# Malate initiates a proton-sensing pathway essential for pH regulation of inflammation

**DOI:** 10.1038/s41392-024-02076-9

**Published:** 2024-12-30

**Authors:** Yu-jia-nan Chen, Rong-chen Shi, Yuan-cai Xiang, Li Fan, Hong Tang, Gang He, Mei Zhou, Xin-zhe Feng, Jin-dong Tan, Pan Huang, Xiao Ye, Kun Zhao, Wen-yu Fu, Liu-li Li, Xu-ting Bian, Huan Chen, Feng Wang, Teng Wang, Chen-ke Zhang, Bing-hua Zhou, Wan Chen, Tao-tao Liang, Jing-tong Lv, Xia Kang, You-xing Shi, Ellen Kim, Yin-hua Qin, Aubryanna Hettinghouse, Kai-di Wang, Xiang-li Zhao, Ming-yu Yang, Yu-zhen Tang, Hai-long Piao, Lin Guo, Chuan-ju Liu, Hong-ming Miao, Kang-lai Tang

**Affiliations:** 1 Department of Orthopedic Surgery/Sports Medicine Center, Southwest Hospital, Army Medical University, Chongqing, 400038 China; 2 Department of Pathophysiology, College of High Altitude Military Medicine, Army Medical University, Chongqing, 400038 China; 3 Department of Orthopedic Surgery, NYU Grossman School of Medicine, New York, NY 10003 USA; 4 NHC Key Laboratory of Diagnosis and Treatment on Brain Functional Diseases & Department of Neurology, The First Affiliated Hospital, Chongqing Medical University, 400016 Chongqing, China; 5 Department of Biochemistry and Molecular Biology, Army Medical University, Chongqing, 400038 China; 6 Department of Biochemistry and Molecular Biology, School of Basic Medical Sciences, Southwest Medical University, Luzhou, Sichuan 646000 China; 7 Department of Rehabilitation Medicine, The First Affiliated Hospital of Chongqing Medical University, Chongqing, 400016 China; 8 Department of Orthopedics and Rehabilitations, Yale University School of Medicine, New Haven, CT 06519 USA; 9 CAS Key Laboratory of Separation Science for Analytical Chemistry, Dalian Institute of Chemical Physics, Chinese Academy of Sciences, Dalian, 116023 China; 10 Department of Anatomy, Engineering Research Center for Organ Intelligent Biological Manufacturing of Chongqing, Key Lab for Biomechanics and Tissue Engineering of Chongqing, Army Medical University, Chongqing, 400038 China; 11 Department of Medical Experimental Center, Qilu Hospital (Qingdao), Cheeloo College of Medicine, Shandong University, Qingdao, 266000 China; 12 Jinfeng Laboratory, Chongqing, 401329 China

**Keywords:** Inflammation, Drug screening, Cell biology

## Abstract

Metabolites can double as a signaling modality that initiates physiological adaptations. Metabolism, a chemical language encoding biological information, has been recognized as a powerful principle directing inflammatory responses. Cytosolic pH is a regulator of inflammatory response in macrophages. Here, we found that L-malate exerts anti-inflammatory effect via BiP-IRF2BP2 signaling, which is a sensor of cytosolic pH in macrophages. First, L-malate, a TCA intermediate upregulated in pro-inflammatory macrophages, was identified as a potent anti-inflammatory metabolite through initial screening. Subsequent screening with DARTS and MS led to the isolation of L-malate-BiP binding. Further screening through protein‒protein interaction microarrays identified a L-malate-restrained coupling of BiP with IRF2BP2, a known anti-inflammatory protein. Interestingly, pH reduction, which promotes carboxyl protonation of L-malate, facilitates L-malate and carboxylate analogues such as succinate to bind BiP, and disrupt BiP-IRF2BP2 interaction in a carboxyl-dependent manner. Both L-malate and acidification inhibit BiP-IRF2BP2 interaction, and protect IRF2BP2 from BiP-driven degradation in macrophages. Furthermore, both in vitro and in vivo, BiP-IRF2BP2 signal is required for effects of both L-malate and pH on inflammatory responses. These findings reveal a previously unrecognized, proton/carboxylate dual sensing pathway wherein pH and L-malate regulate inflammatory responses, indicating the role of certain carboxylate metabolites as adaptors in the proton biosensing by interactions between macromolecules.

## Introduction

The molecular event underlying the prominent plasticity of macrophages is essential for tissue homeostasis during inflammation^1,2^ as well as a promising focus in the exploration of novel anti-inflammatory strategies.^3–8^ Macrophages sense spontaneously and artificially altered physicochemical properties, such as mechanical stress, oxygenation, metabolism, and electrolytes.^9–14^ Certain endogenous metabolites can moonlight as a signaling modality that initiates cellular responses to these chemical dynamisms, particularly pH fluctuations.^15–18^ In innate immune cells, Toll-like receptor (TLR) activations trigger glycolytic reprogramming leading to the accumulation of many metabolites related to tricarboxylic acid (TCA) cycle.^19,20^ Some of these upregulated metabolites trigger protein-mediated signaling pathways, such as Keap1-Nrf2 axis regulated by itaconate, and thereby positively or negatively control the inflammatory response in macrophages.^5,19,21,22^ Although the change in immunoregulatory metabolite during metabolic reprogramming is crucial for the delicate balance of macrophage activation, the role of signaling pathways initiated by these metabolites in the response to environmental cues of macrophages remains incompletely characterized.

Macrophages play a pivotal role in inflammation such as inflammatory response to implant,^23,24^ sepsis,^25^ and inflammatory bowel diseases (IBD).^26^ Regular uses of the acid-suppressive medication have been found to increase the risk of IBD in clinical cohort analysis.^27^ Weaker alkalinity is considered responsible for the anti-inflammatory regulation by nanoflakes on magnesium implants.^28^ The inhibition of the inflammatory response in macrophages induced by exposure to an acidic environment was thought to explain the good tolerance of laparoscopic surgery with CO_2_ exposure.^29–31^ The acidic pH in microenvironment is a hallmark of many inflammatory tissues.^32,33^ In macrophage, TLR4 activation impairs recovery of cytosolic pH in the acidic inflammatory milieu.^34^ CO_2_-induced cytosolic acidification has been found to inhibit the inflammatory cytokines TNF and IL-1 in response to LPS.^29^ Furthermore, the intracellular alkalizers, a Na^+^/H^+^ antiporter, promotes IL-1β production in LPS-activated human monocytes.^35^ These phenomena indicate that pH fluctuations may regulate inflammation and innate inflammatory cytokine through mechanism sensing cytosolic pH, which remains unclear.

In this study, we demonstrated that L-malate, a TCA cycle intermediate, directly binds binding immunoglobulin protein (BiP), a heat shock protein 70 (Hsp70) family member that senses endoplasmic reticulum (ER) stress, disrupts the direct binding of BiP with interferon regulatory factor 2-binding protein 2 (IRF2BP2), a known anti-inflammatory protein.^36,37^ In macrophages, L-malate inhibits BiP-IRF2BP2 interaction, protects IRF2BP2 from BiP-driven degradation, and thereby suppresses inflammatory responses in vitro and in vivo. Interestingly, pH reduction, which promotes carboxyl protonation of L-malate, facilitates L-malate and carboxylate analogues to bind BiP and to restrain BiP-IRF2BP2 interaction in a carboxyl-dependent manner. In macrophages, BiP-IRF2BP2 interaction senses intracellular pH (pH_i_) around 7.0 independently of lysosome pH. Analysis of BiP-malate binding suggested the potential carboxyl-carboxyl pair formed between BiP and L-malate as a “pKa-increasing” chemical basis^38^ for an adequate proton-sensitivity of BiP-IRF2BP2 interaction under pH around 7.0. BiP-IRF2BP2 signaling pathway in macrophages is sensitive to pH fluctuations, and is required for the anti-inflammatory of acidification as well as the pro-inflammatory effect induced by alkalizers of intracellular pH. Furthermore, local alkalization in mouse colons induced colitis which is dependent on myeloid-specific BiP. This alkalization in colons also decreases the protein level of IRF2BP2, whose physiological distribution appears negatively correlated with environmental pH. Collectively, our study reveals that an anti-inflammatory BiP-IRF2BP2 signaling pathway which senses L-malate and cytosolic protons is required for the regulation of macrophage IL-1β production by pH, and indicates a role of certain carboxylate metabolites with various pKa values (pH sensitivities) as an adaptor in proton biosensing by protein-protein interaction.

## Results

### L-malate supplementation exerts anti-inflammatory effects in vitro

**Fig. 1:**
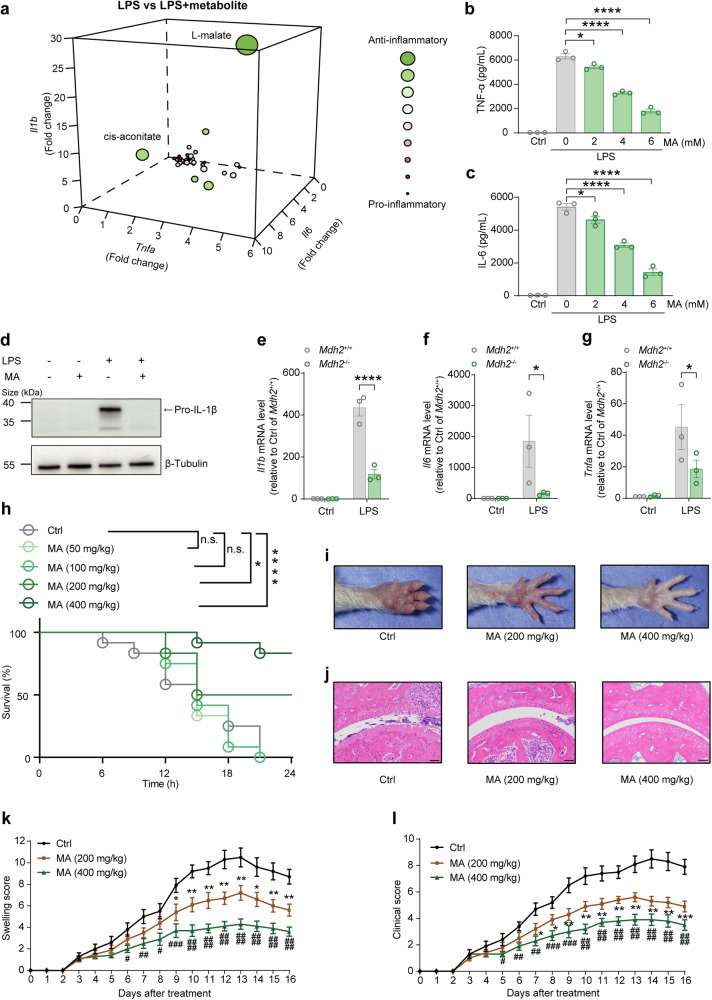
L-malate acts as an anti-inflammatory metabolite. (**a**) Three–dimensional plot coordinates are generated from fold change of *Il1b*, *Tnfa*, and *Il6* expressions in LPS stimulated (24 h) BMDMs treated with vehicle versus that treated with each metabolite, and dot sizes indicate the product of these three fold changes (See also Fig. S1 for details). (**b**-**d**) LPS-stimulated (24 h) BMDMs treated with L-malate (MA) as indicated and extracellular levels of TNF-α (**b**) and IL-6 (**c**) measured by ELISA and intracellular pro-IL-1β abundance visualized by immunoblotting (**d**). (**e**–**g**) *Il1b* (**e**), *Il6* (**f**), *Tnfa* (**g**) mRNA expressions measured by qPCR in *Mdh2*^−/−^ and WT Raw264.7 cell lines under LPS stimulation (24 h). (**h**) Survival rates of WT mice (n = 12) administrated intragastrically with different dosages of L-malate (50, 100, 200 and 400 mg/kg) and injected intraperitoneally with LPS. (**i**) Representative paws of CAIA mice (day 14) treated with or without L-malate. Scale bars, 50μm. (**j**) Representative H&E staining of paw of CAIA mice (day 14) treated with or without L-malate. (**k**, **l**) Swelling score (**k**) and clinical score (**l**) in CAIA mice intraperitoneally injected with different dosages of L-malate (200 and 400 mg/kg, n = 10). n ≥ 3. The data are shown as the mean ± SEM. In (b, c, e – g, h), *p < 0.05; **p < 0.01, ***p < 0.001, ****p < 0.0001. In (**k** and **l**); * < 0.05, ** < 0.01 [MA (200 mg kg^-1^) compared to Ctrl]; # < 0.05, ## < 0.01, ### < 0.001, #### < 0.0001 [MA (400 mg kg^-1^) compared to Ctrl] (unpaired Student’s *t*-test, one-way ANOVA, two-way ANOVA or Mantel‒Cox survival analysis). See also Supplementary Fig. 1-5

Initially, we aimed to broaden the toolbox of anti-inflammatory therapeutic strategies with endogenous compounds including intermediates and the derived metabolites in TCA cycle. Thirty-eight energy metabolic intermediates with accessible solubility were screened with bone marrow-derived macrophages (BMDMs) stimulated by lipopolysaccharide (LPS), and the expression levels of *Il1b*, *Tnfa*, and *Il6* were then measured by qPCR. L-malate, a TCA intermediate, stood out as a potently anti-inflammatory candidate (Fig. 1a and Supplementary Fig. 1a-o). We next verified the anti-inflammatory effect of L-malate on IL-6 (Fig. 1b) and TNF-α (Fig. 1c) by ELISA and on the precursor of IL-1β (pro-IL-1β) using Western blotting (Fig. 1d). Additionally, L-malate treatment induced robust alteration of transcriptional program in LPS-stimulated BMDMs, resulting in the identification of 968 differentially expressed genes (p < 0.05, |fold-change| >2597 upregulated and 371 downregulated) (Supplementary Fig. 2a). KEGG analysis of the 968 differentially expressed genes revealed enrichment in cytokine‒cytokine receptor interaction, inflammatory bowel disease (IBD), and rheumatoid arthritis (Supplementary Fig. 2b). Altogether, these data demonstrated that L-malate supplementation inhibits the pro-inflammatory response in activated macrophages.

### L-malate supplementation alleviates inflammation in vivo

Furthermore, we tested whether L-malate suppressed inflammation in several animal models of inflammatory diseases based on clues collected from RNA-seq analysis mentioned above (Supplementary Fig. 2b). Mice receiving a lethal dose of LPS succumbed to LPS shock within 24 h, whereas mice administered with both LPS and a single dose of L-malate at 200 or 400 mg/kg body weight showed an increased survival rate (Fig. 1h). We next examined the effect of L-malate on inflammatory arthritis in collagen antibody-induced arthritis (CAIA) model. The administration of L-malate alleviated the disease severity including paw swelling (Fig. 1i, k), joint destruction (Fig. 1j), clinical score (Fig. 1l), and the joint levels/proportions of pro-inflammatory cytokines/macrophages (Supplementary Fig. 2c, d) in mice with CAIA. We also determined the anti-inflammatory effect of L-malate supplementation in inflammatory bowel disease using the acute dextran sulfate sodium salt (DSS) model of colitis, the results revealed that L-malate-treated mice with DSS-induced colitis displayed the reduction in disease activity (Supplementary Fig. 3a), colon length shortening (Supplementary Fig. 3c), intestinal inflammation (Supplementary Fig. 3b), the levels of pro-inflammatory mediators in colon (Supplementary Fig. 3d, e), and the proportions of pro-inflammatory macrophages in colon, blood and spleen (Supplementary Fig. 3f). In sum, L-malate supplementation exhibited anti-inflammatory effects in various animal models of inflammation.

### L-malate acts as an intracellular anti-inflammatory metabolite with physiological relevance

We next analyzed the mechanism of anti-inflammatory effect induced by L-malate addition. Apparently, L-malate supplementation can lead to both acidification and accumulation of L-malate structure. Acidized medium (AcM) (pH 6.7) alone exhibited an anti-inflammatory effect which was milder than that of L-malate addition (6 mM, medium pH 6.7) (Supplementary Fig. 4a, b). A recent study demonstrated that GPR65, a sensor of extracellular pH (pH_e_), regulates adaptive immune response and tissue inflammation in mice models of inflammatory and infectious diseases,^39^ and another study considered that GPR65 partially explains the regulation of IL-6 and TNF-α in macrophages under acidic microenvironments.^40^ Unexpectedly, we found that H89, an inhibitor of GPR-cAMP-PKA signaling, did not affect the anti-inflammatory activity of L-malate supplementation (Supplementary Fig. 4c) in LPS-stimulated macrophages. These results prompted us to test the role of pH_i_ in the anti-inflammatory effect of malate addition. We then used monensin, a plasma membrane Na^+^/H^+^ antiporter that alkalinizes the pH_i_ independently of pH_e,_^15^ and found that the AcM-induced inhibition of *Il1b* is blocked by monensin (Supplementary Fig. 4d). Further, Monensin also impaired the effect of L-malate additions on LPS-induced *Il1b* expressions either in uncontrolled pH (Supplementary Fig. 4e), or in the pH_e_-controlled condition (Supplementary Fig. 4f). Notably, to preclude the role of Na^+^ transport in Monensin’s effects, we used sodium acetate, a pH_i_ acidifier without the opposite impact on sodium transport with monensin, and found that sodium acetate rescued the impairment induced by monensin (Supplementary Fig. 4f). However, among the candidates screened as shown above, the acidity of L-malate was not as prominent as its anti-inflammatory effect^15^ (Supplementary Fig. 4g), indicating a specific effect of L-malate compounds. In addition to the finding that L-malate supplement (medium pH 6.7) further inhibited IL-1β productions compared with AcM (pH 6.7) (Supplementary Fig. 4a, b), we examined whether an endogenous increase in L-malate regulates the inflammatory response. We knocked out malate dehydrogenase 2 (*Mdh2*), which catalyzes the oxidation of L-malate to oxaloacetate, in Raw264.7 cell line and found that *Mdh2* knockout potently increased the intracellular levels of malate (Supplementary Fig. 4h) and impaired LPS-induced expressions of pro-inflammatory cytokines (Fig. 1e–g). Notably, in LPS-stimulated macrophages, *Mdh2*-deficiency did not affect the pH_i_ (Supplementary Fig. 4i) but inhibited *Il1b* expressions in an alkalizer-irreversible manner (Supplementary Fig. 4j). Overall, these data indicated that added L-malate structure and reduced pH_i_ are both responsible for the anti-inflammatory activity of L-malate supplementation, and suggested a potential interaction between the effects of L-malate structure and intracellular protons on IL-1β, which is further analyzed in later paragraph.

We next determined whether extracellularly applied L-malate enters cells. When pH goes down, certain dicarboxylate can be sensitively protonated, be transformed from a dicarboxylate to a monocarboxylate.^15^ L-malate and succinate share the high monocarboxylic pKa (Supplementary Fig. 4g), which can increase the cell permeability of the dicarboxylate, which is blocked by cell membrane, via monocarboxylate transporter 1 (MCT1) of skeletal muscle cells.^15^ Moreover, in macrophages, MCT1 is the major influx MCT that can be upregulated by LPS.^17,41^ We thus speculated that exogenous applied L-malate can be transported into the macrophages through MCT1. As expected, L-malate supplementation induced the accumulation of intracellular L-malate in the peripheral blood mononuclear cells (PBMCs) of mice, and this effect can be abolished by MCT1 inhibitor AZD3965 (Supplementary Fig. 5a). Also, after adding deuterium-labeled L-malate into the medium (pH 6.9) of LPS-stimulated BMDMs, we detected the increase in hydrogen isotope ratios of malate, fumarate and aspartate (Supplementary Fig. 5b). L-malate addition increased the intracellular levels of malate in macrophages (Supplementary Fig. 5c), and consistently with the limited dicarboxylate transport of MCTs in non-acidic pH,^15^ although not leading to a change in endogenous malate levels (Supplementary Fig. 5d), loss of acidic pH_e_ ablated the upregulation of intracellular malate induced by L-malate addition (Supplementary Fig. 5c). Collectively, these data suggested that extracellularly applied L-malate enters the cells and increases the intracellular levels of malate. Notably, while L-malate structure takes anti-inflammatory effect (Supplementary Fig. 4a, b, f) without reducing pH_e_, loss of acidic pH_e_ impaired the effect of L-malate treatment on both intracellular malate (Supplementary Fig. 5c) and inflammatory responses (Supplementary Fig. 5e–g), which indicates that the accumulation of intracellular malate is required for the anti-inflammatory effect of L-malate treatment and suggests a relative selectivity in the uptake of exogenously applied L-malate into cells of inflammatory tissue characterized by acidic pH_e_.

Only a moderate foldchanges (~2–3 folds baselines) were measured in intracellular malate levels after L-malate supplementation in LPS-stimulated macrophages (Supplementary Fig. 5h). Subsequently, we inquired whether intracellular L-malate exerts anti-inflammatory effect with physiological relevance. The concentrations of L-malate are reported to be ~200 μM^42^ in cells and intracellular malate accumulated to 2-3x baseline during LPS-induced glycolytic reprogramming in proinflammatory macrophages^19,21^ (Supplementary Fig. 5i). Next, we reduced the concentrations of supplementary L-malate to 1.5 mM with less medium acidity (pH 6.9). We also found the inhibition of IL-1β levels induced by L-malate (1.5 mM, pH_e_ 6.9) (Supplementary Fig. 5j–l). Considering the entry of exogenous L-malate could be limited in pH 6.9 if we further reduce the concentration of added L-malate, we lowered the medium pH to 6.8, and used NH_4_Cl as a pH_i_ alkalizer to counterbalance the side effects of an acidic medium on the pH_i_. Under this condition, we detected a significant anti-inflammatory effect of 500 μM malate supplementation (Supplementary Fig. 5m–o). Moreover, MCT1 inhibitors blocked the downregulation of *Il1b* mRNA levels induced by 500 μM L-malate addition in LPS-primed macrophages (Supplementary Fig. 5p–r). Collectively, these data demonstrated that L-malate acts as an anti-inflammatory signal inside cells.

### L-malate directly binds BiP

We then focused on the mechanism underlying the anti-inflammatory effect of L-malate structure. It has been widely reported that the L-malate shuttle is a critical mechanism in regulating redox homeostasis by transporting reducing equivalents in eukaryotic cells, and L-malate reportedly increases the generation of reactive oxygen species (ROS) in HeLa cells.^43^ However, ROS induced by succinate oxidation are known to act as a pro-inflammatory factor in BMDMs treated with LPS,^21^ suggesting that the increase in ROS production is unlikely to explain the anti-inflammatory activity of L-malate. Second, it is known that L-malate can be reversibly converted into fumarate, a well-established anti-inflammatory metabolite that targets glyceraldehyde-3-phosphate dehydrogenase (GAPDH).^6^ To test the possibility that L-malate inhibits inflammation through the secondary accumulation of fumarate, we used FHIn1, a fumarate hydratase inhibitor that interrupts the shift between L-malate and fumarate in the TCA cycle, and Heptelidic Acid, a GAPDH inhibitor, did not influence the anti-inflammatory effect of L-malate (Supplementary Fig. 6a–f), even though their known inhibitory effects were observed (Supplementary Fig. 6g, h). These results indicated that the regulatory effects of L-malate on inflammation are independent of the secondary accumulation of fumarate or the target of fumarate. It is also well-known that glycolysis is necessary for LPS-induced *Il1b* expressions.^19^ However, L-malate addition did not significantly affect glycolysis in LPS-stimulated macrophages (Supplementary Fig. 6i). Collectively, these data indicated that L-malate inhibits inflammatory response independently of metabolism.

**Fig. 2:**
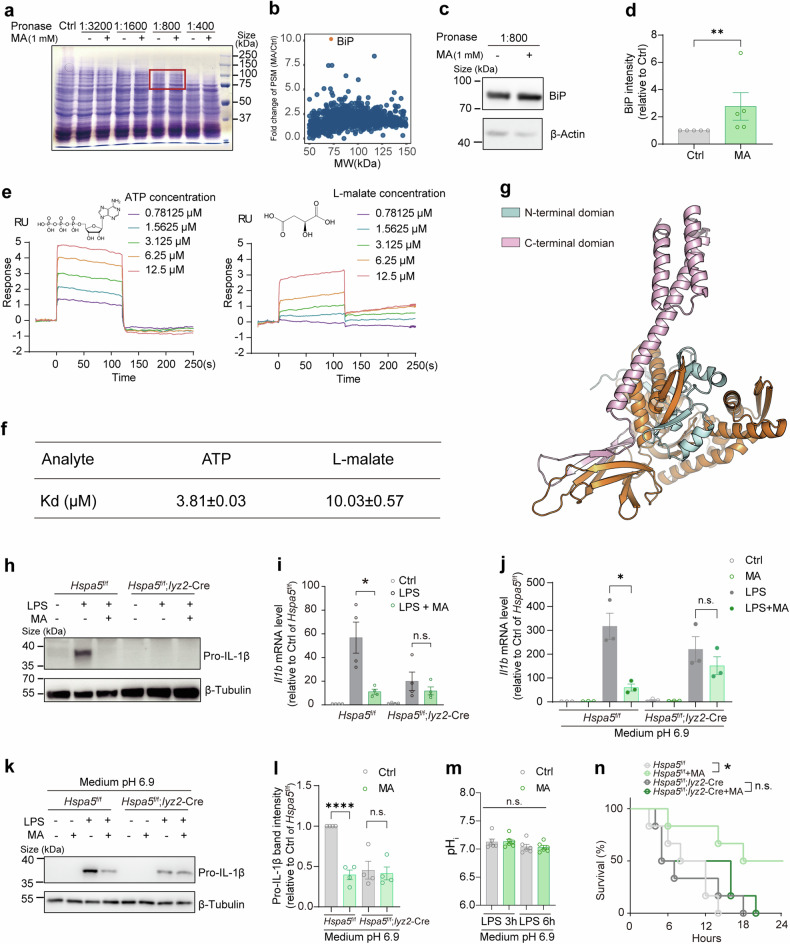
BiP binds L-malate and is required for its anti-inflammatory effect. (**a**) Coomassie blue staining of SDS-PAGE gel for Raw264.7 cell lysates which were digested with protease with or without L-malate (1 mM, pH 7.0) according to DARTS assay. The bands protected by L-malate (1 mM, pH 7.0) was indicated by red frame. Representative data from three experiments. (**b**) Mass spectrometry analysis revealed the enrichment of BiP protein (shown as orange dots) in the protected bands from the L-malate-treated cell lysate in DARTS assays. (**c** and **d**) Raw264.7 cell lysates were digested with protease with or without L-malate (1 mM, pH 7.0) according to DARTS assay, then the levels of BiP protein was assayed using Western blot (**c**) and quantified (**d**). (**e** and **f**) BIAcore diagram showing responses measured in resonance unit (RU) of E. coli-expressed BiP protein (chip-coupled) to ATP or L-malate (pH 7.4). Representative data from three experiments (e); K_D_ for each interaction of BiP with ATP and L-malate is indicated (**f**). (**g**) Crystal structure of BiP (PDB: 5E84) with different regions colored by palecyan (aa 1-125), orange (aa 126-499), and palepink (aa 500-654). (**h** and **i**) *Il1b* mRNA expressions quantified by qPCR (**h**) and pro-IL-1β protein abundance visualized by immunoblotting (**i**) in BMDMs isolated from *Hspa5*^f/f^ or *Hspa5*^f/f^; *Lyz2*-Cre mice. These BMDMs were treated with or without L-malate (6 mM, pH 6.7), under stimulation of LPS for 24 h. (**j**, **k** and **l**) *Il1b* mRNA expressions quantified by qPCR (**j**) and pro-IL-1β protein abundance visualized (**k**) and quantified (**l**) by immunoblotting in BMDMs treated with or without L-malate (6 mM) in medium pH 6.9, under stimulation of LPS for 16 h. In addition, BMDMs were isolated from *Hspa5*^f/f^ or *Hspa5*^f/f^; *Lyz2*-Cre mice. (**m**) pH_i_ of in BMDMs treated with or without L-malate (6 mM) in medium pH 6.9, under stimulation of LPS for 3-6 h. (**n**) Survival rate in *Hspa5*^f/f^ or *Hspa5*^f/f^; *Lyz2*-Cre mice (n = 6) administrated intragastrically with L-malate (400 mg kg^−1^) and injected intraperitoneally LPS. n ≥ 3. The data are shown as the mean ± SEM. *p < 0.05; **p < 0.01, ***p < 0.001, ****p < 0.0001. (two-sided Mann‒Whitney U test, unpaired Student’s *t*-test, Mantel‒Cox survival analysis). See also Supplementary Fig. 6–8

Consequently, we attempted to disclose the potential unidentified molecular basis underlying the anti-inflammatory effect of L-malate. We leveraged an unbiased biochemical approach, drug affinity responsive target stability (DARTS).^44–46^ Raw264.7 macrophages were employed as the protein source for DARTS, and the bands protected by L-malate treatment during protease digestion were detected by Coomassie blue staining (Fig. 2a). Mass spectrometry identified binding immunoglobulin protein (BiP, also referred to as ER chaperone Grp78, 78-kDa glucose regulated protein, and HspA5, heat shock protein A5) as a potently protected protein present in the L-malate-treated protein sample (Fig. 2a–d). Surface plasmon resonance (SPR) detected a direct interaction of L-malate with commercial recombinant BiP protein (Fig. 2e, f) or self-produced, full-length BiP without the signal peptide (aa 25-654) (Supplementary Fig. 8a, b). Adenosine triphosphate (ATP), which is known to bind BiP,^47,48^ was used as a positive control in our SPR assay (Fig. 2e, f). In sum, these results demonstrated that L-malate directly binds BiP, and prompted us to test the role of BiP in L-malate’s anti-inflammatory activities.

### BiP is required for anti-inflammatory effects of L-malate

In activated macrophages, BiP deficiency drastically downregulated the pro-IL-1β at the late stage of LPS stimulation (24 h) (Fig. 2h), and blocked the inhibitions of *Il1b* levels induced by L-malate addition (Fig. 2i) and by L-malate under pH_e_-controlled condition (medium pH 6.9) (Fig. 2j-l), in which L-malate entered cells (Supplementary Fig. 5b) but did not reduce pH_i_ (Fig. 2m). To assess the role of BiP in the therapeutic effect on inflammatory response of L-malate administration in vivo, we evaluate the impact of myeloid BiP-deficiency on L-malate-treated mice receiving a lethal dose of LPS, and found that the increase in the survival rate induced by L-malate administration was blocked in *Hspa5*^f/f^; *Lyz2*-Cre mice (Fig. 2n). These data suggested that BiP is the main downstream mediator of L-malate in its regulation of inflammation. We next aimed to gain insights into how BiP mediates L-malate induced inhibition of inflammation. We first considered the critical role of BiP in ER stress (ERS) and unfolded protein response (UPR) activity.^49^ The UPR is reportedly a linker of metabolic alterations and the inflammatory signature.^20^ We performed RNA sequencing of BMDMs treated with LPS plus L-malate or those treated with LPS for 24 h. The GO analysis and heatmap indicated alternations of UPR signals in L-malate-treated group (Supplementary Fig. 7a, b). Thus, we sought to determine whether the anti-inflammatory activity of L-malate addition is dependent on three traditional UPR signal transducers, ATF6, PERK, and IRE1α.^50–52^ Unexpectedly, the inhibition of L-malate on LPS-induced inflammatory signals was not affected by 4μ8c (Supplementary Fig. 7c–e), GSK2606414 (Supplementary Fig. 7f–h), and Ceapin-A7 (Supplementary Fig. 7i–k), which are corresponding inhibitors that impaired effects of L-malate on three UPR signaling branches, IRE1α (Supplementary Fig. 7l), PERK (Supplementary Fig. 7m, n) and ATF6 (Supplementary Fig. 7o, p), respectively. These findings suggested that the anti-inflammatory activity of L-malate is independent of the traditional UPR pathway and led us to propose that the L-malate-BiP axis may regulate inflammation through a previous unknown mechanism.

### BiP directly binds IRF2BP2 while L-malate restrains BiP-IRF2BP2 interaction

With the aim of filling the mechanistic gap between inflammation modulation and the L-malate-BiP axis, we analyzed the binding mode of L-malate and BiP by generating serial BiP fragments to identify the domain of BiP that binds L-malate by SPR (Supplementary Fig. 8a). We found that L-malate bound to fragments that includes either N-terminal or C-terminal BiP domain (Supplementary Fig. 8b–g, k). Conversely, the binding of L-malate and the BiP fragments without these two L-malate-binding domains was not detected (Supplementary Fig. 8h–j). Interestingly, according to X-ray crystallographic data of BiP, its L-malate-binding regions, the N-terminal and C-terminal domains, are spatially close^48^ (Fig. 2g), suggesting a conformational change in BiP caused by the binding of L-malate between these two domains. Regretfully, due to the limitation of phenotypic studies on the amino acids within the L-malate-BiP binding area, as well as the complexity of the overall binding mode, it is difficult to predict the functional consequence of L-malate-BiP coupling merely by analyzing their binding sites. Thus, we decided to seek additional clues from known features of protein-metabolite interactions and BiP-related ligand binding.

**Fig. 3:**
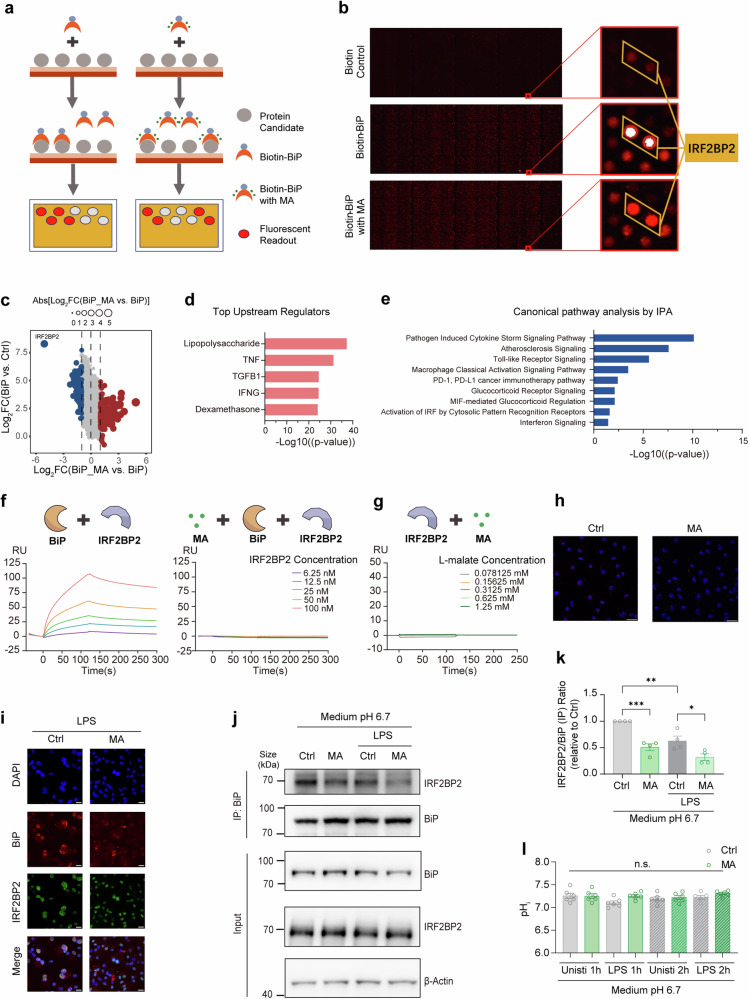
BiP and IRF2BP2 show direct binding affinity which is impaired by L-malate. (**a**) Strategy for identifying L-malate-responding protein-protein interactions with BiP. (**b**) Overall and notable readouts in the protein-protein interaction microarrays. (**c**) Scatterplot of signals from the protein microarrays of the BiP, BiP + L-malate (1 mM, pH adjusted to 7.2) and control groups. Proteins whose interaction with BiP were detected to be inhibited by L-malate are shown as blue dots, while proteins whose interaction with BiP were detected to be promoted by L-malate are shown as red dots. Size of circle indicates the absolute value of the logarithm of the ratios of the binding signal in the BiP+MA-treated group to the binding signal in the BiP-treated group to base two. In addition, protein IRF2BP2 was labeled. (**d**) Top predicted upstream regulators of DEGs analyzed by RNA-seq in BMDMs treated with LPS compared to BMDMs treated with LPS plus L-malate (24 h). (**e**) Canonical pathway analysis of the DEGs from RNA-seq in BMDMs treated with LPS compared to that treated with LPS plus L-malate (12 h), which of interest were shown. (**f**) BIAcore diagram of IRF2BP2 (concentrations indicated with colored lines) and BiP (chip-coupled) with or without L-malate (6 mM, pH 7.4). (**g**) BIAcore diagram of IRF2BP2 (chip-coupled) and L-malate (pH 7.4, concentrations indicated with colored lines). (**h**) The red spots represent BiP-IRF2BP2 bindings detected by PLA assay in BMDMs treated with or without L-malate (6 mM, pH 6.7), under stimulation of LPS for 3 h. Nuclei are stained with DAPI. Scale bars, 20 μm. Representative data from three experiments. (**i**) Immunofluorescent staining showing BiP-IRF2BP2 co-localizations in LPS-stimulated BMDMs treated with or without L-malate (6 mM, pH 6.7) for 1 h. Nuclei are stained with DAPI. Scale bars, 10 μm. Representative data from three experiments. (**j** and **k**) Cellular BiP-IRF2BP2 interactions shown by Western blot analysis of co-immunoprecipitation in MG132-pretreated (5 μM, 0.5 h) Raw264.7 cell lines treated with MG132 (0.5 μM) plus L-malate (6 mM) for 3 h in the medium pH 6.7. (**l**) pH_i_ of MG132-pretreated (5 μM, 0.5 h) Raw264.7 cell lines treated with MG132 (0.5 μM) plus L-malate (6 mM) for 1-2 h in the medium pH 6.7. n ≥ 3. The data are shown as the mean ± SEM. *p < 0.05; **p < 0.01, ***p < 0.001. (two-sided Mann‒Whitney U test, unpaired Student’s *t*-test, Mantel‒Cox survival analysis). See also Supplementary Fig. 9

It has been revealed that a subset of proteins shows responses to certain metabolites which are indicative of structural alterations, and metabolite-induced structural rearrangements coexist with alterations in the protein complex.^53–55^ Notably, the chaperone function of Hsp70s is mainly mediated by the interaction of its C-terminal substrate-binding domain (SBD) with extended hydrophobic polypeptides whose affinity and kinetics could be allosterically modulated by the binding of metabolites.^47,48,56–58^ Notably, the aa500-640 segment of BiP detected to bind with L-malate mainly includes the SBDα subdomain (aa501-636) of SBD, which is able to bind hydrophobic regions of substrate proteins.^48^ Based on our SPR data and these general patterns mentioned above, we speculate that L-malate binding could induce a conformational change in BiP, which results in the regulation of inflammatory response. Therefore, we examined whether L-malate affects the protein‒protein interaction of BiP. Considering the complexity and wide range of BiP interactomes, we performed another unbiased assay, human protein microarrays, which contain ~20,000 full-length, purified human proteins expressed in yeast.^59,60^ The “binding signal” of each candidate with three different treatments, the vehicle and the recombinant BiP protein with or without L-malate, was tested (Fig. 3a, b). The protein candidate showing the strongest “BiP-binding signal” was interferon regulatory factor 2–binding protein 2 (IRF2BP2), and meanwhile the “BiP-binding signal” of IRF2BP2 was most sensitive to L-malate (Fig. 3b, c). Importantly, IRF2BP2 is an anti-inflammatory protein in macrophages,^36^ which inhibits *Il1b* expressions.^37^

Moreover, based on *in cellua* assays, we performed complementary analyses to assess how L-malate affects molecular regulation in LPS-stimulated BMDMs through an Ingenuity Pathway Analysis (IPA) of RNA-seq data, which showed that “dexamethasone” was one of the top 5 predicted upstream regulators of transcriptional alterations (Fig. 3d), and a canonical pathway analysis revealed that the identified differentially expressed genes (DEGs) in RNA-seq were involved in “PD-1/PD-L1 cancer immunotherapy pathway” and “glucocorticoid receptor signaling” (Fig. 3e). Notably, IRF2BP2 is considered as a coregulator of glucocorticoid receptor (GR) and NF-κB^61,62^ and reportedly regulates PD-L1 expressions in tumors.^63^

Based on the amino acid sequence of IRF2BP2, we predicted its structure using RoseTTAFold, and based on the evaluation results and error estimate values, the Profiles-3D scores and the Ramachandran plot, model 1 was ultimately selected (Supplementary Fig. 9a–g) for protein‒protein docking with the crystal structure of BiP using ZDOCK. This docking model (Supplementary Fig. 9i, j) showed an overlapping binding area with the interaction between BiP and L-malate (Fig. 2g, Supplementary Fig. 8g, k), further indicating the critical role of L-malate in the BiP-IRF2BP2 interaction.

We then verified the L-malate-sensitive interaction between BiP and IRF2BP2 through multiple approaches. In SPR, we detected direct bindings between BiP and IRF2BP2 and L-malate-induced disruption of BiP-IRF2BP2 binding (Fig. 3f), while not detect bindings of L-malate with IRF2BP2 (Fig. 3g). In LPS-primed macrophages, we also detected the inhibitory effect of L-malate treatment on BiP-IRF2BP2 interaction through proximal ligation assays (Fig. 3h) and on BiP-IRF2BP2 co-localization via immunofluorescence (Fig. 3i). Notably, although BiP is mainly located in the endoplasmic reticulum lumen, BiP retro-translocates from ER through its Nt-arginylation.^64,65^ Through co-immunoprecipitation (Co-IP) in both LPS-stimulated and unstimulated macrophages, we found that L-malate reduced BiP-IRF2BP2 interactions (Fig. 3j, k) but not pH_e_ (controlled medium pH 6.7) or pH_i_ (Fig. 3l). Consistently with the accumulation of intracellular L-malate during LPS stimulation^19,21^ (Supplementary Fig. 5i), LPS suppressed intracellular interactions of BiP with IRF2BP2 (Fig. 3j, k). Taken together, these data demonstrate that BiP directly binds IRF2BP2 and that L-malate inhibits the BiP-IRF2BP2 interaction.

### L-malate-BiP axis targets IRF2BP2 proteolysis to regulate inflammatory response

**Fig. 4:**
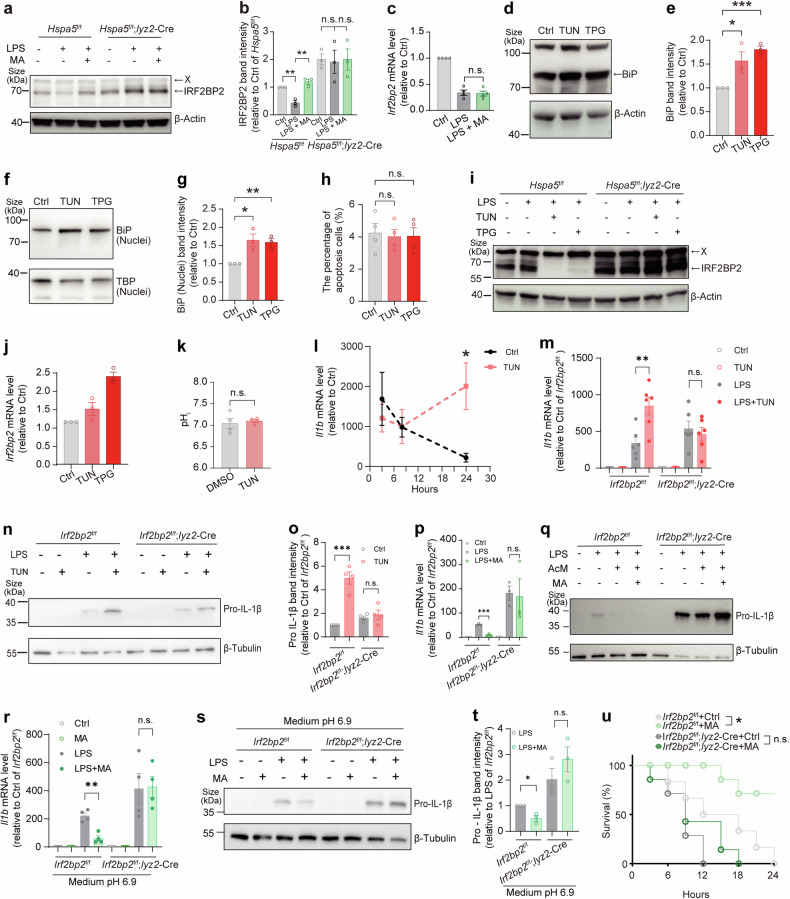
L-malate-BiP axis regulates the inflammatory response through IRF2BP2. (**a**, **b**) IRF2BP2 protein levels was visualized by immunoblotting (**b**) and quantification (**c**) of IRF2BP2 in BMDMs from *Hspa5*^f/f^ or *Hspa5*^f/f^; *Lyz2*-Cre mice. These LPS-stimulated BMDMs were incubated with/without L-malate (6 mM, medium pH 6.7, 8 h). (**c**) *Irf2bp2* mRNA expressions quantified by qPCR in LPS-stimulated BMDMs treated with or without L-malate (6 mM, medium pH 6.7, 8 h). (**d**-**g**) BiP protein levels of whole-cell (**d**, **e**) or nuclei (**f**, **g**) of BMDMs treated with/without Tunicamycin (TUN) (0.1 μM) or Thapsigargin (TPG) (0.1 μM) for 16 h. (**h**) Apoptosis rates detected by flow cytometry of BMDMs treated with/without Tunicamycin (0.1 μM) or Thapsigargin (0.1 μM) under 24 h stimulation of LPS. In addition, Tunicamycin (0.1 μM) or Thapsigargin (0.1 μM) was added 16 h before LPS stimulation. (**i**) IRF2BP2 protein abundance visualized by immunoblotting in BMDMs from *Hspa5*^f/f^ or *Hspa5*^f/f^; *Lyz2*-Cre mice. These BMDMs were incubated with/without Tunicamycin (0.1 μM) or Thapsigargin (0.1 μM) and stimulated with LPS (2 h). In addition, Tunicamycin (0.1 μM) or Thapsigargin (0.1 μM) was added 16 h before LPS stimulation. Representative data from three experiments. (**j**) *Irf2bp2* mRNA levels measured by qPCR in BMDMs isolated from *Hspa5*^f/f^ or *Hspa5*^f/f^; *Lyz2-Cre* mice. These BMDMs were stimulated with LPS (2 h) and incubated with/without Tunicamycin (0.1 μM) or Thapsigargin (0.1 μM) which were added 16 h before LPS stimulation. (**k**) pH_i_ of BMDMs incubated with/without Tunicamycin (0.1 μM) for 16 h. **(l)** *Il1b* mRNA levels in BMDMs stimulated with LPS (3 h, 8 h, 24 h) and incubated with/without Tunicamycin (0.1 μM) or Thapsigargin (0.1 μM) which were added 16 h before LPS stimulation. (**m**-**o**) *Il1b* mRNA levels quantified by qPCR (m) and pro-IL-1β protein levels visualized (**n**) and quantified (**o**) by immunoblotting in BMDMs isolated from *Irf2bp2*^f/f^ or *Irf2bp2*^f/f^; *Lyz2*-Cre mice. These BMDMs were incubated with/without Tunicamycin (0.1 μM) or Thapsigargin (0.1 μM) and stimulated with LPS (16 h). In addition, Tunicamycin (0.1 μM) or Thapsigargin (0.1 μM) was added 24 h before LPS stimulation. (**p**, **q**) *Il1b* mRNA (**p**) and pro-IL-1β protein abundance (**q**) in BMDMs isolated from *Irf2bp2*^f/f^ or *Irf2bp2*^f/f^; *Lyz2*-Cre mice treated with medium (pH 6.7) or L-malate (6 mM, medium pH 6.7), under stimulation of LPS for 24 h. (**r**-**t**) *Il1b* mRNA (**r**) and pro-IL-1β protein levels (**s** and **t**) in BMDMs isolated from *Irf2bp2*^f/f^ or *Irf2bp2*^f/f^; *Lyz2*-Cre mice. These BMDMs under a controlled medium pH (6.9) were treated with or without L-malate (6 mM), under stimulation of LPS for 16 h. **(u)** Survival in *Irf2bp2*^f/f^ or *Irf2bp2*^f/f^; *Lyz2*-Cre mice intragastric administrated with L-malate (400 mg kg^−1^) and intraperitoneally injected LPS (n = 6-7 per group). The “X” means unknown proteins detected in immunoblotting visualizing IRF2BP2. *n* ≥ 3. Data are shown as mean ± SEM. *p < 0.05; **p < 0.01; ***p < 0.001 (unpaired Student’s *t*-test, Mantel-Cox survival analysis). See also Supplementary Figs. 6 and 8

We then decided to examine whether IRF2BP2 mediates the effect of L-malate-BiP axis on the inflammatory response. We first test the potential posttranslational regulation of IRF2BP2 by L-malate and BiP. It has been reported that IRF2BP2 can be strongly phosphorylated, and its nuclear localization depends on phosphorylation.^66,67^ However, in LPS-stimulated macrophages whose BiP-IRF2BP2 co-localization was found to be reduced by L-malate addition, most of IRF2BP2 appeared to be co-localized with nuclear marker and no obvious difference in IRF2BP2 localization was observed after L-malate treatment (Fig. 3i). Subsequently, we tested effects of L-malate on IRF2BP2 protein abundance during LPS stimulation. We found that the protein levels of IRF2BP2 were increased by L-malate addition in a BiP-dependent manner (Fig. 4a, b), while the mRNA levels of *Irf2bp2* was not affected by L-malate treatment (Fig. 4c) in LPS-primed macrophages. Next, we used Tunicamycin and Thapsigargin, as BiP inducers in macrophages,^68^ at a lower dosage than where they were used as an ER stressor and found that these treatments increased the protein levels of BiP in both the whole cell (Fig. 4d, e) and the nuclei (Fig. 4f, g) but had no significant effect on cell death (Fig. 4h). Both Thapsigargin and Tunicamycin drastically decreased the protein abundance of IRF2BP2 in LPS-stimulated macrophages in a BiP-dependent manner (Fig. 4i), while not decreasing the mRNA levels of *Irf2bp2* (Fig. 4j). Notably, although Thapsigargin reportedly leads to intracellular acidification,^69^ Tunicamycin were not detected to affect pH_i_ (Fig. 4k).

Furthermore, to examine the phenotypic relationship between BiP and IRF2BP2, we tested the regulation of IL-1β by the BiP inducer in inflammatory macrophages and found that Tunicamycin promoted *Il1b* expressions in activated macrophages (Fig. 4l), and IRF2BP2 deficiency abrogated promotive effects of Tunicamycin in *Il1b* expressions (Fig. 4m) and pro-IL-1β production (Fig. 4n, o). Thus, we propose that BiP regulates *Il1b* expressions in an IRF2BP2-dependent manner. We next examined the role of IRF2BP2 in the anti-inflammatory regulation of L-malate. In LPS-primed macrophages, IRF2BP2 deficiency blocked the anti-inflammatory effect of L-malate addition (Fig. 4p, q) and of L-malate under pH-controlled condition (Fig. 4r–t), in which L-malate entered cells (Supplementary Fig. 5b) but did not reduce pH_i_ (Fig. 2m). Further, we ascertained whether IRF2BP2 play a role in the protective effect on inflammatory response of L-malate treatment in vivo, and found that the increase in survival rates induced by L-malate administration was abolished in *Irf2bp2*^f/f^; *Lyz2-*Cre mice (Fig. 4u). Collectively, these results demonstrate that L-malate-BiP axis regulates inflammatory responses through the BiP-bound anti-inflammatory protein IRF2BP2.

**Fig. 5:**
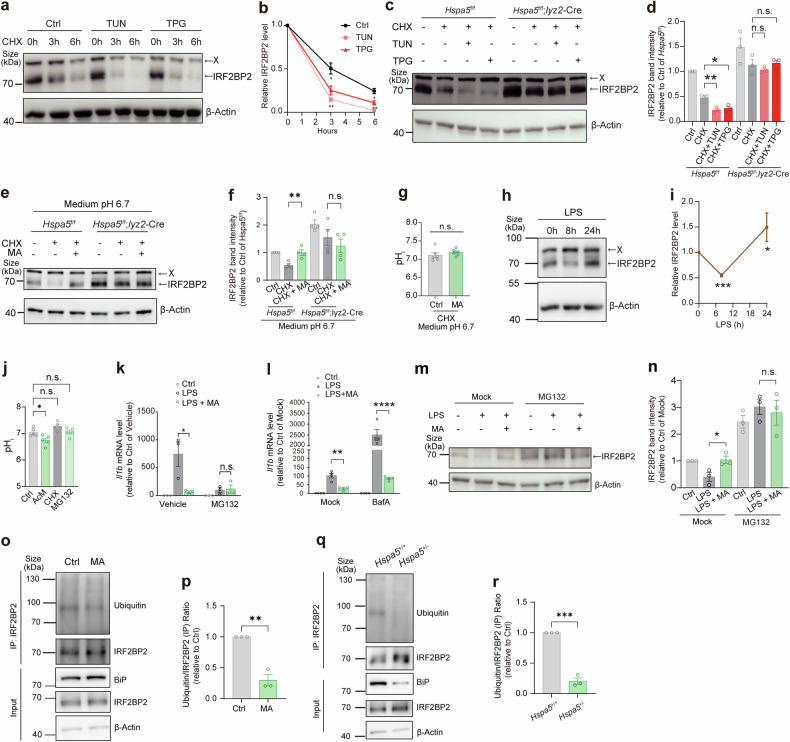
L-malate protects IRF2BP2 from BiP-driven protein degradation. (**a** and **b**) IRF2BP2 protein levels in CHX-treated (0 h, 3 h, and 6 h) BMDMs incubated with Tunicamycin (0.1 μM) or Thapsigargin (0.1 μM) added 16 h before CHX addition. (**c** and **d**) IRF2BP2 protein levels visualized (**c**) and quantified (**d**) by immunoblotting of BMDMs isolated from *Hspa5*^f/f^ or *Hspa5*^f/f^; *Lyz2*-Cre mice. These BMDMs were treated by CHX (3 h) and incubated with/without Tunicamycin (0.1 μM) or Thapsigargin (0.1 μM) added 16 h before CHX addition. (**e** and **f**) IRF2BP2 abundance was visualized by immunoblotting (**e**) and quantification (**f**) of IRF2BP2 in BMDMs from *Hspa5*^f/f^ or *Hspa5*^f/f^; *Lyz2*-Cre mice. These BMDMs under a controlled medium pH (6.7) were treated by CHX and incubated with or without L-malate (6 mM, 6 h). (**g**) pH_i_ of BMDMs (controlled medium pH 6.7) treated with CHX (2 h) and incubated with or without L-malate (6 mM, 6 h). (**h** and **i**) IRF2BP2 abundance was visualized by immunoblotting (**h**) and quantification (**i**) of IRF2BP2 in BMDMs treated by LPS for 0, 8 h and 24 h. n = 3. (**j**) pH_i_ of BMDMs treated with acidized medium (pH 6.7, 3 h), MG132 (5 μM, 0.5 h) or CHX (6 h). (**k**) *Il1b* mRNA levels in LPS-stimulated BMDMs treated with/without L-malate (6 mM) or MG132 (0.5 μM). In addition, MG132 (5 μM) was added in MG132-treated groups 0.5 h before LPS stimulation. (**l**) *Il1b* mRNA expressions in LPS-stimulated BMDMs treated with/without L-malate (6 mM, medium pH 6.7) and bafilomycin A (BafA) for 24 h. (**m** and **n**) IRF2BP2 protein levels in unstimulated and LPS-stimulated BMDMs treated with L-malate (6 mM, medium pH 6.7) and MG132 (0.5 μM) for 8 h. In addition, MG132 (5 μM) was added in MG132-treated groups 0.5 h before LPS stimulation. (**o** and **p**) IRF2BP2 ubiquitination shown by Western blot analysis of co-immunoprecipitation in MG132-pretreated (5 μM, 0.5 h) Raw264.7 cells treated with MG132 (0.5 μM) and L-malate (6 mM) in medium pH 6.7 for 3 h. (**q** and **r**) IRF2BP2 ubiquitination shown by Western blot analysis of co-immunoprecipitation in MG132-treated (5 μM, 0.5 h) *Hspa5*^+/+^ or *Hspa5*^+/−^ Raw264.7 cell line. The “X” means unknown proteins detected in immunoblotting visualizing IRF2BP2. n ≥ 3. Data are shown as mean ± SEM. *p < 0.05; **p < 0.01; ***p < 0.001; ****p < 0.0001 (unpaired Student’s *t*-test)

We subsequently aimed to analyze the mechanism underlying regulation of IRF2BP2 protein levels by L-malate-BiP axis. The impact of BiP and L-malate on the protein abundance rather than mRNA levels of IRF2BP2 prompt us to test whether L-malate-BiP axis regulates the stability of IRF2BP2 proteins. We used cycloheximide (CHX), a protein synthesis inhibitor, and found that BiP inducers reduced the stabilities of IRF2BP2 protein in CHX-treated BMDMs (Fig. 5a, b). Further, in a BiP-dependent way, IRF2BP2 proteins were destabilized by BiP inducers (Fig. 5c, d), and stabilized by L-malate (Fig. 5e, f) which did not reduce pH_e_ (controlled medium pH 6.7) or pH_i_ (Fig. 5g). Next, we inquired how L-malate-BiP axis regulates the stability of IRF2BP2 protein. BiP is regarded as a molecular platform located in the cytoplasm and nucleus that facilitates the degradation of its binding target and thus controls a specialized signaling pathway.^70–72^ The stability of IRF2BP2 is reportedly modulated by ubiquitin and proteasome-dependent degradation, which is regulated by its binding protein.^73^ Although decreased by LPS treatment for 8h^36^ (Fig. 4a, b, Fig. 5h, i), IRF2BP2 protein levels recovered from 8 h to 24 h of LPS stimulation (Fig. 5h, i), which could be explained by the LPS-induced inhibition of BiP-IRF2BP2 interaction in macrophages, while the downregulation of IRF2BP2 in the early stage of LPS treatment may be attributed to activation of the proteasome mediated by LPS-TLR4 signaling.^74,75^ Accordingly, we tested the role of ubiquitin-proteasome system in regulation of IRF2BP2 and IL-1β by L-malate-BiP axis. We used a proteasome inhibitor MG132 that were not detected to change pH_i_ of BMDMs (Fig. 5j), and found that MG132 abolished the effect of L-malate addition on LPS-induced expressions of *Il1b* (Fig. 5k), while the autophagosome–lysosome fusion inhibitor bafilomycin A did not affect the anti-inflammatory regulation of L-malate supplementation (Fig. 5l). Moreover, in MG132-treated pro-inflammatory macrophages, L-malate failed to up-regulate IRF2BP2 protein abundance (Fig. 5m, n). Consistently, through Co-IP assay in MG132-pretreated cells, we found reduced ubiquitylation of IRF2BP2 in L-malate-treated macrophages (Fig. 5o, p) and in the *Hspa5*^*+/−*^ Raw 264.7 cells (Fig. 5q, r). Collectively, these findings demonstrated that the degradation of IRF2BP2 are regulated by BiP and by L-malate in a BiP-dependent manner.

### BiP-IRF2BP2 signaling senses cytosolic protons

The structure-based pH-sensitivity of L-malate (Supplementary Fig. 4g), as well as three aspects of interesting observations prompted us to further analyze the correlation between pH_i_ and L-malate-triggered BiP-IRF2BP2 signaling. (1) The pH_i_ alkalizer predominantly affected the anti-inflammatory regulation of L-malate supplementation, and this effect could be reversed by the pH_i_ acidifier (Supplementary Fig. 4f). (2) Conversely, the promotive effect of pH_i_ alkalizer on *Il1b* expressions was blocked in the L-malate accumulation model (Supplementary Fig. 4j) mimicked by *Mdh2* deficiency which induced a greater increase in intracellular L-malate (Supplementary Fig. 4h) than that induced by 6 mM L-malate supplementation (Supplementary Fig. 5c, h) but did not affect the pH_i_ of LPS-stimulated macrophages (Supplementary Fig. 4i). (3) Although attributed to both L-malate and pH_i_, the anti-inflammatory effect of L-malate addition was almost completely blocked by BiP deficiency (Fig. 2h), by IRF2BP2 deficiency (Fig. 4p, q) and by MG132-induced stabilization of IRF2BP2 protein (Fig. 5k, m, n). Overall, these data suggested that effects of pH_i_ fluctuations on IL-1β productions could be, at least partially, attributed to a potential impact of pH on BiP-IRF2BP2 pathway.

**Fig. 6:**
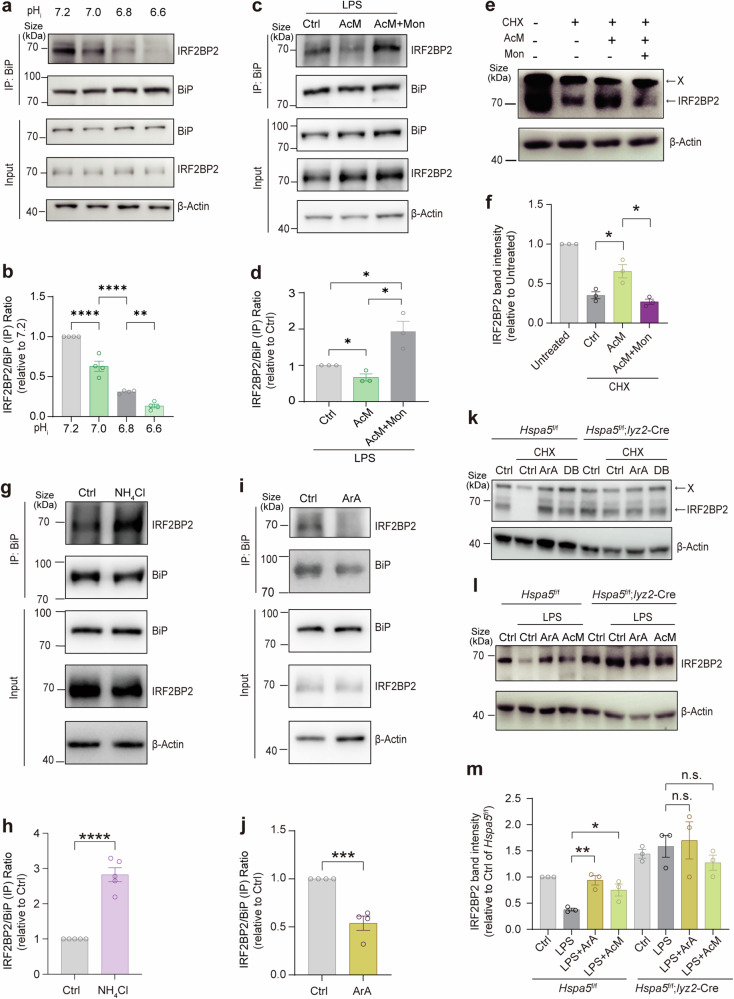
BiP-IRF2BP2 signaling senses intracellular protons independently of lysosomal pH. (**a** and **b**) Cellular BiP-IRF2BP2 interactions shown by Western blot analysis of co-immunoprecipitation in MG132-pretreated (5 μM, 2 h) peritoneal macrophages which were incubated with Nigericin (10 μM), Valinomycin (10 μM) and MG132 (5 μM) for 3 h in calibration buffer under different pH (7.2, 7.0, 6.8, 6.6). (**c** and **d**) Cellular BiP-IRF2BP2 interactions shown by Western blot analysis of co-immunoprecipitation in MG132-pretreated (5 μM, 0.5 h) Raw264.7 cells which were treated with acidized medium (AcM) (pH 6.7) or with AcM plus monensin (0.1 μM) in the presence of LPS and MG132 (0.5 μM) for 6 h. (**e** and **f**) IRF2BP2 protein levels visualized (**e**) and quantified (**f**) by Western blot in CHX-treated (6 h) BMDMs with or without acidized medium (AcM) (pH 6.7) and monensin. (**g** and **h**) Cellular BiP-IRF2BP2 interactions shown by Western blot analysis of co-immunoprecipitation in MG132-pretreated (5 μM, 0.5 h) Raw264.7 cells which were incubated by Ringer’s solution containing glucose (11 mM), MG132 (0.5 μM) with or without NH_4_Cl (30 mM) for 2 h. (**i** and **j**) Cellular BiP-IRF2BP2 interactions shown by Western blot analysis of co-immunoprecipitation in MG132-pretreated (5 μM, 0.5 h) Raw264.7 cells which were treated MG132 (0.5 μM) by with or without ArA (200 μM, neutralized pH) for 6 h. (**k**) IRF2BP2 protein abundance was visualized by immunoblotting in BMDMs isolated from *Hspa5*^f/f^ or *Hspa5*^f/f^; *Lyz2*-Cre mice. These CHX-treated BMDMs were treated with/without ArA or DB (9 h). (**l** and **m**) IRF2BP2 protein levels in BMDMs isolated from *Hspa5*^f/f^ or *Hspa5*^f/f^; *Lyz2*-Cre mice. These LPS-stimulated BMDMs were treated with/without AcM (pH 6.7) or ArA (8 h). n ≥ 3. Data are shown as mean ± SEM. *p < 0.05; **p < 0.01; ***p < 0.001; ****p < 0.0001 (unpaired Student’s *t*-test)

Next, we determined whether pH fluctuations affect BiP-IRF2BP2 signaling in cells independently of exogenous L-malate. We first calibrated pH_i_ with Nigericin and Valinomycin in the calibration buffer in accordance to the commercial kit. Strikingly, the BiP-IRF2BP2 interaction in macrophages was inhibited by intracellular acidification in a pH-dependent way (Fig. 6a, b). Subsequent Co-IP assays showed that the BiP-IRF2BP2 interaction was slightly inhibited by acidized medium (AcM) but enhanced by treatment of AcM with monensin in LPS-primed macrophages (Fig. 6c, d), which can be explained by the potential Monensin-driven secondary active transport of H^+^ relying on the inward Na^+^ gradient maintained by Na^+^/K^+^ ATPase.^76,77^ Further, acidized media alleviated the degradation of IRF2BP2, and monensin abolished this effect of acidized media (Fig. 6e, f). Also, another pH_i_ alkalizer, NH_4_Cl, promoted BiP-IRF2BP2 interaction (Fig. 6g, h) in macrophages. These data of pH_i_ modulators confirmed that the BiP-IRF2BP2 interaction/signaling senses cytosolic protons. Subsequently, we identified subcellular location of protons sensed by BiP-IRF2BP2 interaction/signaling relative to lysosomes using the activator of TMEM175 that trigger proton release from lysosomes including arachidonate (ArA) and DCPIB (DB) to upregulate cytosolic protons.^78^ ArA reduced the cellular BiP-IRF2BP2 interaction (Fig. 6i, j). Both ArA and DB protected IRF2BP2 from degradation in a BiP-dependent manner (Fig. 6k). Furthermore, both ArA and AcM increased the protein abundance of IRF2BP2 in LPS-treated BMDMs, and this effect was not observed in BiP-knockout macrophages (Fig. 6l, m). Overall, these data demonstrated that, independently of lysosomal acidification, cytosolic protons inhibit BiP-IRF2BP2 interaction in cells and induce IRF2BP2 accumulation through BiP.

### pH reduction facilitates L-malate to bind BiP and to inhibit BiP-IRF2BP2 interaction in a carboxyl-dependent manner

L-malate is an endogenous carboxylate whose monoprotonation is sensitive to weakly acidic pH due to its high pKa2 (Supplementary Fig. 4g). In fact, some carboxylate metabolites reportedly play key roles in the impact of pH_i_ on enzyme functions and cell‒cell communications, mainly through a high monoprotonation sensitivities of dicarboxylates.^15,16^ Metabolites that can be sensitively regulated are considered to act as an intrinsic signaling modality initiating physiological adaptation.^15,17,18^ Consequently, we hypothesized that the protonated L-malate are, at least one of, the signaling intersection of cytosolic protons and L-malate to control BiP-IRF2BP2 signaling.

**Fig. 7:**
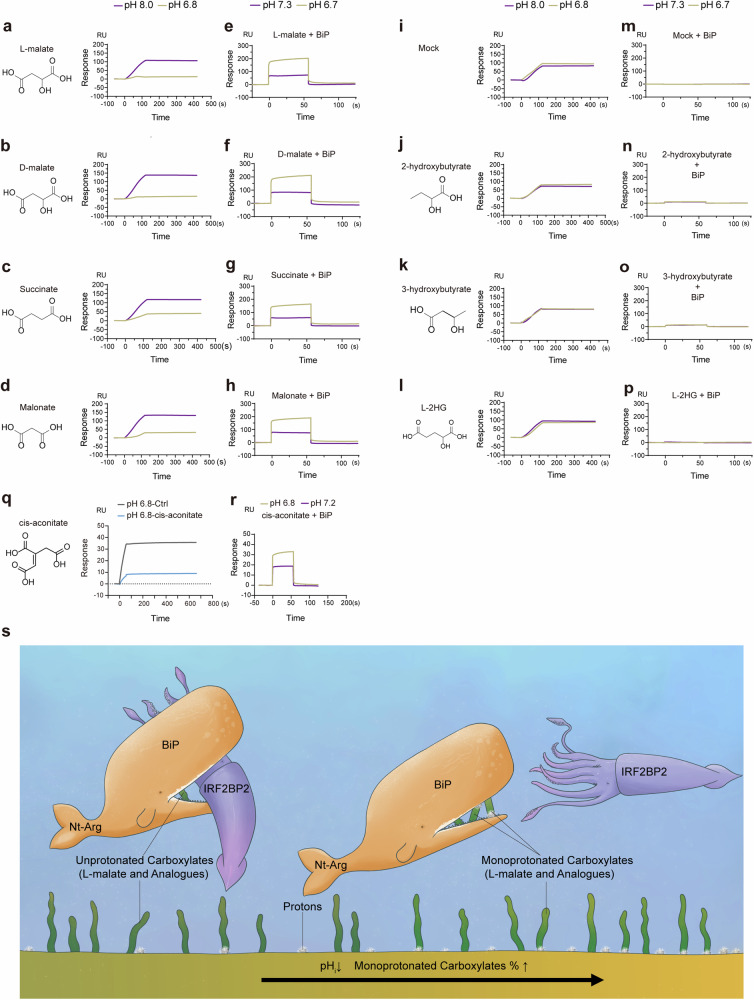
pH reduction facilitates L-malate and analogues to bind BiP and to inhibit BiP-IRF2BP2 interaction. (**a**-**d**, **i**-**l**) BIAcore diagram of BiP (chip-coupled) and IRF2BP2 (25 nM) with or without different small molecules at concentration of 0.2 mM in different pH (8.0 indicated in purple and 6.8 indicated in brown). (**e**-**h**, **m**-**p**) BIAcore diagram of different small molecules at concentration of 1 mM with BiP protein (chip-coupled) in different pH (7.3 indicated in purple, 6.7 indicated in brown). (**q**) BIAcore diagram of BiP (chip-coupled) and IRF2BP2 (25 nM) with or without cis-aconitate (500 μM) in pH 6.8. (**r**) BIAcore diagram of cis-aconitate (1 mM) BiP protein (chip-coupled) in different pH (7.2 indicated in purple, 6.8 indicated in brown). (**s**) A conceptual graph illustrating the role of L-malate and analogues in pH sensing of BiP-IRF2BP2 interaction. *n* = 3. See also Supplementary Fig. 10

To verify our hypothesis, we examined effects of pH on L-malate-induced disruption of BiP-IRF2BP2 binding and on L-malate-BiP interaction. In SPR, the presence of L-malate permitted the inhibitory effect of pH reduction on BiP-IRF2BP2 binding while the acidic pH facilitated L-malate to restrain BiP-IRF2BP2 interaction (Fig. 7a). Further, acidification enhanced L-malate-BiP bindings in SPR (Fig. 7e, Supplementary Fig. 10a). Additionally, in SPR assays for L-malate and BiP domains, we found that reduced pH promoted bindings of L-malate with either the N-terminal domain (Supplementary Fig. 10b) or the C-terminal domain (Supplementary Fig. 10f) instead of other domains (Supplementary Fig. 10c–e). In sum, these data supported our hypothesis that protons and L-malate synergistically regulate BiP-IRF2BP2 interaction.

We next analyzed the potential biochemical basis in BiP underlying the pH sensitivities of L-malate-BiP-IRF2BP2 axis which consists of L-malate-BiP binding and L-malate-induced disruption of BiP-IRF2BP2 interaction. There are two well-established biochemical mechanisms of proton sensing by proteins: the protonation in histidine,^79,80^ and the protonation-mediated formation of carboxyl-carboxyl pairs.^81,82^ However, L-malate is a dicarboxylate that lacks histidine group. And in the sequence of BiP, both two domains binding L-malate in a pH sensitive way, the N-terminal domain (aa 1-125) lacking histidine residue and the C-terminal domain (aa 500-654) containing only one histidine residue, are both rich in amino acids containing the carboxyl side chain (Supplementary Fig. 10g), which is structurally possible to form the carboxyl-carboxyl pairs with either or both two carboxyls in L-malate. Moreover, the monocarboxylic pKa (monoprotonation-related pKa and pKa2 of dicarboxylate) of L-malate is higher and closer to 7.0 than the pka of carboxyl side chains in amino acids (Supplementary Fig. 4g) and can be further increased by the near-neighbour carboxyl.^38^ Accordingly, we proposed that the potential carboxyl-carboxyl pair formed between L-malate and BiP is more likely to explain the pH sensitivity of interactions of BiP with either L-malate or IRF2BP2.

We also tested the role of L-malate’s carboxyls in the structure-activity relationship of the effect on BiP-IRF2BP2 interaction and BiP binding of L-malate using carboxylate analogues of L-malate. (1) D-malate, the enantiomer of L-malate, as well as succinate which only lacks the hydroxyl, was detected to disrupt BiP-IRF2BP2 interaction (Fig. 7b, c) and to bind BiP (Fig. 7f, g) in a pH-sensitive manner. (2) Malonate, the minimum C3 dicarboxylate (which differs from malate with its shorter carbon chain and lack of hydroxyl) showed a pH-sensitive disruption of BiP-IRF2BP2 interaction (Fig. 7d) and acidity-enhanced affinity with BiP (Fig. 7h). (3) Vehicle control, 2-hydroxybutyrate and 3-hydroxybutyrate which only have one carboxyl replaced with methyl compared to L-malate, did not show a pH-sensitive disrupt BiP-IRF2BP2 interaction (Fig. 7i-k) or affinity for BiP (Fig. 7m–o). (4) L-2-hydroxyglutarate (L-2HG), a dicarboxylate that only differs from malate with a longer carbon chain (L-malate C4, L-2HG C5), was not detected to disrupt BiP-IRF2BP2 interaction (Fig. 7l) or to bind BiP (Fig. 7p). Furthermore, we also inquired whether a tricarboxylate analogue of L-malate can bind BiP and disrupt BiP-IRF2BP2. We tested cis-aconitate, a tricarboxylate with high monoprotonation-related pKa (~6.2, pKa3 of tricarboxylate), whose supplementation also potently inhibited the expressions of pro-inflammatory genes (Fig. 1a, and Supplementary Fig. 1j, k) in our metabolites screening assay in LPS-stimulated macrophages, and we detected the cis-aconitate-induced disruption of BiP-IRF2BP2 binding (Fig. 7q), as well as the cis-aconitate-BiP interaction which was enhanced by pH reduction (Fig. 7r). Collectively, these data demonstrated that besides L-malate, its certain carboxylate analogues can also bind BiP and disrupt BiP-IRF2BP2 interaction in a pH-sensitive way, and two carboxyls connected by a carbon chain with proper length (shorter than or equal to 4) in BiP-binding carboxylates is the critical structure of both carboxylate-BiP binding and acidity-facilitated inhibitory effects of BiP-binding carboxylate on BiP-IRF2BP2 binding, and in other words, a carboxylate-based structure-activity relationship is shared by carboxylate-BiP binding and pH-carboxylate-BiP-IRF2BP2 axis. Accordingly, we proposed that acidity-facilitated disruptions of BiP-IRF2BP2 interaction induced by BiP-binding carboxylates, as well as pH-sensitive bindings of BiP with BiP-binding carboxylate metabolites such as L-malate, succinate and cis-aconitate can, at least partially, explain the proton sensing by the BiP-IRF2BP2 interaction in cells, while L-malate’s high pKa, cytosolic levels, affinity to BiP, and its potential lack of ability to trigger pro-inflammatory signals like succinate,^19^ could synthetically explain its relative dominance among these BiP-binding carboxylates in anti-inflammatory regulation in cells.

Next, we simulated the binding model of BiP with malate via molecular docking. Considering the predominance of unprotonated malate in proportion among all its protonation states within the physiological pH range, and the pH-insensitive affinity of BiP fragments with malate in pH 7.4 ~ 8.0 (Supplementary Fig. 10f), we first modeled the binding pocket that binds the unprotonated malate within the N/C-terminal domains of BiP showing that two carboxyls in malate interact with the -NH_3_^+^ side chain of amino acids in N- and C- terminal domains, respectively (Supplementary Fig. 10i). Moreover, when we increased the concentration of BiP-binding carboxylates including L-malate, pH reduction failed to further enhance carboxylate-binding (Supplementary Fig. 10h), which indicated that pH reduction only facilitates carboxylate to bind BiP via upregulating their binding ratios. Accordingly, we simulated the binding pocket of BiP with monoprotonated malate, in which the one carboxyl with lower pKa in malate interacts with -NH_3_^+^ of amino acid and a carboxyl-carboxyl pair is formed between the other carboxyl with higher pKa in malate and -COO^–^ side chain of C-terminal amino acid (Supplementary Fig. 10j). Notably, these two present pockets without the involvement of the -COO^–^ side chain of N-terminal amino acid (Supplementary Fig. 10i, j) were not sufficient to explain the independent pH sensitivity of the binding between malate and the N-terminal fragment (aa 25-125) of BiP (Supplementary Fig. 10b). Thus, we simulated the third binding pocket with flexible docking, which includes the interaction of malate with -NH_3_^+^ side chains of C-terminal amino acids and a carboxyl-carboxyl pair between malate and N-terminal amino acid (Supplementary Fig. 10j). Overall, we mimicked a button-like binding model for pH-sensitive malate-BiP interactions (Fig. 7s), and this model, together with the data showing the structure-activity relationship of malate (Fig. 7a–r), supported our mechanistic speculation that the co-sensing of pH by BiP and carboxylates requires the formation of carboxyl-carboxyl pair, a “pKa-increasing” chemical basis^38^ for an adequate proton-sensitivity under pH around 7.0.

### pH fluctuations regulate IL-1β production via BiP-IRF2BP2 axis in vitro

**Fig. 8:**
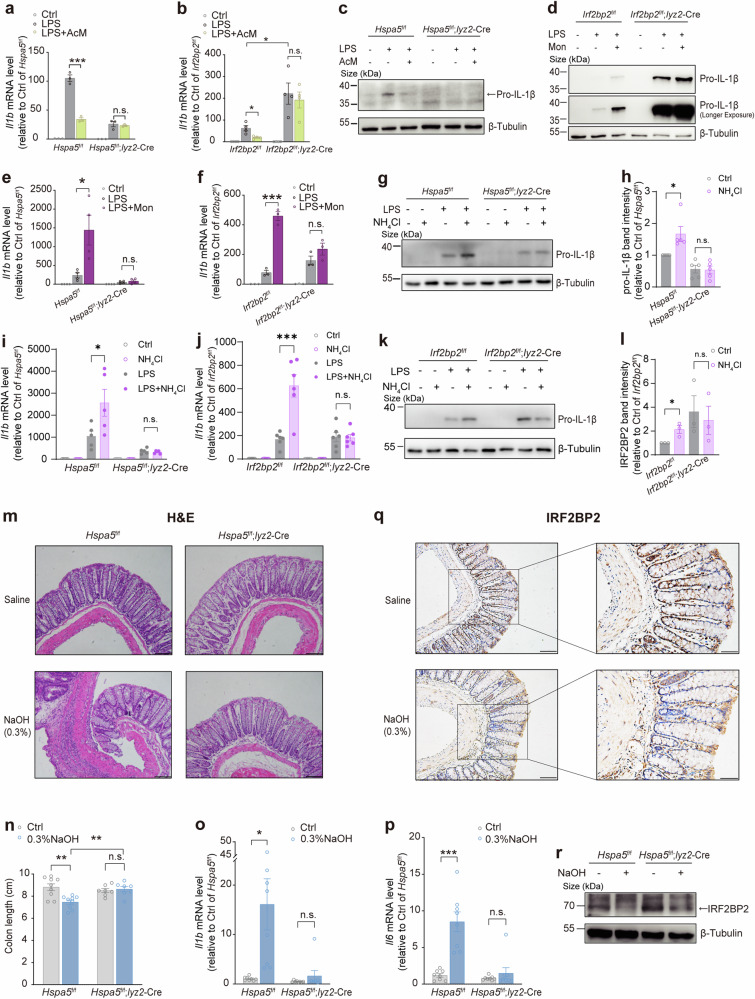
BiP-IRF2BP2 signaling links pH fluctuation with inflammation. (**a**, **c**) *Il1b* mRNA levels (**a**) and pro-IL-1β protein abundance (**c**) in BMDMs isolated from *Hspa5*^f/f^ or *Hspa5*^f/f^; *Lyz2*-Cre mice treated with or without acidized medium (AcM) (pH 6.7), under stimulation of LPS for 24 h. (**b**) *Il1b* mRNA levels by qPCR in BMDMs isolated from *Irf2bp2*^f/f^ or *Irf2bp2*^f/f^; *Lyz2*-Cre mice treated with or without acidized medium (AcM) (pH 6.7), under stimulation of LPS for 24 h. (**d**, **f**) Protein abundance of pro-IL-1β (**d**) and mRNA levels of *Il1b* (**f**) in BMDMs isolated from *Irf2bp2*^f/f^ or *Irf2bp2*^f/f^; *Lyz2*-Cre mice treated with or without Monensin (1 μM), under stimulation of LPS for 24 h. (**e**) *Il1b* expressions in BMDMs isolated from *Hspa5*^f/f^ or *Hspa5*^f/f^; *Lyz2*-Cre mice treated with or without monensin (1 μM), under stimulation of LPS for 24 h. (**g**, **h**) pro-IL-1β protein levels in BMDMs isolated from *Hspa5*^f/f^ or *Hspa5*^f/f^; *Lyz2*-Cre mice treated with or without NH_4_Cl (15 mM), under stimulation of LPS for 16 h. (**i**) *Il1b* mRNA levels in BMDMs isolated from *Hspa5*^f/f^ or *Hspa5*^f/f^; *Lyz2*-Cre mice treated with or without NH_4_Cl (15 mM), under stimulation of LPS for 8 h. (**j**) *Il1b* mRNA levels in BMDMs isolated from *Irf2bp2*^f/f^ or *Irf2bp2*^f/f^; *Lyz2*-Cre mice treated with or without NH_4_Cl (15 mM), under stimulation of LPS for 8 h. (**k**, **l**) pro-IL-1β protein levels in BMDMs isolated from *Irf2bp2*^f/f^ or *Irf2bp2*^f/f^; *Lyz2*-Cre mice treated with or without NH_4_Cl (15 mM), under stimulation of LPS for 16 h. (**m**) Representative H&E-stained sections of colons from *Hspa5*^f/f^, *Hspa5*^f/f^; *Lyz2*-Cre mice sampled on day 6 after the saline or NaOH exposure. Scale bar 100 μm. (**n**) Length of colons from *Hspa5*^f/f^ or *Hspa5*^f/f^; *Lyz2*-Cre mice at day 6 of NaOH-induced colitis model (n = 7-9 per group). (**o**, **p**) mRNA levels of *Il1b* (**o**) and *Il6* (**p**) in colons collected at day 3 after the saline or NaOH exposure, analyzed by qPCR. (**q**) Immunoreactive staining (brown) for IRF2BP2 [scale bar, 100 (left) or 50 (right) μm]. (**r**) Western blot of IRF2BP2 in colons of mixed sample from *Hspa5*^f/f^ or *Hspa5*^f/f^; *Lyz2*-Cre mice at day 3 of NaOH-induced colitis model (n = 8 per group). The “X” means unknown proteins detected in immunoblotting visualizing IRF2BP2. n ≥ 3. Data are shown as mean ± SEM. *p < 0.05; **p < 0.01; ***p < 0.001 (unpaired Student’s *t*-test)

We then verified whether BiP-IRF2BP2 signaling mediates the effect of pH fluctuations on inflammatory response. Importantly, the downregulation of IL-1β levels induced by AcM (Fig. 8a–c) and the upregulation of IL-1β production induced by monensin (Fig. 8d–f) were blocked by the deficiency of BiP (Fig. 8a, c, e) and IRF2BP2 (Fig. 8b, d, f) in LPS-primed macrophages. Moreover, we also tested another pH_i_ alkalizer NH_4_Cl, and we found its moderate but significant upregulation of IL-1β levels in LPS-stimulated macrophages, while not in that with BiP deficiency (Fig. 8g–i) or IRF2BP2 deficiency (Fig. 8j–l). Taken together, these data demonstrated that BiP-IRF2BP2 signaling mediates the impact of pH fluctuation on IL-1β production in pro-inflammatory macrophages.

### BiP-IRF2BP2 signaling links pH fluctuations with inflammation in vivo

Further, we examined the involvement of the BiP-IRF2BP2 axis in the relationship between pH fluctuation and inflammation in vivo. Macrophages in the healthy intestine usually respond to the gut microbiota without provoking an inflammatory response, and a breakdown in this tolerance leads to inflammatory bowel disease (IBD).^83^ It has been reported that acid-suppressive medications are clinically relevant to IBD,^84,85^ and combined analyses of three human cohorts showed that regular proton pump inhibitor (PPI) users have an increased risk of IBD compared with nonusers.^27^ Furthermore, an experimental rat model of Crohn’s colitis induced by NaOH has been previously established.^86^ Additionally, a recent study revealed that perturbation of IRF2BP2 could be a strategy to unleash cell-intrinsic inflammation.^37^ These reports, along with our present findings, prompted us to test the possibility that disturbances of local physiological pH and BiP-IRF2BP2 signaling can unleash an inflammatory response in the intestine. Therefore, considering the possible off-target effect of PPI, we chose NaOH to mimic loss of proton in intestinal environment basically according to the reported NaOH-induced colitis model,^86^ but reduced the NaOH concentration from 6.25% to 0.3%, 75 μmol/g, which is lower than the concentration of fecal Na^+^ excretion in mice around 200 μmol/g,^87^ excluding a major effect on Na^+^. Interestingly, rectal administration of 0.3% NaOH solution induced intestinal inflammation (Fig. 8m), colon shortening (Fig. 8n), and upregulation of inflammatory cytokines IL-1β (Fig. 8o) and IL-6 (Fig. 8p). Importantly, myeloid deficiency of BiP ablated the NaOH-induced phenotypes of colitis mentioned above (Fig. 8m-p). Consistently, the protein abundance of IRF2BP2 were decreased in mice treated with NaOH in IHC (Fig. 8q) and western blot (Fig. 8r), whereas the downregulation of IRF2BP2 was also detected in *Hspa5*^f/f^; *lyz2*-Cre mice (Fig. 8r), which may be attributed to the reaction of non-myeloid cells to NaOH. Notably, in immunohistochemical staining of the colon mucosa of the control group, the protein levels of IRF2BP2 appeared lower near the colonic epithelium, a more alkaline environment (Fig. 8q), and this physiological distribution of IRF2BP2 was in line with the pH-sensing feature of IRF2BP2 protein stability. Collectively, these results demonstrated a proton-dependent maintenance of intestinal immunological homeostasis in which BiP-IRF2BP2 signaling plays a key role.

## Discussion

The endogenous metabolite is an ideal type of lead compounds in drug discoveries as well as a potential signal transducer. Here, we unexpectedly reveal that metabolic and environmental cues merge into a previously unrecognized carboxylate/proton dual sensing signaling pathway. Unbiased progressive screenings and experimental verification with multiple approaches identified L-malate’s profound anti-inflammatory activity and, after excluding the involvement of glycolysis, fumarate and canonical UPR pathways, uncovered its underlying mechanism, a BiP-IRF2BP2 pathway involving L-malate-BiP binding, L-malate-restrained BiP-IRF2BP2 binding and BiP-driven degradation of IRF2BP2. The pH reduction, which promotes carboxyl monoprotonation of carboxylate, facilitates L-malate and analogue to bind BiP and to disrupt BiP-IRF2BP2 binding in a carboxyl-dependent way. Similarly to L-malate, acidifications inhibit BiP-IRF2BP2 interaction, protect anti-inflammatory IRF2BP2 protein from degradation driven by BiP, and impairs LPS-induced IL-1β production through BiP-IRF2BP2 pathway in macrophages. Conversely, pH_i_ alkalizers enhance BiP-IRF2BP2 interactions and promote IL-1β production via BiP-IRF2BP2 pathway in macrophages. Furthermore, in mouse colons, NaOH that reduces protein levels of IRF2BP2, induces a myeloid-BiP-dependent colitis. These findings uncover the non-metabolic immunoregulatory/biochemical effect of L-malate, which provides potential therapeutic agent/targets to treat the inflammatory diseases, and evidence the role of L-malate-initiated intracellular protein signaling pathway in responses to local environmental pH.

During activations of proinflammatory macrophage, distinct nonmetabolic pathways that positively or negatively regulate inflammatory gene expressions can be triggered by gradual accumulations of the intermediate and the derived metabolite in TCA cycle such as succinate that profoundly augments IL-1β productions at the early stage (3 h) of LPS stimulations in macrophages.^3,5,19^ Along with the LPS-induced accumulations of L-malate and carboxylate analogues^19,21^ (Supplementary Fig. 5i), the BiP-IRF2BP2 interaction in macrophages is reduced during the LPS stimulation (Fig. 3j, k). The abundance of IRF2BP2 protein, the anti-inflammatory downstream effector of BiP-IRF2BP2 signaling, are downregulated in the early stage (8 h) and are recovered in the late stage (24 h) of LPS-induced macrophages activation (Fig. 5h, i), which can be explained by that the proteasome activation mediated by LPS-TLR4 signaling^74,75^ counterbalances the tendency of BiP-IRF2BP2 signaling to stabilize IRF2BP2 protein in early stage of TLR4 activation and thereby delayed the activation and anti-inflammatory regulation of the BiP-IRF2BP2 pathway. During later stage of LPS stimulation, levels of intracellular L-malate whose anti-inflammatory effect was structure-dependent but metabolism-independent (Supplementary Fig. 6a–i), were further increased (Supplementary Fig. 5i). And consistently, IRF2BP2 deficiency drastically upregulated the levels of IL-1β in 24 h LPS stimulation (Fig. 4p, q and Fig. 8b, d), but during 16 h LPS stimulation, only a moderate increase the levels of IL-1β were detected (Fig. 4m–o, Fig. 8k, l). We consequently proposed that during proinflammatory polarizations of macrophages, the L-malate-inducible BiP-IRF2BP2 signaling acts as a negative feedback mechanism whose delayed activations contributes to the balance between prompt initiation and timely termination of the inflammatory response. Notably, although the inhibition of IL-1β by IRF2BP2 was found in multiple studies,^36,37,88,89^ the mechanism underlying the regulation of IL-1β by IRF2BP2 remains unclear. IRF2BP2 positively regulates the promoter activity of anti-inflammatory Krüppel-like factor 2 (KLF2) and mediates myocyte enhancer factor 2 (MEF2)-dependent KLF2 expressions.^36^ Moreover, the knockdown of KLF2 has been reported to partially reverse the reduction in *IL1b* mRNA levels induced by the overexpressions of IRF2BP2.^88^ On the other hand, IRF2BP2 was first discovered to be a corepressor of IRF2.^90^ It has been reported that IRF2^−/−^ mice are highly resistant to LPS-induced lethality and that serum protein levels of *Il1b* are downregulated in IRF2^−/−^ mice after LPS challenge.^91^ Furthermore, *Il1b* chromatin is bound by IRF2BP2,^37^ which suggests a potential direct mechanism of IRF2BP2-induced *Il1b* inhibition at the transcriptional level. Therefore, we hypothesize that IRF2BP2 inhibits *Il1b* expressions partly through the MEF2-KLF2 axis and that IRF2BP2 binds to *Il1b* chromatin in a IRF2-dependent or IRF2-independent manner, thereby inhibiting IL-1β transcription. Taken together with the related studies, the regulation of IL-1β by IRF2BP2 may not be explained by a single mechanism, while the potential effect induced by the binding of IRF2BP2 and *Il1b* chromatin on *Il1b* expressions as well as the mechanistic link of KLF2 and IRF2 with IL-1β levels need further investigation, which are critical for the application of malate and analogues that triggers BiP-IRF2BP2 signaling in the clinical anti-inflammatory therapy.

In sites of immune activity, the pH_e_ of local microenvironments can be reduced into values as low as 6.6 in joint fluids of patients with the inflammatory joint disease^31^ and below 6.0 in the peritoneal fluid of infected patients.^92^ Acidic pH_e_ can reduce the pH_i_ of macrophages (Fig. 5j). Although macrophages tend to recover pH_i_ in the acidic inflammatory milieu, this pH_i_ recovery can be impaired by TLR4 activation.^34^ There is also report demonstrating the acidification of pH_i_ in human phagocytes induced by high-density bacterial exposure.^93^ The ability of phagocytes to drive innate immune response can be impaired by acidifications of their microenvironments^40,94^ and cytoplasm.^95–97^ CO_2_-induced intracellular acidification is specifically regarded as responsible for the reduced inflammation observed during CO_2_ pneumoperitoneum in laparoscopic surgery,^29–31^ and clinical acidemias are accompanied by immunodeficiency, including a diminution of the inflammatory response.^30,97^ Consistently, weaker alkalinity is related to the anti-inflammatory effect of nanoflakes on magnesium implants.^28^ Additionally, clinical acid-suppressive medications are relevant to inflammatory bowel diseases,^84,85^ and the personal use of proton pump inhibitors is associated with an increased risk of IBD and its subtypes.^27^ In monocytes, the proinflammatory effects of intracellular alkalizers have been revealed.^35^ Controversially, cytosolic acidification is also considered as an early transductory signal driving the chemotaxis initiation of human neutrophil.^98^ Also, it has been reported that the inflammasome activation and secretion of the inflammatory cytokine are promoted by acidic pH_e_ in early stage (3-6 h) of LPS stimulation in macrophages.^99,100^ Taking this controversy into account in our present study, we propose that the reduction in pH facilitates the rapid and potent initiation of innate immune response and then limits its duration, and this pH reduction thus acts as a fundamental physiological factor contributing to a successful immune response, which requires to be strong but often short-lived to ensure elimination of the pathogen followed by termination of the response, and ultimately, to the organism’s survival during evolutionary adaptation to environmental stressors.^10^

Markedly, these main components of this pH-sensing signaling pathway including some of BiP-binding carboxylates (such as L-malate and succinate), BiP and IRF2BP2, are ubiquitously expressed in various organisms ranging from simple to complex animals,^101,102^ which increases the possibility that the carboxylate-BiP-IRF2BP2 pathway plays a broader role in signaling transduction of pH fluctuations. Conceivably, the pH-sensing carboxylate-BiP-IRF2BP2 axis can be subjected to a variety of targeted manipulations to meet the requirement of therapy against autoimmune diseases, infections, cancers,^37^ and other health issues and to compensate for the pH-related inflammation-provoking side effects of interventions such as surgical replacements^28,103^ and acid-suppressive medications.^27^ Notably, IRF2BP2 is reported to bind lncRNA CHROMR and mediated its regulation of antiviral gene program in a recent study that also identified a critical role for CHROMR in controlling the restriction of influenza A virus replication in macrophages.^104^ Additionally, influenza A infection is reported to raise pH_i._^105^ Therefore, an interesting mechanistic insight into viral disruption of cellular innate immune response could be provided by investigating whether and how pH_i_-sensitive BiP interacts with CHROMR-IRF2BP2 axis. Besides processes of the innate immunity, the change in pH_i_ has been found in many other conditions, such as a rapid decrease in pH_i_ of rat aortic endothelial cells caused by shear stress forces,^106^ hypoxia-induced intracellular acidification as well as the increased pH_i_ induced by a decrease of temperatures from 35^°^C to 22^°^C in isolated sheep heart Purkinje fibres,^107^ the hyperoxia-induced intracellular acidification in neonatal mouse lung fibroblasts,^108^ and the glucose signaling through modulation of cytosolic pH in yeast cells.^109,110^ Likewise, IRF2BP2 not only reportedly plays a role in immune responses, but also gets upregulated in other physiological/pathological processes including exercise and ischemia^36,73,89,102^ characterized by a decrease in pH_e_ and pH_i_. Therefore, it is tempting to, at least partially, attribute elevated IRF2BP2 protein levels and its beneficial effect to the BiP-IRF2BP2 interaction inhibited by intracellular acidification during exercise and ischemia.^73,102^ Overall, IRF2BP2, a transcription co-factor^111^ known to play a role in inflammation, cholesterol handling,^36^ PD-L1 signaling,^63,112^ carcinogenesis,^113^ apoptosis,^114^ erythropoiesis,^101^ pathological cardiac hypertrophy,^115^ and other processes, may act as an “acidity-inducible factor” (ACIF) that mediates the biological effect of pH fluctuations during multiple physiologic/pathophysiologic processes, such as tumor immunity,^63,112^ extracellular chemical signals,^116^ nutrient limitation,^117^ ischemia,^118^ and other cell stresses.^119^ These potential roles of IRF2BP2 involved in the “acid signal” remains to be further investigated.

BiP is a marker and sensor of ERS, which can be induced by a variety of inflammation-provoking pathological conditions, including infection, hypoxia, oxidative injury, a high-fat diet, and hyperthermia.^120^ ERS and the UPR are involved in the control of immune responses.^20,121^ ERS is also widely reported to induce or promote productions of inflammatory mediators such as IL-6, TNF-α, and IL-23, through three well-characterized UPR branches.^20,122–124^ However, the immunoregulatory mechanism of BiP, which are independent of canonical UPR signaling, as well as the transcriptional modification of IL-1β by ERS remain poorly understood. These data in this study suggest that the upregulation of *Il1b* expressions in proinflammatory macrophages by an ER stressor is mainly mediated by the BiP-IRF2BP2 axis, which differs from known immunoregulatory pathways of the UPR. To note, ERS can promote BiP’s retrotranslocation from ER to cytosol independently of canonical UPR pathways.^65^ Future investigation, especially on the BiP-related signaling outside ER, to broaden the understanding of how ERS affects the innate immune response is interesting. As an increase in pH_i_ can facilitate BiP to bind and destabilize of the anti-inflammatory IRF2BP2 (Fig. 6a–f), additional attention should be paid to effects of ERS on the BiP-IRF2BP2 axis in tissue inflammation under nonacidic conditions or with alkaline treatment, such as microenvironments in intestine or tissues receiving surgical replacements with metal materials. In cells under ERS, a reported pH-sensing metabolic mechanism could also play a role in proton sensing of BiP. It could be expected that the acid-enhanced L-2HG production that stabilizes HIF1α^16^ and thereby reduces ERS,^125–127^ which is reported to upregulate cytosolic BiP in a UPR-independent manner,^65^ subsequently decreases levels of cytosolic BiP and BiP-bound IRF2BP2, and thereby stabilizes IRF2BP2 protein. Future investigations on the role of pH-sensitive L-2HG metabolism in the BiP-mediated proton sensing of cells under high-level ERS is interesting. Given the critical role of BiP in various biological activities, especially in the ER,^58,128–133^ cytosol^64,65^ and cell membrane,^134,135^ the interesting follow-up study regarding the role of BiP in cellular response to pH fluctuations may not be limited to their effects on the BiP-IRF2BP2 interaction. The physiological and physiopathological significances of the potential ERS/proton-sensing interactomes of BiP is nonnegligible. Also, considering the key role of BiP-binding ATP in biological functions of BiP,^58,128–130,132^ the potential role of ATP in BiP-mediated signalings in response to pH fluctuations is also interesting to be further investigated.

The data in this study indicates a critical role of the BiP-carboxylate binding in the pH sensitivity of the BiP-related molecular event. Similarly to BiP, some BiP-binding carboxylates such as L-malate and succinate locates in both intracellular spaces and certain extracellular spaces such as that of colon tissues in a high concentration^136–138^ which is close to the levels of BiP-binding carboxylates disrupting BiP-IRF2BP2 binding in SPR (Fig. 7a–d). Further, the data revealing that identical structure-activity relationships of pH-sensitive carboxylate-BiP binding and pH-carboxylate-BiP-IRF2BP2 axis were found as well as the dependence of BiP-binding carboxylates on the carboxyl (Fig. 7a–w), suggested that the carboxyl-carboxyl pair formed between BiP and BiP-binding carboxylate plays an essential role in pH-sensitivity of carboxylate-BiP-IRF2BP2 axis. Accordingly, the monoprotonation-related pKa of carboxyl in BiP-binding carboxylate (taking succinate as an example, 5.69 in water) can be the main chemical basis of the proton sensing by the carboxylate-BiP binding and BiP-mediated signaling, but it seems insufficient to explain the significant sensitivity at pH around 7.0 (Fig. 6a, b). However, it has been reported that the imposition of a restricting, near-neighbour arrangement for two carboxyl groups^38^ can lead to a pKa shift, which can be explained by the electronic polarization.^139^ For instance, the positioning of two carboxyl groups in different amino acid close in space, is considered to increase the pKa of one carboxyl group with 2–3 units,^38,81^ as well as the difference in pKa (4.4 and 6.1) between cis-trans dicarboxylate isomers which differ from each other mainly by the distance of two carboxyls.^38^ As for the carboxylate-BiP binding, when the carboxyl with the highest pKa in BiP-binding carboxylates approaches the carboxyl side chain in the amino acids of BiP, monoprotonation-related pKa of BiP-binding carboxylates can be increased to a value closer to 7.0. Furthermore, besides L-malate and succinate, the cis-aconitate, a tricarboxylate metabolite with potent inhibitory effects on expressions of pro-inflammatory cytokines (Fig. 1a, Supplementary Fig. 1j, k) and a higher monoprotonation-related pKa (~6.2, pKa3 of tricarboxylate, Supplementary Fig. 4g), suppressed BiP-IRF2BP2 binding (Fig. 7q) and binds to BiP in a pH sensitive way (Fig. 7r). Therefore, we propose that the carboxyl-carboxyl pair inducing pKa shift is one of a biochemical basis for the physiological significance of proton sensing by carboxylate-BiP binding and the diversity in pKa of BiP-binding carboxylates contributes to a wide sensitive range and the robustness of proton sensing by the carboxylate-BiP binding. In this study, we found that L-malate enters cells through MCT1 (Supplementary Fig. 5a), a symporter of carboxylate and proton, which can theoretically lead to a drop in pH_i_ during its transport of extracellular added L-malate. Intriguingly, under the pH_e_-controlled condition, extracellularly added L-malate did not decrease the pH_i_ (Figs. 2m, 3l, 5g). An exciting explanation about these data is that the BiP-binding carboxylate drives a tendency of intracellular alkalization that counterbalances the effect on pH_i_ of MCT1-mediated proton symport. The carboxylate metabolite in TCA cycle can accumulate during the condition that produces intracellular acid load, such as the metabolic shift into glycolysis.^19,21^ Subsequently, we proposed that, in order to stringently maintain pH_i_ in a distinct range,^140^ cells may require a feedforward regulation mediated by BiP-binding carboxylates in addition to the negative feedback triggered by pH fluctuations. Therefore, it is tempting to investigate the potential feedback and feedforward pH_i_ regulations that is initiated by carboxylate metabolites and/or protons. In summary, our findings propose a general model of how a metabolite-mediated pH-sensing mechanism drives cell autonomous regulation via non-metabolic function.

This study examined the anti-inflammatory effect and the corresponding downstream molecular pathway of L-malate mainly via the addition of L-malate. Also, loss of function of pH_i_ modulator induced by BiP-binding carboxylate intervention was only verified through L-malate’s endogenous accumulation model. The lack of an animal/cell model with the depletion of L-malate and its endogenous carboxylate analogues led to difficulty in determining to what extent the response of BiP-IRF2BP2 signaling and inflammation to pH requires BiP-binding carboxylates in cells. Although predictions via RoseTTAFold and molecular docking formed a conceptual framework for our study, future work should experimentally resolve the structures of complexes of BiP with IRF2BP2 or BiP-binding carboxylates. Further, based on these potential experimental structural data, establishing rigorous plan of mutagenesis whose possibility to induce large structural change is minimized, is needed to test the importance of the specific binding of BiP with L-malate and carboxylate analogues in the effect of malate and pH fluctuations.

## Materials and methods

All mice were housed in specific-pathogen-free (SPF) facility under conditions of a 12 h light/dark cycle, controlled temperature (23 ± 1 °C), relative humidity (55% ± 5%) with food and water ad libitum. The animal experiments were approved by the Institutional Animal Care Use Committees at Army Medical University (AMUWEC20207065).

### Mouse breeding and genotyping

Wild-type male C57BL/6 J and DBA/1 J mice were obtained from ENSIWEIER Company. The *Hspa5*^f/f^ and *Irf2bp2*^f/f^ mice were generated using the CRISPR/Cas9 system in the C57BL/6 J mouse background. Briefly, for *Hspa5*, an *Hspa5* donor vector containing flox sites flanking exons 5-9 of *Hspa5* was created. Two sgRNA sites targeting intron 4 and the 3’UTR were transcribed in vitro. The sgRNA targeting intron 4 was 5’-AGTTAAGATTGAAAGGTTTCTGG-3’, and the sgRNA targeting the 3’UTR was 5’-ATTGAGAAAAAGGTGGGTCAGGG-3’. The strategy for Irf2bp2 was similar to that for *Hspa5*. floxed mice, in which exons 1-2 of *Irf2bp2* were flanked with loxP recombination sites, were generated. Briefly, an *Irf2bp2* donor vector containing flox sites flanking exons 1-2 of *Irf2bp2* was created. Two sgRNA sites targeting the 5’ upstream sequence and 3’UTR were transcribed in vitro. The sgRNA targeting the 5’ upstream sequence was 5’-GCTGCACACCTGCGGCGCTAGGG-3’, and the sgRNA targeting the 3’UTR was 5’-GAAATGTAGACTTCATAACATGG-3’. Thereafter, the donor vector with two gRNAs and *Cas9* mRNA was microinjected into fertilized C57BL/6 J eggs. F0 generation mice, which were positive for homologous recombination, were identified by long PCR spanning the 5’ or 3’ homologous arm. The positive founder mice were mated to WT C57BL/6 J mice to obtain *Hspa5* or *Irf2bp2* flox heterozygous mice. The mice were generated by Shanghai Model Organisms Center, Inc. (Shanghai, China). *Hspa5*^f/f^; *Lyz2*-Cre and *Irf2bp2*^f/f^; *Lyz2*-Cre mice were obtained by crossing with *Lyz*2-Cre mice from the Jackson Laboratory. Mice between 6 and 12 weeks of age were used for the experiments.

### Reagents

Metabolite candidates in screening assay were used at the concentration of 6 mM. Phenyllactate, L-malate, cis-aconitate, leucine, citrate, uracil, guanosine, serine, oxaloacetate, fructose 6-phosphate, pyruvate, uridine, UMP, phosphorylethanolamine, ornithine, threonine, glutamine, ATP, GDP, argininosuccinate, citrulline, cysteine, isocitrate, GMP, IMP, α-ketoglutarate, ribose 5-phosphate, aspartate, NAD ^+^ , PRPP, GTP, and Flavin mononucleotide were purchased from MCE. PRPP was purchased from Cayman. Alanine, methylmalonate, and glucose were purchased from Selleck. BPG, acetyl-CoA, argininosuccinate, and glucose 6-phosphate were purchased from Sigma. Small molecule compounds used in SPR assay: L-malate (#02288, Sigma), D-malate (#46940-U, Sigma), Succinate (#14079, Sigma), 3-Hydroxybutyrate (#HY-113378, MCE), L-2-hydroxybutyrate acid (#HY-W018499, MCE), L-2-hydroxyglutarate (#90790, Sigma), Malonate (#792535, Sigma).

### Generation and treatment of BMDMs

Bone marrow cells from the femur and tibia of 6–10-week-old male C57BL/6 J mice were differentiated in the presence of recombinant mouse M-CSF (20 ng/mL; PeproTech) in DMEM (containing 10% fetal calf serum and 1% penicillin/streptomycin) for 5–7 days. For experiments, the cells were seeded at a concentration of 0.5 × 10^6^ cells per ml in tissue culture plates of various formats. The cells were treated with L-malate (0.5 - 6 mM, #02288, Sigma), D-malate (0.5 mM, M109342, Aladdin), Dimethyl L-malate (0.5 -3 mM D806475, MACKLIN), monensin sodium salt (0.1 - 1 μM, #HY-N0150, MCE), NH_4_Cl (15 - 30 mM, #HY-Y1269, MCE), sodium acetate (20 mM, #S2889, Sigma), H89 (20 μM, #S1582, Selleck), Tunicamycin (0.1 μM, #654380, Sigma), Thapsigargin (0.1 μM, #HY-13433, MCE), Arachidonic acid (200 μM, #HY-109590, MCE), DCPIB (100 μM, #HY-103371, MCE), CHX (5 μg/mL, #S7418, Selleck), MG-132 (5 μM/0.5 μM, #HY-13259, MCE), bafilomycin A1 (100 nM, #HY-100558, MCE), heptelidic acid (1 μM, #14079, Cayman Chemical), fumarate hydratase-IN-1 (25 μM, #HY-100004, MCE), LPS (100 ng/mL, #L4524, Sigma), 4μ8C (IRE1 Inhibitor III) (100 μM, #HY-19707, MCE), GSK2606414 (10 μM, #S7307, Selleck), and ceapin-A7 (8 μM, #HY-108434, MCE) as indicated.

### Generation and treatment of peritoneal macrophages

6–10-week-old male C57BL/6 J mice were intraperitoneally injected with 3 mL 3% thioglycolate solution 3 days in advance. Mice were euthanized with carbon dioxide. Then, the peritoneum was opened to maximize ascites collection using instruments under sterile conditions. Then erythrocytes were removed from the ascites, and the remaining cells were centrifuged and resuspended in DMEM (containing 10% fetal calf serum and 1% penicillin/streptomycin). And these cells were cultured at 37 °C with 5% CO_2_. After 2 h, the adherent cells were perceived as peritoneal macrophages. The cells were treated with MG-132 (5 μM, #HY-13259, MCE), cellular pH calibration buffer (#P35379, thermo scientific), Nigericin sodium salt (10 μM, # HY-100381, MCE), Valinomycin (10 μM, #HY-N6693, MCE).

### PBMC isolation

Peripheral blood was collected in EDTA-coated tubes. After centrifugalization at 3000 × *g* for 10 min at room temperature, plasm was collected and stored. The remaining blood cells were diluted one-fold with phosphate-buffered saline (PBS) and were layered gently on top of half amount of Ficoll-Paque PREMIUM 1.084 (#17544602, GE Healthcare) in a 15 mL tube. After centrifugation (450 g, 20 min), PBMCs were concentrated in the second layer from the top to the bottom and was collected with pipettes and then stored in another 15 mL tube. Collected PBMCs were washed twice with PBS and centrifuged at 300 x g for 10 min. The isolated PBMCs were resuspended in freezing solution (90% FBS, 10% DMSO) and stored under liquid nitrogen for future use.

### Intracellular pH measurement

Intracellular pH was determined using the ratiometric fluorescent intracellular pH probe 2′,7′-bis-(2-carboxyethyl)-5-(and-6)-carboxyfluorescein acetoxymethyl ester (BCECF-AM)^141^ according to the protocol of the commercial kit (#B1150, Thermo Scientific). Before the measurement of Intracellular pH, BMDMs and Raw264.7 cells were implanted in 96 Wells dedicated to fluorescence intensity measurement at 70%-80% confluence. After the treatments as described aforementioned, 2 μM BECEF-AM was added into 96 Wells and incubated for 45 min at 37 °C. The fluorescence intensity was quantified by alternating excitation at two different wavelengths under λ1 = 490 nm and λ2 = 440 nm, while fixed emission at 535 nm respectively. Then the fluorescence intensity was used to calculated the absolute value of intracellular pH based on the standard curve which was established as described below. With the aim of quickly balancing and equalizing intracellular and extracellular pH values, the cellular pH calibration buffer, valinomycin (10 μM) and nigericin (10 μM) were used to establish the standard curve in accordance with the protocol of the commercial kit (#P35379, Thermo Scientific).

### Glyceraldehyde-3-phosphate dehydrogenase (GAPDH) activity assay

The GAPDH activity analysis was compliant with the instructions provided with the kit (#ab204732, Abcam). BMDMs were cultured in 6-well plates at 1 ×10^6^ cells per well and treated with heptelidic acid and LPS for 16 h. After cracking and centrifugation, 20 µL of supernatant was used as the sample to be tested. The 50 µL reaction system was proportionally configured in advance before the formal measurements, and this system was composed of 46 µL of assay buffer IV/GAPDH assay buffer, 2 µL of developer solution III/GAPDH developer, and 2 µL of glyceraldehyde-3-Phosphate/GAPDH substrate. Then, 20 µL of sample was combined with 50 µL of the reaction mix, and the mixture was loaded onto a 96-well plate to measure the absorbance at OD450 nm in the kinetic mode at 37 °C.

### Quantitative real-time reverse transcription PCR

Total RNA was extracted from cells or tissues with a RNeasy Micro Kit (QIAGEN). cDNA synthesis was carried out with a RevertAid First Strand cDNA Synthesis Kit (#K1622, Thermo Scientific) according to the manufacturer’s instructions. Real-time quantitative PCR was performed with PowerUP SYBR Green Master Mix (Thermo Fisher Scientific) and the CFX96 Touch System (Bio-Rad). 18S rRNA was used as an internal control for normalization. The primers for qRT‒PCR are listed below: 5’-TGCCACCTTTTGACAGTGATG-3’ and 5’-TGATGTGCTGCTGCGAGATT-3’ for *Il1b*; 5’-CTCCCAACAGACCTGTCTATAC-3’ and 5’-CCATTGCACAACTCTTTTCTCA-3’ for *Il6*; 5’-CCCTCACACTCACATCATCTTCT-3’ and 5’-GCTACGTGGGCTACAG-3’ for *Tnfa*; 5’-CACTGTCGAGTCGCGTCC-3’ and 5’-TCATCCATGGCGAACTGGTG-3’ for *Actb*; 5’-AGGTCGGTGTGAACGGATTTG-3’ and 5’-TGTAGACCATGTAGTTGAGGTCA-3’ for *Gapdh*; 5’-AGCAAGTTCAAGAAGGAGCCG-3’ and 5’-GCTGTCCTTGCAACTGCTTTA-3’ for *Irf2bp2*; 5’-CCTGAGCCCGGAGGAGAA-3’ and 5’-CTCGAGCAGTCTGCGCTG-3’ for *Xbp1*; 5’-GAGTCCGCAGCAGGTG-3’ and 5’-GTGTCAGAGTCCATGGGA-3’ for *Xbp1s*.

### Immunoblot analysis

Tissues or cell proteins were extracted with M-PER (Thermo Fisher Scientific) supplemented with 1× cocktails containing protease and phosphatase inhibitors (Roche). The protein concentrations were determined using a BCA kit (Thermo Fisher Scientific). The extracted proteins were resolved by 4-12% SDS‒PAGE and then transferred onto a polyvinylidene difluoride (PVDF) membrane using either a wet or semidry transfer system. The membranes were blocked with 5% (w/v) dried milk in TBS-Tween (TBST) for 60 min at room temperature. The membranes were incubated with primary antibodies [anti-beta-actin (#8457, Cell Signaling; R23613, ZENBIO), anti-IRF2BP2 (#18847-1-AP, Proteintech), anti-beta-tubulin (#2146, Cell Signaling), anti-IL-1-beta (#12507, Cell Signaling), anti-BiP (#ab21685, Abcam), anti-ATF6 (#24169-1-AP, Proteintech), anti-eIF2α (#ab169528, Abcam), anti-P-eIF2α (9721S, Cell Signaling)] at 4 °C overnight and then with horseradish peroxidase-conjugated secondary antibodies (CST) for 2 h at room temperature. The blots were developed with a Western blotting detection kit (Advansta) for 1 min, and the bands were visualized using Fusion FX6-XT (VILBER).

### ELISA assay

Levels of IL-1β, IL-6 and TNF-α were detected in BMDMs medium or serums isolated from murine models using ELISA kits, in accordance with the manufacturer’s instructions [BGK10749 (IL-1β), BGK08505 (IL-6), BGK06804 (TNF-α) Biogem, originally known as a part of Peprotech].

### Immunofluorescence

BMDMs were washed in PBS and fixed with 4% paraformaldehyde at room temperature for 15 min. The cells were then blocked for 30 min with 5% bovine serum albumin (BSA) diluted with 0.3% Triton X–100. The cells were incubated with antibodies against BiP (1:100, ab212054, Abcam) and Irf2bp2 (1:100, HPA027815, Sigma) at 1:50 dilution overnight at 4 °C in a dark humidified chamber. After washing, the cells were incubated with a fluorophore-conjugated secondary antibody for 60 min. The cells were then counterstained with DAPI in the dark for 5 min. The stained samples were visualized and photographed by laser-scanning confocal microscopy (Olympus, IXplore SpinSR).

### Proximal ligation assay

A proximal ligation assay was conducted as previously described.^142^ Briefly, BMDMs were seeded on a chamber slide. The cells were fixed with 4% PFA, and washed three times with PBS before performing the PLA experiment according to the manufacturer’s instructions provided with the Duolink In Situ Red Starter Kit Mouse/Rabbit (Sigma Aldrich). Subsequently, the nonspecific cellular antigens were blocked with blocking solution for 1 h at 37 °C and then incubated with antibodies against BiP (1:100, ab212054, Abcam) and Irf2bp2 (1:100, HPA027815, Sigma) alone or simultaneously in the Probemaker PLA Probe Diluent overnight at 4 °C. Thereafter, the PLA probes were linked to corresponding antibodies and amplified in relevant buffer at 37 °C. Duolink In Situ Mounting Medium with DAPI (Sigma) was used to mount the slides with a cover slip. Images were acquired using a confocal microscope (Olympus, IXplore SpinSR).

### DARTS

Drug affinity responsive target stability (DARTS) assay was adapted from Zhang et al..^45^ Raw 264.7 (washed by pre-chilled PBS twice) were lysed using M-PER (Thermo Scientific, 78501) supplemented with the addition of protease inhibitors (Roche) and phosphatase inhibitors (Roche). TNC buffer (50 mM Tris-HCl pH 8.0, 50 mM NaCl, 10 mM CaCl2) was added to the lysate and centrifuged at 14,000 at 4°C for 10 min. Protein concentration of the supernatant was determined using the BCA Protein Assay kit. Proteins were diluted to the same final volume and protein concentration. To add L-malate (1 mM) to the supernatant, 100 mM L-malate solution with pH adjusted to 7.0 or vehicle control (H_2_O, pH 7.0) was added to the supernatant at 99 times the volume, followed by incubation with vehicle or L-malate for 1 h at room temperature. Digestion was performed using Pronase (Roche) at room temperature for 15 min and was stopped using excess protease inhibitors with immediate transfer to ice. The resulting digests were separated by SDS-PAGE 10% gel. Bands with increased staining were excised from the lane of L-malate-treated group and the matching area of the vehicle lane, and then the gels were prepared for LC-MS/MS analysis.^143^ LC separation was performed online on an EASY-nLC 1000 (Thermo Scientific). Peptides were gradient eluted directly to an Orbitrap Fusion Lumos mass spectrometer (Thermo Fisher). High resolution full MS spectra were acquired every second with a resolution of 240,000, an AGC target of 1e6, with a maximum ion injection time of 50 ms, and scan range of 400 to 1500 m/z. Following each full MS data-dependent HCD MS/MS scans were acquired in the ion trap using the rapid scan mode with an AGC target of 6e4, maximum ion time of 18 ms, one microscan, 0.7 m/z isolation window, normalized collision energy (NCE) of 30, fixed first mass 110 m/z and dynamic exclusion for 20 s. Only ions with a charge state of 2-7 were allowed to trigger an MS2 scan. The MS/MS spectra were searched against a Uniprot (www.uniprot.org) mouse protein database with common lab contaminants added using Sequest within Proteome Discoverer 1.4 (Thermo Fisher). The search parameters were as follows: mass accuracy better than 10 ppm for MS1 and 0.4 Da for MS2, two missed cleavages, fixed modification carbamidomethyl on cysteine, variable modification of oxidation on methionine and deamidation on asparagine and glutamine. The data was filtered using a 1% FDR cut off for peptides and proteins against a decoy database and only proteins with at least 2 unique peptides were reported.

### Macrophage polarization assay

The polarization of BMDMs was induced for M1 activation by treatment with LPS (100 ng/mL, Sigma) for 24 h or the indicated periods before further analysis.

### Flow cytometry

Flow cytometry was conducted as previously described.^144^ For the detection of macrophage polarization, mouse claws or colorectal resection samples were mechanically cut into 1-mm^3^ pieces with scissors and digested with PBS containing Ca/Mg, 10 μg/ml DNase I (Sigma Aldrich), and 1 mg/ml collagenase IV (Sigma Aldrich) for 30 min at 37 °C. The solution was stirred on a magnetic stirrer. After digestion, the tissue suspension was filtered through a 70-μm filter and centrifuged at 500 × g for 5 min. The blood cells were lysed in RBC lysis buffer (Sigma Aldrich) for 10 min and then mixed with PBS to stop the reaction. The samples were then resuspended in FACS buffer (PBS with 2% FBS) for staining. The spleen was separated by a 70-μm filter and washed with FACS buffer. After centrifugation at 500 × g for 5 min, the blood cells were lysed as described above. The spleen sample was eventually resuspended in FACS buffer. The following antibodies were used: PerCP/Cy5.5 anti-mouse CD45 antibody (#103132, BioLegend), APC/Cy7 anti-mouse F4/80 (#123118, BioLegend), APC anti-mouse CD206 (#141708, BioLegend), FITC anti-mouse CD45R (#103206, BioLegend), FITC anti-mouse CD3 (#100203, BioLegend), and PE anti-mouse CD11c (#117308, BioLegend). Macrophages were marked based on the phenotype CD3^-^CD45R^-^CD45^+^ F4/80^+^ . M1-like cells were defined by the phenotype CD3^-^CD45R^-^CD45^+^F4/80^+^CD206^-^CD11^+^ .

In the cell apoptosis assay, BMDMs were collected, washed twice with cold PBS and stained with the APC Annexin V Apoptosis Detection Kit with PI (#640932, BioLegend). The apoptosis rate was detected by flow cytometry after incubation at room temperature for 15 min in darkness.

### Collagen antibody induced arthritis (CAIA) model

CAIA was induced as previously described.^145^ On day 0, 6–8-week-old male DBA/1 mice were injected intravenously with 80 μg/mg 5-clone cocktail (Catalog # 53100, Chondrex, LLC, Seattle, WA). CAIA mice were given an intraperitoneal injection of PBS, 200 mg/kg malate, or 400 mg/kg malate every day, starting on day 2. On day 3, an additional injection of 1.5 μg/mg LPS injection was given. Severity of arthritis was assessed by the qualitative clinical score and the swelling score via determining paw thickness using a Mitutoyo loop handle dial thickness gauge with a caliper.

For histopathological assessments, the ankle joints of CAIA mice treated with PBS, 200 mg/kg malate, or 400 mg/kg malate were sectioned, deparaffinized, rehydrated, and stained with hematoxylin and eosin. Slides were scanned using an Olympus VS200 slide scanner and visualized with OlyVIA version 3.2 software (Olympus).

### Endotoxin-induced model of sepsis

For survival studies, the mice were intragastrically administered L-malate (50, 100, 200, or 400 mg/kg) or PBS for 2 h, sepsis was induced by the injection of 50 mg/kg LPS, and the survival was monitored every 3 h for up to 24 h. The mice were culled immediately at a humane end-point.

### Induction and assessment of experimental (acute) colitis

Colitis was induced by colitis-grade dextran sodium sulfate (DSS; 36–50 kDa; 3% weight/volume; MP Biomedicals) added to the drinking water for 7 days. The control mice received standard drinking water. C57 mice were intragastrically administered PBS, 50 mg/kg L-malate, 100 mg/kg L-malate, or 200 mg/kg L-malate every day starting 2 days after the addition of DSS. The disease activity index (DAI) was calculated by scoring the percent weight loss, intestinal bleeding [no blood, occult blood (hemoccult + ), or gross blood], and stool consistency (normal stool, loose stool, or diarrhea), as previously described (*28*). After 7 days of DSS treatment, the mice were sacrificed. For the histological assessment of colitis, colon specimens were fixed in 4% paraformaldehyde (PFA) and embedded in paraffin. Four-micrometer tissue sections were stained with H&E.

### Coimmunoprecipitation (Co-IP)

Co-IP was conducted as previously described.^146^ Briefly, cell lysis buffer (87787, Thermo) for Western and IP was used for the protein extraction of Raw264.7 on ice for 15 min, and then the lysate was quantified. Next, magnetic beads (Sera-Mag SpeedBead Protein A/G, 17152104010150, GE) which were incubated with 4-25 μg of antibodies against BiP (PA5-19503, Invitrogen) or IRF2BP2 (18847-1-AP, ProteinTech) at 4 °C overnight, were washed three times with lysis buffer, and then added into 500-1000 μg of cellular protein extract and co-incubate with extract at 4 °C overnight. Thereafter, these magnetic beads were washed three times with lysis buffer. The bound protein and input samples were examined by Western blotting with anti-BiP (PA5-19503, Invitrogen), anti-IRF2BP2 (PA5-55700, Invitrogen), anti-ubiquitin (3933, CST) or anti-beta-actin (#8457, Cell Signaling; R23613, ZENBIO) antibodies.

### Human proteome microarray

Analysis of protein‒protein interactions using a Human Proteome Microarray (CDI Laboratories) was performed as described previously with some modifications.^147^ In brief, a total of 25 μg of recombinant human BiP protein (#ab78432, Abcam) was added to 67 μL of labeling buffer, and then 3 μL of 10 μg/μL Biotin Reagent diluted in N, N-Dimethylformamide was added. Subsequently, the above mixture was shaken at room temperature for 2 h, once every 10 min. Finally, 30 μL of stop solution was added to the mixture, followed by shaking at room temperature for 30 min. Three slides of the proteome microarray were preblocked with blocking buffer (PBS with 0.1% Tween 20 and 5% BSA) for 1.5 h and then incubated with biotin (control), biotin-labeled BiP protein at 1 μg/ml, or a mixture of L-malate (1 mM) and biotin-labeled BiP protein for 1 h at room temperature. The slides were then subjected to three 5-min washes with 1× phosphate-buffered saline buffer containing Tween-20 (PBST) and subsequently to two 5-min washes with distilled water. The microarrays were further incubated with Cy5-conjugated streptavidin diluted in 1× detection buffer (1:1000) for 20 min at room temperature and washed. The microarrays were then spun to dryness and scanned with GenePix 4000B (Axon Instruments, Sunnyvale, CA, USA) for visualization. The proteome microarray data analysis included four steps: image scan, background correction, interchip normalization and differential spot statistics.^60^ (1) Image scan: The median foreground and background intensities for each spot in the protein microarrays were obtained with GenePix Pro 6.0 software. We manually checked all the spots on the chips and adjusted the size and position of the spots with dust or specks. (2) Background correction: We calculated the signal intensity of each spot, which was defined as the foreground median intensity divided by its local background median intensity, named the “I” value. (3) Interchip normalization: The values of “I” between the “BiP” and “BiP+MA” samples were normalized using the R package “limma” with the function “normalizeBetweenArrays”.^148^ The aim of normalization is to reduce the effect of systematic errors caused by experimental factors and make the microarrays comparable in order to allow the discovery of actual biological differences. (4) Differential spot statistics: Differential spots were defined as those with a difference in normalized I values >2 between the “BiP+MA” (normalized) and “BiP” (normalized) samples.

### Measurement of L-malate and fumarate by LC–MS/MS

The sample extracts were collected from BMDMs or PBMCs washed with ice cold DPBS for three times, and were analyzed by tandem mass spectrometry (MS/MS, QTRAP® 6500+ with ultra-performance liquid chromatography (UPLC, ExionLC™ AD)). L-malate was analyzed by scheduled multiple reaction monitoring. The metabolites were isolated using an ACQUITY UPLC amino column (2.1 mm × 100 mm, 1.7 μm) with a maintained column temperature of 40 °C. The mobile phase comprised 10 mmol/L ammonium acetate, 0.3% ammonia solution, and 90% acetonitrile water. The flow rate was set to 0.4 mL/min with a sample volume of 2 μL. The MS operating parameters were as follows: ion spray voltage, -4500 V; temperature, 550 °C°C; curtain gas, 35; ion source gas 1, 50; ion source gas 2, 60; collision gas, 8; entrance potential, 10; collision cell exit potential, 10; and scan type: multiple reaction monitoring (MRM). The quantitative standard curve was prepared for LC‒MS analysis. Standard linear regression curves were drawn with the mass of the analyte peak area on the vertical axis and the analyte concentration on the horizontal axis.

### L-malate metabolite analysis and stable isotope tracing by GC-MS

LPS-stimulated BMDMs were treated with deuterium-labeled L-malate (6 mM, 8 h) (MCE, HY-Y1069S) in a pH_e_-controlled condition. After incubation with medium, cells were washed with ice-cold PBS for three times and frozen with liquid nitrogen. For the cultured cell tracing experiment, samples preparation and methods of metabolomics analysis were performed as described previously.^149,150^ Natural isotope abundance was corrected using IsoCor v.2.0.^151^

### Measurement of glycolytic rate by Seahorse assay

BMDMs extracellular acidification rate (ECAR) were measured using the Seahorse XFe96 Extracellular Flux Analyzer (Seahorse Bioscience, Agilent) according to manufacturer’s instructions of Seahorse XF glycolytic rate assay kit (103344, Agilent). BMDMs were seeded into 96-well XF cell culture microplate in induction medium (3.3 × 10^4^ per well). Before the Seahorse assay, the cells were first treated by LPS with or without L-malate (6 mM, pH 6.7) for different times, and then remove the medium. Wash once with warmed assay medium (XF DMEM medium, pH 7.4, with 1 mM pyruvate, 2 mM glutamine and 10 mM glucose), and incubate with warmed assay medium at 37 °C in a non-CO_2_ incubator for 60 min. Then, remove the assay medium, and add fresh, warm assay medium and injected Rot/AA and 2-DG (2-deoxy-glucose) sequentially. The glycolytic proton efflux rate (glycoPER), basal glycolysis rate and % PER from glycolysis were calculated using Wave Desktop 2.6 (Agilent Technologies).

### Surface plasmon resonance (SPR) analysis

The SPR experiments were performed using a Biacore 8 K system at 25 °C with a CM5 sensor chip. The recombinant BiP protein (#ab78432, Abcam) or BiP fragments for SPR analysis were diluted into NaAc (pH 4.0) and immobilized on the CM5 chip using EDC/NHS regents; a blank channel was used as a negative control. Small molecule compounds and recombinant IRF2BP2 protein (OriGene) diluted with HBS-EP (or HBS-EP with small molecule compounds) were allowed to flow over the chip surface, and the response units were measured. The binding kinetics constants were analyzed using Bia-evaluation analysis software (Cytiva).

### Preparation of BiP protein

The appropriate plasmid pET28a-HSPA5 was transformed into calcium chloride-competent *E. coli* BL21 (DE3), and the cells were then induced by 1 mM IPTG at 37 °C for 3 h. Recombinant protein was purified from the supernatant of the bacterial lysate using an affinity HisTrap^TM^ HP column (Cytiva). After the column was washed with 10 mL of equilibrium buffer, the protein was eluted by imidazole and dialyzed in PBS (pH 7.4). The purified product was analyzed by SDS‒PAGE.

### Molecular docking simulation

The molecular docking of malate to HSPA5 was completed with the software package described by Schrödinger (2015). The crystal structure (PDB ID: 5E84) was first prepared using the Protein Preparation Wizard module. During the process, nonessential components in the crystal structure were deleted, and only the protein structure was retained. The force field of OPLS3 was selected to perform restricted energy optimization. The RMSD value of hydrogen atom variation was limited to 0.30 Å. The obtained protein structure was used for further docking simulation. The binding sites were detected using the Sitemap module. According to the identified sites, grid files were generated. Malate was then processed to generate low-energy 3D structures using the LigPrep module, and possible protonation states were generated at pH 7.0 ± 0.5 with the force field of OPLS3. The original chirality of malate was maintained, and isomers were not generated. The structure was docked into each binding site using the Glide extra precision (XP) mode with default settings. The docking conformations were output based on their docking score.

The structure of IRF2BP2 was predicted using RoseTTAFold according to its amino acid sequence. Based on the evaluation results and error estimate values, Profiles-3D and Ramachandran plot, model 1 was ultimately selected for protein‒protein docking with HSPA5 using ZDOCK implemented in Discovery Studio 2019. The force field of CHARMM was set for protein‒protein docking. The top 2000 poses of 100 clusters were set to output based on the ZDOCK score. The other parameters were set as defaults.

### Generation of *Mdh2*^−/−^ and *Hspa*5^+/−^ Raw264.7 cells by CRISPR‒Cas9

*Hspa5*^**+/−**^ and *Mdh2*^**−/−**^ cell lines derived from Raw264.7 cells were constructed using the CRISPR/Cas9 system. Gene-specific sgRNAs were designed to target gene coding regions as follows: *Hspa5* sgRNA sense, 5′-AGCGCCTCATCGGACGCACT-3′ and antisense, 5′-AGTGCGTCCGATGAGGCGCT-3′; and *Mdh2* sgRNA sense, 5′-GTGTGTGAGCGATATCGTAG-3′ and antisense, 5′-CTACGATATCGCTCACACAC-3′. The sgRNA was cloned and inserted into the pHBLV-U6-gRNA-EF1-CAS9-PURO vector (Hanbio Biotechnology). The cotransfection of CRISPR plasmids, psPAX2 and pMD2G into HEK293T cells (ATCC) was performed to produce the lentivirus. Raw264.7 cells were then infected with lentivirus using polybrene (6 μg/mL) for 48 h and then selected with 2 μg/mL puromycin (Gibco) for 2 days.

### NaOH-induced colitis in mice

The procedure was performed as described^86^ with some modifications. Before the start of the study, preliminary experiments were used to determine the dose of NaOH. Twenty-four hours before the procedure, food was removed from the cage, and the mice were only allowed to drink water. On the day of induction, all the mice were anesthetized, and a 4-mm-diameter enema tube was inserted rectally until the tip was 4 cm proximal to the anus. Then, 0.3 ml of 0.9% saline or 0.3% NaOH solution was administered slowly to the *Hspa5*^f/f^ or *Hspa5*^f/f^; *Lyz2*-Cre mice. Thereafter, all the mice were maintained in a head-down position for 5 min to limit expulsion of the solution. On the morning of the 3rd or the 6th day, all the mice were sacrificed. Laparotomy and total colectomy were performed. The lumen of the resected specimen was irrigated with 0.9% NaCl. The distal colon segment was then split longitudinally into two pieces and preserved for histological and biochemical analyses.

For histopathological assessment, the colons were fixed in 4% paraformaldehyde for 48 h. Blocks were serially sectioned at a thickness of 5 μm along the cephalocaudal axis of the gut to the level of the lumen. Hematoxylin and eosin (H&E) staining procedures were performed according to the standard protocol provided with the stain kit (G1120, Solarbio). IHC staining of IRF2BP2 was performed with anti-IRF2BP2 antibody (1:200, 18847-1-AP, ProteinTech) and detected using horseradish peroxidase-conjugated secondary antibodies. Digital light microscopy images were recorded with a Zeiss Axio Imager.A1 microscope (Thornwood, NY), AxioCam MRc5 camera, and AxioVision 4.7.1 imaging software (Carl Zeiss Microimaging).

### RNA sequencing

Total RNA was isolated using TRIzol reagent (Invitrogen, Carlsbad, CA, USA). All RNA samples were determined to have A260/280 values ≥ 1.8 (Nanodrop); the samples for RNA-seq had RIN values >7 (BioAnalyzer, Agilent). Poly (A) RNA was purified from 1 μg of total RNA, fragmented, and then reverse-transcribed to create cDNA using SuperScript™ II Reverse Transcriptase (Invitrogen, cat. 1896649, USA) to construct a library. Then, 2×150-bp paired-end sequencing (PE150) on an Illumina NovaSeq™ 6000 (LC-Bio Technology Co., Ltd., Hangzhou, China) was performed following the vendor’s recommended protocol.

### Bioinformatics analysis

Fastp software was used to remove the reads that contained adaptor contamination, low-quality bases, and undetermined bases with the default parameters. HISAT2 was used to map reads to the reference genome of the mouse: Mus_musculus_Ensemble_101. StringTie was used to determine the expression levels of mRNAs by calculating FPKM values (FPKM = [total_exon_fragments / mapped_reads (millions) × exon_length (kB)]). We considered genes to be significantly differentially expressed based on a threshold of <0.05 in *P* value obtained from a parametric F test comparing nested linear models using R package edgeR, and a threshold of >1 in log1.3 |fold-change| or log2 |fold-change| when performing the GO analysis or all other analysis, respectively. A volcano plot was generated to visualize the differentially expressed genes between groups, and genes associated with the UPR were labeled. KEGG pathway enrichment analysis was performed. Canonical pathway analysis and upstream regulator predictions were performed using IPA (Qiagen).

### Quantification and statistical analysis

The data are presented as the means ± SEMs. Statistical analyses were performed in Excel, R, and Prism. For comparisons of treatment groups, two-tailed Student’s *t*-test, one-way ANOVA or two-way ANOVA was performed. The data that did not adhere to a normal distribution were analyzed by a two-sided Mann‒Whitney U test. Mantel‒Cox was used for survival analysis. *p* < 0.05 was considered to indicate statistical significance: *p < 0.05, **p < 0.01, and ***p < 0.005. Details of the statistical analyses and “*n*” values are found in the Figure Legends. For in vivo experiments, “n” indicates the number of mice, and in vitro experiments, “n” indicates the number of biological replicates.

## Supplementary information

Supplementary Materials

original WB data

## Data Availability

All data are available in the main text or the supplementary materials. The RNA-seq datasets have been submitted to SRA database (SRA accession: PRJNA893377). The mass spectrometry datasets for DARTS is available at https://massive.ucsd.edu (accession number: MassIVE MSV000090772_reviewer).

## References

[1] Spiller, K. L. & Koh, T. J. Macrophage-based therapeutic strategies in regenerative medicine. *Adv. Drug Deliv. Rev.* **122**, 74–83 (2017). 28526591 10.1016/j.addr.2017.05.010 PMC5690893

[2] Ji, L. et al. Slc6a8-Mediated Creatine Uptake and Accumulation Reprogram Macrophage Polarization via Regulating Cytokine Responses. *Immunity* **51**, 272–284.e277 (2019). 31399282 10.1016/j.immuni.2019.06.007

[3] Ryan, D. G. & O’Neill, L. A. J. Krebs Cycle Reborn in Macrophage Immunometabolism. *Annu Rev. Immunol.* **38**, 289–313 (2020). 31986069 10.1146/annurev-immunol-081619-104850

[4] Hooftman, A. & O’Neill, L. A. J. The Immunomodulatory Potential of the Metabolite Itaconate. *Trends Immunol.* **40**, 687–698 (2019). 31178405 10.1016/j.it.2019.05.007

[5] Mills, E. L. et al. Itaconate is an anti-inflammatory metabolite that activates Nrf2 via alkylation of KEAP1. *Nature* **556**, 113–117 (2018). 29590092 10.1038/nature25986 PMC6047741

[6] Kornberg, M. D. et al. Dimethyl fumarate targets GAPDH and aerobic glycolysis to modulate immunity. *Science* **360**, 449–453 (2018). 29599194 10.1126/science.aan4665 PMC5924419

[7] Bambouskova, M. et al. Electrophilic properties of itaconate and derivatives regulate the IκBζ-ATF3 inflammatory axis. *Nature* **556**, 501–504 (2018). 29670287 10.1038/s41586-018-0052-z PMC6037913

[8] Humphries, F. et al. Succination inactivates gasdermin D and blocks pyroptosis. *Science* **369**, 1633–1637 (2020). 32820063 10.1126/science.abb9818 PMC8744141

[9] Müller, D. N. et al. Sodium in the microenvironment regulates immune responses and tissue homeostasis. *Nat. Rev. Immunol.* **19**, 243–254 (2019). 30644452 10.1038/s41577-018-0113-4

[10] Hotamisligil, G. S. Inflammation, metaflammation and immunometabolic disorders. *Nature* **542**, 177–185 (2017). 28179656 10.1038/nature21363

[11] Solis, A. G. et al. Mechanosensation of cyclical force by PIEZO1 is essential for innate immunity. *Nature* **573**, 69–74 (2019). 31435009 10.1038/s41586-019-1485-8 PMC6939392

[12] Netea, M. G. et al. A guiding map for inflammation. *Nat. Immunol.* **18**, 826–831 (2017). 28722720 10.1038/ni.3790 PMC5939996

[13] McGettrick, A. F. & O’Neill, L. A. J. The Role of HIF in Immunity and Inflammation. *Cell Metab.* **32**, 524–536 (2020). 32853548 10.1016/j.cmet.2020.08.002

[14] Jain, N. & Vogel, V. Spatial confinement downsizes the inflammatory response of macrophages. *Nat. Mater.* **17**, 1134–1144 (2018). 30349032 10.1038/s41563-018-0190-6 PMC6615903

[15] Reddy, A. et al. pH-Gated Succinate Secretion Regulates Muscle Remodeling in Response to Exercise. *Cell* **183**, 62–75.e17 (2020). 32946811 10.1016/j.cell.2020.08.039 PMC7778787

[16] Intlekofer, A. M. et al. L-2-Hydroxyglutarate production arises from noncanonical enzyme function at acidic pH. *Nat. Chem. Biol.* **13**, 494–500 (2017). 28263965 10.1038/nchembio.2307 PMC5516644

[17] Zhang, J. et al. Endothelial Lactate Controls Muscle Regeneration from Ischemia by Inducing M2-like Macrophage Polarization. *Cell Metab.* **31**, 1136–1153.e1137 (2020). 32492393 10.1016/j.cmet.2020.05.004 PMC7267778

[18] Martínez-Reyes, I. & Chandel, N. S. Mitochondrial TCA cycle metabolites control physiology and disease. *Nat. Commun.* **11**, 102 (2020). 31900386 10.1038/s41467-019-13668-3 PMC6941980

[19] Tannahill, G. M. et al. Succinate is an inflammatory signal that induces IL-1β through HIF-1α. *Nature* **496**, 238–242 (2013). 23535595 10.1038/nature11986 PMC4031686

[20] Mogilenko, D. A. et al. Metabolic and Innate Immune Cues Merge into a Specific Inflammatory Response via the UPR. *Cell* **177**, 1201–1216.e1219 (2019). 31031005 10.1016/j.cell.2019.03.018

[21] Mills, E. L. et al. Succinate Dehydrogenase Supports Metabolic Repurposing of Mitochondria to Drive Inflammatory Macrophages. *Cell* **167**, 457–470.e413 (2016). 27667687 10.1016/j.cell.2016.08.064 PMC5863951

[22] Murphy, M. P. & O’Neill, L. A. J. Krebs Cycle Reimagined: The Emerging Roles of Succinate and Itaconate as Signal Transducers. *Cell* **174**, 780–784 (2018). 30096309 10.1016/j.cell.2018.07.030

[23] Pearl, J. I. et al. Role of the Toll-like receptor pathway in the recognition of orthopedic implant wear-debris particles. *Biomaterials* **32**, 5535–5542 (2011). 21592562 10.1016/j.biomaterials.2011.04.046 PMC3716294

[24] Konttinen, Y. T. et al. Macrophage polarization and activation in response to implant debris: influence by “particle disease” and “ion disease”. *J. Long. Term. Eff. Med Implants* **24**, 267–281 (2014). 25747030 10.1615/jlongtermeffmedimplants.2014011355 PMC4373605

[25] Kawai, T. et al. Unresponsiveness of MyD88-deficient mice to endotoxin. *Immunity* **11**, 115–122 (1999). 10435584 10.1016/s1074-7613(00)80086-2

[26] Ip, W. K. E. et al. Anti-inflammatory effect of IL-10 mediated by metabolic reprogramming of macrophages. *Science* **356**, 513–519 (2017). 28473584 10.1126/science.aal3535 PMC6260791

[27] Xia, B. et al. Regular Use of Proton Pump Inhibitor and the Risk of Inflammatory Bowel Disease: Pooled Analysis of 3 Prospective Cohorts. *Gastroenterology* **161**, 1842–1852.e1810 (2021). 34389338 10.1053/j.gastro.2021.08.005

[28] Wang, G. et al. Nonleaching Antibacterial Concept Demonstrated by In Situ Construction of 2D Nanoflakes on Magnesium. *Adv. Sci. (Weinh.)* **7**, 1902089 (2020). 31921567 10.1002/advs.201902089 PMC6947590

[29] West, M. A. et al. Mechanism of decreased in vitro murine macrophage cytokine release after exposure to carbon dioxide: relevance to laparoscopic surgery. *Ann. Surg.* **226**, 179–190 (1997). 9296512 10.1097/00000658-199708000-00010 PMC1190953

[30] Grabowski, J. et al. Tumor necrosis factor expression is ameliorated after exposure to an acidic environment. *J. Surg. Res.* **173**, 127–134 (2012). 20888586 10.1016/j.jss.2010.08.005 PMC3017641

[31] Hanly, E. J. et al. CO2 Pneumoperitoneum modifies the inflammatory response to sepsis. *Ann. Surg.* **237**, 343–350 (2003). 12616117 10.1097/01.SLA.0000055271.58945.E2 PMC1514307

[32] Öörni, K. et al. Acidification of the intimal fluid: the perfect storm for atherogenesis. *J. Lipid Res.* **56**, 203–214 (2015). 25424004 10.1194/jlr.R050252 PMC4306676

[33] Farr, M. et al. Significance of the hydrogen ion concentration in synovial fluid in rheumatoid arthritis. *Clin. Exp. Rheumatol.* **3**, 99–104 (1985). 4017318

[34] Swallow, C. J., Grinstein, S. & Rotstein, O. D. Lipopolysaccharide impairs macrophage cytoplasmic pH regulation under conditions simulating the inflammatory microenvironment. *J. Leukoc. Biol.* **52**, 395–399 (1992). 1402389 10.1002/jlb.52.4.395

[35] Orlinska, U. & Newton, R. C. Effects of intracellular ions on interleukin-1 beta production by lipopolysaccharide-activated human monocytes. *Am. J. Physiol.* **263**, C1073–C1080 (1992). 1443100 10.1152/ajpcell.1992.263.5.C1073

[36] Chen, H. H. et al. IRF2BP2 Reduces Macrophage Inflammation and Susceptibility to Atherosclerosis. *Circ. Res.* **117**, 671–683 (2015). 26195219 10.1161/CIRCRESAHA.114.305777

[37] Ellegast, J. M. et al. Unleashing Cell-Intrinsic Inflammation as a Strategy to Kill AML Blasts. *Cancer Discov.* **12**, 1760–1781 (2022). 35405016 10.1158/2159-8290.CD-21-0956 PMC9308469

[38] Sawyer, L. & James, M. N. Carboxyl-carboxylate interactions in proteins. *Nature* **295**, 79–80 (1982). 7057876 10.1038/295079a0

[39] Chen, X. et al. pH sensing controls tissue inflammation by modulating cellular metabolism and endo-lysosomal function of immune cells. *Nat. Immunol.* **23**, 1063–1075 (2022). 35668320 10.1038/s41590-022-01231-0 PMC9720675

[40] Mogi, C. et al. Involvement of proton-sensing TDAG8 in extracellular acidification-induced inhibition of proinflammatory cytokine production in peritoneal macrophages. *J. Immunol.* **182**, 3243–3251 (2009). 19234222 10.4049/jimmunol.0803466

[41] Hahn, E. L., Halestrap, A. P. & Gamelli, R. L. Expression of the lactate transporter MCT1 in macrophages. *Shock* **13**, 253–260 (2000). 10774612 10.1097/00024382-200004000-00001

[42] Goncalves, R. L. et al. Sites of superoxide and hydrogen peroxide production by muscle mitochondria assessed ex vivo under conditions mimicking rest and exercise. *J. Biol. Chem.* **290**, 209–227 (2015). 25389297 10.1074/jbc.M114.619072 PMC4281723

[43] Zhao, Y. et al. Malate transported from chloroplast to mitochondrion triggers production of ROS and PCD in Arabidopsis thaliana. *Cell Res.* **28**, 448–461 (2018). 29540758 10.1038/s41422-018-0024-8 PMC5939044

[44] Lomenick, B. et al. Target identification using drug affinity responsive target stability (DARTS). *Proc. Natl Acad. Sci. USA* **106**, 21984–21989 (2009). 19995983 10.1073/pnas.0910040106 PMC2789755

[45] Zhang, C. et al. A Semi-Quantitative Drug Affinity Responsive Target Stability (DARTS) assay for studying Rapamycin/mTOR interaction. *J Vis Exp.* **150**, e59656 (2019). 10.3791/59656 31524870

[46] Chin, R. M. et al. The metabolite α-ketoglutarate extends lifespan by inhibiting ATP synthase and TOR. *Nature* **510**, 397–401 (2014). 24828042 10.1038/nature13264 PMC4263271

[47] Yang, J. et al. Conformation transitions of the polypeptide-binding pocket support an active substrate release from Hsp70s. *Nat. Commun.* **8**, 1201 (2017). 29084938 10.1038/s41467-017-01310-z PMC5662698

[48] Yang, J. et al. Close and Allosteric Opening of the Polypeptide-Binding Site in a Human Hsp70 Chaperone BiP. *Structure* **23**, 2191–2203 (2015). 26655470 10.1016/j.str.2015.10.012 PMC4680848

[49] Hetz, C., Zhang, K. & Kaufman, R. J. Mechanisms, regulation and functions of the unfolded protein response. *Nat. Rev. Mol. Cell Biol.* **21**, 421–438 (2020). 32457508 10.1038/s41580-020-0250-z PMC8867924

[50] Walter, P. & Ron, D. The unfolded protein response: from stress pathway to homeostatic regulation. *Science* **334**, 1081–1086 (2011). 22116877 10.1126/science.1209038

[51] Mori, K. Tripartite management of unfolded proteins in the endoplasmic reticulum. *Cell* **101**, 451–454 (2000). 10850487 10.1016/s0092-8674(00)80855-7

[52] Hetz, C. The unfolded protein response: controlling cell fate decisions under ER stress and beyond. *Nat. Rev. Mol. Cell Biol.* **13**, 89–102 (2012). 22251901 10.1038/nrm3270

[53] Aughey, G. N. & Liu, J. L. Metabolic regulation via enzyme filamentation. *Crit. Rev. Biochem Mol. Biol.* **51**, 282–293 (2015). 27098510 10.3109/10409238.2016.1172555 PMC4915340

[54] O’Connell, J. D., Zhao, A., Ellington, A. D. & Marcotte, E. M. Dynamic reorganization of metabolic enzymes into intracellular bodies. *Annu Rev. Cell Dev. Biol.* **28**, 89–111 (2012). 23057741 10.1146/annurev-cellbio-101011-155841 PMC4089986

[55] Piazza, I. et al. A Map of Protein-Metabolite Interactions Reveals Principles of Chemical Communication. *Cell* **172**, 358–372.e323 (2018). 29307493 10.1016/j.cell.2017.12.006

[56] Vogel, M., Mayer, M. P. & Bukau, B. Allosteric regulation of Hsp70 chaperones involves a conserved interdomain linker. *J. Biol. Chem.* **281**, 38705–38711 (2006). 17052976 10.1074/jbc.M609020200

[57] Swain, J. F. et al. Hsp70 chaperone ligands control domain association via an allosteric mechanism mediated by the interdomain linker. *Mol. Cell* **26**, 27–39 (2007). 17434124 10.1016/j.molcel.2007.02.020 PMC1894942

[58] Amin-Wetzel, N. et al. A J-Protein Co-chaperone Recruits BiP to Monomerize IRE1 and Repress the Unfolded Protein Response. *Cell* **171**, 1625–1637.e1613 (2017). 29198525 10.1016/j.cell.2017.10.040 PMC5733394

[59] Wang, Y. et al. A nuclease that mediates cell death induced by DNA damage and poly(ADP-ribose) polymerase-1. *Science* **354**, aad6872 (2016). 27846469 10.1126/science.aad6872 PMC5134926

[60] Hu, S. et al. Profiling the human protein-DNA interactome reveals ERK2 as a transcriptional repressor of interferon signaling. *Cell* **139**, 610–622 (2009). 19879846 10.1016/j.cell.2009.08.037 PMC2774939

[61] Manjur, A. et al. IRF2BP2 modulates the crosstalk between glucocorticoid and TNF signaling. *J. Steroid Biochem Mol. Biol.* **192**, 105382 (2019). 31145973 10.1016/j.jsbmb.2019.105382

[62] Lempiäinen, J. K. et al. Agonist-specific Protein Interactomes of Glucocorticoid and Androgen Receptor as Revealed by Proximity Mapping. *Mol. Cell Proteom.* **16**, 1462–1474 (2017). 10.1074/mcp.M117.067488 PMC5546198 28611094

[63] Dorand, R. D. et al. Cdk5 disruption attenuates tumor PD-L1 expression and promotes antitumor immunity. *Science* **353**, 399–403 (2016). 27463676 10.1126/science.aae0477 PMC5051664

[64] Cha-Molstad, H. et al. Amino-terminal arginylation targets endoplasmic reticulum chaperone BiP for autophagy through p62 binding. *Nat. Cell Biol.* **17**, 917–929 (2015). 26075355 10.1038/ncb3177 PMC4490096

[65] Shim, S. M. et al. The endoplasmic reticulum-residing chaperone BiP is short-lived and metabolized through N-terminal arginylation. *Sci. Signal* **11**, eaan0630 (2018). 29295953 10.1126/scisignal.aan0630

[66] Ramalho-Oliveira, R., Oliveira-Vieira, B. & Viola, J. P. B. IRF2BP2: A new player in the regulation of cell homeostasis. *J. Leukoc. Biol.* **106**, 717–723 (2019). 31022319 10.1002/JLB.MR1218-507R

[67] Teng, A. C. et al. Identification of a phosphorylation-dependent nuclear localization motif in interferon regulatory factor 2 binding protein 2. *PLoS One* **6**, e24100 (2011). 21887377 10.1371/journal.pone.0024100 PMC3162591

[68] Hu, X. et al. Ubiquitin-fold modifier 1 inhibits apoptosis by suppressing the endoplasmic reticulum stress response in Raw264.7 cells. *Int J. Mol. Med.* **33**, 1539–1546 (2014). 24714921 10.3892/ijmm.2014.1728

[69] Lin, W. W., Chang, S. H. & Wu, M. L. Lipoxygenase metabolites as mediators of UTP-induced intracellular acidification in mouse RAW 264.7 macrophages. *Mol. Pharm.* **53**, 313–321 (1998). 10.1124/mol.53.2.313 9463490

[70] Gething, M. J. Role and regulation of the ER chaperone BiP. *Semin Cell Dev. Biol.* **10**, 465–472 (1999). 10597629 10.1006/scdb.1999.0318

[71] Liao, Y. et al. Targeting GRP78-dependent AR-V7 protein degradation overcomes castration-resistance in prostate cancer therapy. *Theranostics* **10**, 3366–3381 (2020). 32206096 10.7150/thno.41849 PMC7069092

[72] Sun, S. et al. IRE1α is an endogenous substrate of endoplasmic-reticulum-associated degradation. *Nat. Cell Biol.* **17**, 1546–1555 (2015). 26551274 10.1038/ncb3266 PMC4670240

[73] Teng, A. C. et al. IRF2BP2 is a skeletal and cardiac muscle-enriched ischemia-inducible activator of VEGFA expression. *Faseb j.* **24**, 4825–4834 (2010). 20702774 10.1096/fj.10-167049

[74] Chen, S. et al. Immunoproteasome dysfunction augments alternative polarization of alveolar macrophages. *Cell Death Differ.* **23**, 1026–1037 (2016). 26990663 10.1038/cdd.2016.3 PMC4987736

[75] Doyle, A. et al. Toll-like receptor 4 mediates lipopolysaccharide-induced muscle catabolism via coordinate activation of ubiquitin-proteasome and autophagy-lysosome pathways. *Faseb j.* **25**, 99–110 (2011). 20826541 10.1096/fj.10-164152 PMC3005430

[76] Bassnett, S. Intracellular pH regulation in the embryonic chicken lens epithelium. *J. Physiol.* **431**, 445–464 (1990). 1966051 10.1113/jphysiol.1990.sp018339 PMC1181783

[77] Smith, J. B. & Rozengurt, E. Serum stimulates the Na+,K+ pump in quiescent fibroblasts by increasing Na+ entry. *Proc. Natl Acad. Sci. USA* **75**, 5560–5564 (1978). 82969 10.1073/pnas.75.11.5560 PMC393006

[78] Hu, M. et al. Parkinson’s disease-risk protein TMEM175 is a proton-activated proton channel in lysosomes. *Cell* **185**, 2292–2308.e2220 (2022). 35750034 10.1016/j.cell.2022.05.021 PMC9236176

[79] Ludwig, M. G. et al. Proton-sensing G-protein-coupled receptors. *Nature* **425**, 93–98 (2003). 12955148 10.1038/nature01905

[80] Choi, C. H. et al. pH sensing by FAK-His58 regulates focal adhesion remodeling. *J. Cell Biol.* **202**, 849–859 (2013). 24043700 10.1083/jcb.201302131 PMC3776353

[81] Jasti, J., Furukawa, H., Gonzales, E. B. & Gouaux, E. Structure of acid-sensing ion channel 1 at 1.9 A resolution and low pH. *Nature* **449**, 316–323 (2007). 17882215 10.1038/nature06163

[82] Ramaswamy, S. S., MacLean, D. M., Gorfe, A. A. & Jayaraman, V. Proton-mediated conformational changes in an acid-sensing ion channel. *J. Biol. Chem.* **288**, 35896–35903 (2013). 24196950 10.1074/jbc.M113.478982 PMC3861639

[83] Scott, N. A. et al. Antibiotics induce sustained dysregulation of intestinal T cell immunity by perturbing macrophage homeostasis. *Sci. Transl. Med.* **10**, eaao4755 (2018). 30355800 10.1126/scitranslmed.aao4755 PMC6548564

[84] Juillerat, P. et al. Drugs that inhibit gastric acid secretion may alter the course of inflammatory bowel disease. *Aliment Pharm. Ther.* **36**, 239–247 (2012). 10.1111/j.1365-2036.2012.05173.x 22670722

[85] Lu, T. X. et al. The influence of proton pump inhibitor therapy on the outcome of infliximab therapy in inflammatory bowel disease: a patient-level meta-analysis of randomised controlled studies. *Gut* **70**, 2076–2084 (2021). 33334900 10.1136/gutjnl-2020-321609

[86] Koçak, E. et al. NaOH-induced Crohn’s colitis in rats: a novel experimental model. *Dig. Dis. Sci.* **56**, 2833–2837 (2011). 21503680 10.1007/s10620-011-1697-8

[87] Umbach, A. T. et al. Intestinal Na+ loss and volume depletion in JAK3-deficient mice. *Kidney Blood Press Res.* **37**, 514–520 (2013). 24281140 10.1159/000355731

[88] Jiang, Y. & Shen, Q. IRF2BP2 prevents ox-LDL-induced inflammation and EMT in endothelial cells via regulation of KLF2. *Exp. Ther. Med.* **21**, 481 (2021). 33767776 10.3892/etm.2021.9912 PMC7976449

[89] Cruz, S. A. et al. Loss of IRF2BP2 in Microglia Increases Inflammation and Functional Deficits after Focal Ischemic Brain Injury. *Front Cell Neurosci.* **11**, 201 (2017). 28769762 10.3389/fncel.2017.00201 PMC5515910

[90] Childs, K. S. & Goodbourn, S. Identification of novel co-repressor molecules for Interferon Regulatory Factor-2. *Nucleic Acids Res.* **31**, 3016–3026 (2003). 12799427 10.1093/nar/gkg431 PMC162335

[91] Cuesta, N., Salkowski, C. A., Thomas, K. E. & Vogel, S. N. Regulation of lipopolysaccharide sensitivity by IFN regulatory factor-2. *J. Immunol.* **170**, 5739–5747 (2003). 12759457 10.4049/jimmunol.170.11.5739

[92] Simmen, H. P. & Blaser, J. Analysis of pH and pO2 in abscesses, peritoneal fluid, and drainage fluid in the presence or absence of bacterial infection during and after abdominal surgery. *Am. J. Surg.* **166**, 24–27 (1993). 8328625 10.1016/s0002-9610(05)80576-8

[93] Coakley, R. J., Taggart, C., McElvaney, N. G. & O’Neill, S. J. Cytosolic pH and the inflammatory microenvironment modulate cell death in human neutrophils after phagocytosis. *Blood* **100**, 3383–3391 (2002). 12384441 10.1182/blood.V100.9.3383

[94] Rotstein, O. D., Fiegel, V. D., Simmons, R. L. & Knighton, D. R. The deleterious effect of reduced pH and hypoxia on neutrophil migration in vitro. *J. Surg. Res.* **45**, 298–303 (1988). 3411954 10.1016/0022-4804(88)90079-0

[95] Grinstein, S., Swallow, C. J. & Rotstein, O. D. Regulation of cytoplasmic pH in phagocytic cell function and dysfunction. *Clin. Biochem* **24**, 241–247 (1991). 1651820 10.1016/0009-9120(91)80014-t

[96] Rotstein, O. D., Nasmith, P. E. & Grinstein, S. The Bacteroides by-product succinic acid inhibits neutrophil respiratory burst by reducing intracellular pH. *Infect. Immun.* **55**, 864–870 (1987). 3030935 10.1128/iai.55.4.864-870.1987 PMC260430

[97] Lardner, A. The effects of extracellular pH on immune function. *J. Leukoc. Biol.* **69**, 522–530 (2001). 11310837

[98] Yuli, I. & Oplatka, A. Cytosolic acidification as an early transductory signal of human neutrophil chemotaxis. *Science* **235**, 340–342 (1987). 3798116 10.1126/science.3798116

[99] Chae, B. J., Lee, K. S., Hwang, I. & Yu, J. W. Extracellular Acidification Augments NLRP3-Mediated Inflammasome Signaling in Macrophages. *Immune Netw.* **23**, e23 (2023). 37416933 10.4110/in.2023.23.e23 PMC10320421

[100] Rajamäki, K. et al. Extracellular acidosis is a novel danger signal alerting innate immunity via the NLRP3 inflammasome. *J. Biol. Chem.* **288**, 13410–13419 (2013). 23530046 10.1074/jbc.M112.426254 PMC3650379

[101] Wang, Y. et al. The NOTCH1-dependent HIF1α/VGLL4/IRF2BP2 oxygen sensing pathway triggers erythropoiesis terminal differentiation. *Redox Biol.* **28**, 101313 (2020). 31539803 10.1016/j.redox.2019.101313 PMC6812007

[102] Yang, Z. et al. Exercise ameliorates high-fat diet-induced insulin resistance accompanied by changes in protein levels of hepatic ATF3-related signaling in rats. *Physiol. Behav.* **249**, 113766 (2022). 35240124 10.1016/j.physbeh.2022.113766

[103] Jonásová, L. et al. Hydroxyapatite formation on alkali-treated titanium with different content of Na+ in the surface layer. *Biomaterials* **23**, 3095–3101 (2002). 12102180 10.1016/s0142-9612(02)00043-1

[104] van Solingen, C. et al. Long noncoding RNA CHROMR regulates antiviral immunity in humans. *Proc. Natl Acad. Sci. USA* **119**, e2210321119 (2022). 36001732 10.1073/pnas.2210321119 PMC9477407

[105] Okda, F. A., Perry, S. S., Webby, R. J. & Russell, C. J. Interplay between H1N1 influenza a virus infection, extracellular and intracellular respiratory tract pH, and host responses in a mouse model. *PLoS One* **16**, e0251473 (2021). 33979408 10.1371/journal.pone.0251473 PMC8115840

[106] Ziegelstein, R. C., Cheng, L. & Capogrossi, M. C. Flow-dependent cytosolic acidification of vascular endothelial cells. *Science* **258**, 656–659 (1992). 1329207 10.1126/science.1329207

[107] Bright, C. M. & Ellis, D. Hypoxia-induced intracellular acidification in isolated sheep heart Purkinje fibres and the effects of temperature. *J. Mol. Cell Cardiol.* **26**, 463–469 (1994). 8072004 10.1006/jmcc.1994.1057

[108] Panikkanvalappil, S. R. et al. Hyperoxia Induces Intracellular Acidification in Neonatal Mouse Lung Fibroblasts: Real-Time Investigation Using Plasmonically Enhanced Raman Spectroscopy. *J. Am. Chem. Soc.* **138**, 3779–3788 (2016). 26938952 10.1021/jacs.5b13177

[109] Dechant, R., Saad, S., Ibáñez, A. J. & Peter, M. Cytosolic pH regulates cell growth through distinct GTPases, Arf1 and Gtr1, to promote Ras/PKA and TORC1 activity. *Mol. Cell* **55**, 409–421 (2014). 25002144 10.1016/j.molcel.2014.06.002

[110] Dechant, R. et al. Cytosolic pH is a second messenger for glucose and regulates the PKA pathway through V-ATPase. *Embo j.* **29**, 2515–2526 (2010). 20581803 10.1038/emboj.2010.138 PMC2928683

[111] Pastor, T. P., Peixoto, B. C. & Viola, J. P. B. The Transcriptional Co-factor IRF2BP2: A New Player in Tumor Development and Microenvironment. *Front Cell Dev. Biol.* **9**, 655307 (2021). 33996817 10.3389/fcell.2021.655307 PMC8116537

[112] Wu, A. et al. Loss of VGLL4 suppresses tumor PD-L1 expression and immune evasion. *Embo j.* **38**, e99506 (2019). 30396996 10.15252/embj.201899506 PMC6589543

[113] Feng, X. et al. The Tumor Suppressor Interferon Regulatory Factor 2 Binding Protein 2 Regulates Hippo Pathway in Liver Cancer by a Feedback Loop in Mice. *Hepatology* **71**, 1988–2004 (2020). 31538665 10.1002/hep.30961

[114] Koeppel, M. et al. The novel p53 target gene IRF2BP2 participates in cell survival during the p53 stress response. *Nucleic Acids Res* **37**, 322–335 (2009). 19042971 10.1093/nar/gkn940 PMC2632907

[115] Fang, J. et al. Control of Pathological Cardiac Hypertrophy by Transcriptional Corepressor IRF2BP2 (Interferon Regulatory Factor-2 Binding Protein 2). *Hypertension* **70**, 515–523 (2017). 28716987 10.1161/HYPERTENSIONAHA.116.08728

[116] Hellwig, N. et al. TRPV1 acts as proton channel to induce acidification in nociceptive neurons. *J. Biol. Chem.* **279**, 34553–34561 (2004). 15173182 10.1074/jbc.M402966200

[117] Young, B. P. et al. Phosphatidic acid is a pH biosensor that links membrane biogenesis to metabolism. *Science* **329**, 1085–1088 (2010). 20798321 10.1126/science.1191026

[118] Schroeder, M. A. et al. Measuring intracellular pH in the heart using hyperpolarized carbon dioxide and bicarbonate: a 13C and 31P magnetic resonance spectroscopy study. *Cardiovasc Res* **86**, 82–91 (2010). 20008827 10.1093/cvr/cvp396 PMC2836261

[119] Webb, B. A., Chimenti, M., Jacobson, M. P. & Barber, D. L. Dysregulated pH: a perfect storm for cancer progression. *Nat. Rev. Cancer* **11**, 671–677 (2011). 21833026 10.1038/nrc3110

[120] Kim, I., Xu, W. & Reed, J. C. Cell death and endoplasmic reticulum stress: disease relevance and therapeutic opportunities. *Nat. Rev. Drug Discov.* **7**, 1013–1030 (2008). 19043451 10.1038/nrd2755

[121] Janssens, S., Pulendran, B. & Lambrecht, B. N. Emerging functions of the unfolded protein response in immunity. *Nat. Immunol.* **15**, 910–919 (2014). 25232821 10.1038/ni.2991 PMC4388558

[122] Keestra-Gounder, A. M. et al. NOD1 and NOD2 signalling links ER stress with inflammation. *Nature* **532**, 394–397 (2016). 27007849 10.1038/nature17631 PMC4869892

[123] Martinon, F., Chen, X., Lee, A. H. & Glimcher, L. H. TLR activation of the transcription factor XBP1 regulates innate immune responses in macrophages. *Nat. Immunol.* **11**, 411–418 (2010). 20351694 10.1038/ni.1857 PMC3113706

[124] Márquez, S. et al. Endoplasmic Reticulum Stress Sensor IRE1α Enhances IL-23 Expression by Human Dendritic Cells. *Front Immunol.* **8**, 639 (2017). 28674530 10.3389/fimmu.2017.00639 PMC5475432

[125] Yoo, W. et al. HIF-1α expression as a protective strategy of HepG2 cells against fatty acid-induced toxicity. *J. Cell Biochem* **115**, 1147–1158 (2014). 24402912 10.1002/jcb.24757

[126] Liu, X. et al. HIF-1-regulated expression of calreticulin promotes breast tumorigenesis and progression through Wnt/β-catenin pathway activation. *Proc. Natl Acad. Sci. USA* **118**, e2121952119 (2021). 10.1073/pnas.2109144118 PMC8612225 34706936

[127] Michalak, M. et al. Calreticulin, a multi-process calcium-buffering chaperone of the endoplasmic reticulum. *Biochem J.* **417**, 651–666 (2009). 19133842 10.1042/BJ20081847

[128] Matlack, K. E., Misselwitz, B., Plath, K. & Rapoport, T. A. BiP acts as a molecular ratchet during posttranslational transport of prepro-alpha factor across the ER membrane. *Cell* **97**, 553–564 (1999). 10367885 10.1016/s0092-8674(00)80767-9

[129] Hamman, B. D., Hendershot, L. M. & Johnson, A. E. BiP maintains the permeability barrier of the ER membrane by sealing the lumenal end of the translocon pore before and early in translocation. *Cell* **92**, 747–758 (1998). 9529251 10.1016/s0092-8674(00)81403-8

[130] Lyman, S. K. & Schekman, R. Binding of secretory precursor polypeptides to a translocon subcomplex is regulated by BiP. *Cell* **88**, 85–96 (1997). 9019409 10.1016/s0092-8674(00)81861-9

[131] Sanders, S. L. et al. Sec61p and BiP directly facilitate polypeptide translocation into the ER. *Cell* **69**, 353–365 (1992). 1568250 10.1016/0092-8674(92)90415-9

[132] Flynn, G. C., Pohl, J., Flocco, M. T. & Rothman, J. E. Peptide-binding specificity of the molecular chaperone BiP. *Nature* **353**, 726–730 (1991). 1834945 10.1038/353726a0

[133] Kassenbrock, C. K., Garcia, P. D., Walter, P. & Kelly, R. B. Heavy-chain binding protein recognizes aberrant polypeptides translocated in vitro. *Nature* **333**, 90–93 (1988). 3129663 10.1038/333090a0

[134] Miharada, K. et al. Cripto regulates hematopoietic stem cells as a hypoxic-niche-related factor through cell surface receptor GRP78. *Cell Stem Cell* **9**, 330–344 (2011). 21982233 10.1016/j.stem.2011.07.016

[135] Arap, M. A. et al. Cell surface expression of the stress response chaperone GRP78 enables tumor targeting by circulating ligands. *Cancer Cell* **6**, 275–284 (2004). 15380518 10.1016/j.ccr.2004.08.018

[136] Gao, X. et al. Metabolite analysis of human fecal water by gas chromatography/mass spectrometry with ethyl chloroformate derivatization. *Anal. Biochem* **393**, 163–175 (2009). 19573517 10.1016/j.ab.2009.06.036

[137] Gao, X., Pujos-Guillot, E. & Sébédio, J. L. Development of a quantitative metabolomic approach to study clinical human fecal water metabolome based on trimethylsilylation derivatization and GC/MS analysis. *Anal. Chem.* **82**, 6447–6456 (2010). 20669995 10.1021/ac1006552

[138] De Filippis, F. et al. High-level adherence to a Mediterranean diet beneficially impacts the gut microbiota and associated metabolome. *Gut* **65**, 1812–1821 (2016). 26416813 10.1136/gutjnl-2015-309957

[139] Ji, C., Mei, Y. & Zhang, J. Z. Developing polarized protein-specific charges for protein dynamics: MD free energy calculation of pKa shifts for Asp26/Asp20 in thioredoxin. *Biophys. J.* **95**, 1080–1088 (2008). 18645195 10.1529/biophysj.108.131110 PMC2479593

[140] Casey, J. R., Grinstein, S. & Orlowski, J. Sensors and regulators of intracellular pH. *Nat. Rev. Mol. Cell Biol.* **11**, 50–61 (2010). 19997129 10.1038/nrm2820

[141] Flinck, M. et al. The acid-base transport proteins NHE1 and NBCn1 regulate cell cycle progression in human breast cancer cells. *Cell Cycle* **17**, 1056–1067 (2018). 29895196 10.1080/15384101.2018.1464850 PMC6110587

[142] Steinbach, N. et al. PTEN interacts with the transcription machinery on chromatin and regulates RNA polymerase II-mediated transcription. *Nucleic Acids Res.* **47**, 5573–5586 (2019). 31169889 10.1093/nar/gkz272 PMC6582409

[143] Cristea, I. M., Williams, R., Chait, B. T. & Rout, M. P. Fluorescent proteins as proteomic probes. *Mol. Cell Proteom.* **4**, 1933–1941 (2005). 10.1074/mcp.M500227-MCP200 16155292

[144] Xiang, W. et al. Monoacylglycerol lipase regulates cannabinoid receptor 2-dependent macrophage activation and cancer progression. *Nat. Commun.* **9**, 2574 (2018). 29968710 10.1038/s41467-018-04999-8 PMC6030061

[145] Khachigian, L. M. Collagen antibody-induced arthritis. *Nat. Protoc.* **1**, 2512–2516 (2006). 17406499 10.1038/nprot.2006.393

[146] Zhao, W. et al. A subunit of V-ATPases, ATP6V1B2, underlies the pathology of intellectual disability. *EBioMedicine* **45**, 408–421 (2019). 31257146 10.1016/j.ebiom.2019.06.035 PMC6642280

[147] Shen, S. M. et al. Nuclear PTEN safeguards pre-mRNA splicing to link Golgi apparatus for its tumor suppressive role. *Nat. Commun.* **9**, 2392 (2018). 29921876 10.1038/s41467-018-04760-1 PMC6008332

[148] Swindell, W. R. et al. ALS blood expression profiling identifies new biomarkers, patient subgroups, and evidence for neutrophilia and hypoxia. *J. Transl. Med.* **17**, 170 (2019). 31118040 10.1186/s12967-019-1909-0 PMC6530130

[149] Yan, M. et al. Metabolomics profiling of metformin-mediated metabolic reprogramming bypassing AMPKα. *Metabolism* **91**, 18–29 (2019). 30468782 10.1016/j.metabol.2018.11.010

[150] Zeng, J. et al. Metabolomics study of hepatocellular carcinoma: discovery and validation of serum potential biomarkers by using capillary electrophoresis-mass spectrometry. *J. Proteome Res.* **13**, 3420–3431 (2014). 24853826 10.1021/pr500390y

[151] Millard, P. et al. IsoCor: isotope correction for high-resolution MS labeling experiments. *Bioinformatics* **35**, 4484–4487 (2019). 30903185 10.1093/bioinformatics/btz209

