# Supplementary material for: Malate initiates a proton-sensing pathway essential for pH regulation of inflammation
Source: Signal Transduct Target Ther. 2024 Dec 30;9:367. doi: 10.1038/s41392-024-02076-9 (PMC11683149; doi:10.1038/s41392-024-02076-9)
Supplement: Supplementary file 1 — Supplementary Materials [file 41392_2024_2076_MOESM1_ESM.docx]

Supplementary Materials for

**Malate initiates a proton-sensing pathway essential for pH regulation of inflammation**

Yu-jia-nan Chen^1,2,3,4,6,#,^*, Rong-chen Shi^2,6,#^, Yuan-cai Xiang^2,6,11,#^, Li Fan^1,4,#^, Hong Tang^1^, Gang He^1^, Mei Zhou^1^, Xin-zhe Feng^3^, Jin-dong Tan^1,10^, Pan Huang^1^, Xiao Ye^1^, Kun Zhao^2,6^, Wen-yu Fu^3,7^, Liu-li Li^2^, Xu-ting Bian^1^, Huan Chen^8^, Feng Wang^1^, Teng Wang^2,6^, Chen-ke Zhang^1^, Bing-hua Zhou^1^, Wan Chen^1^, Tao-tao Liang^1^, Jing-tong Lv^1^, Xia Kang^1,2,6^, You-xing Shi^1^, Ellen Kim^3^, Yin-hua Qin^9^, Aubryanna Hettinghouse^3^, Kai-di Wang^3,12^, Xiang-li Zhao^3,7^, Ming-yu Yang^1^, Yu-zhen Tang^1^, Hai-long Piao^8^, Lin Guo^1,^*, Chuan-ju Liu^3,7^^,^*, Hong-ming Miao^2,5,^*, Kang-lai Tang^1,^*

Correspondence to: chenyujiananjade@hotmail.com, guolin6212@163.com, chuan-ju.liu@yale.edu, hongmingmiao@sina.com or tangkanglai@hotmail.com

**This PDF file includes:**

Supplementary Figures 1-10


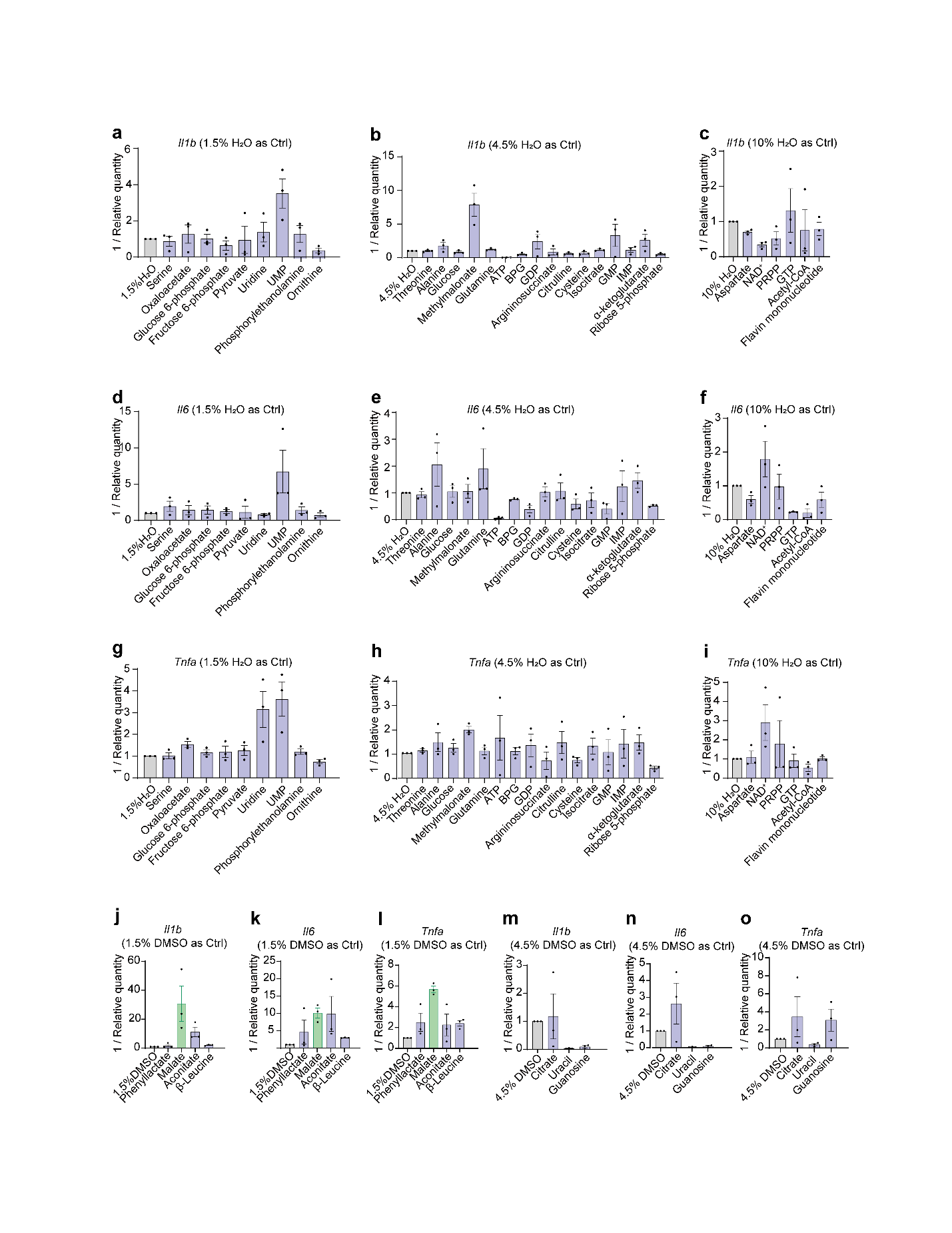


Supplementary Figure. 1. TCA-cycle-related metabolites screening in LPS-induced macrophage activation, related to Figure 1

(a-o) BMDMs were incubated with each metabolite or the respective solubility-matched vehicle including 1.5% volume H_2_O (a, d, g), 4.5% volume H_2_O (b, e, h), 10% volume H_2_O (c, f, i), 1.5% volume DMSO (j - l), or 4.5% volume DMSO (m - o), under LPS stimulation for 24 h. *Il1b*, *Il6*, and *Tnfa* mRNA expressions were measured. n = 3. Data are shown as mean ± SEM.


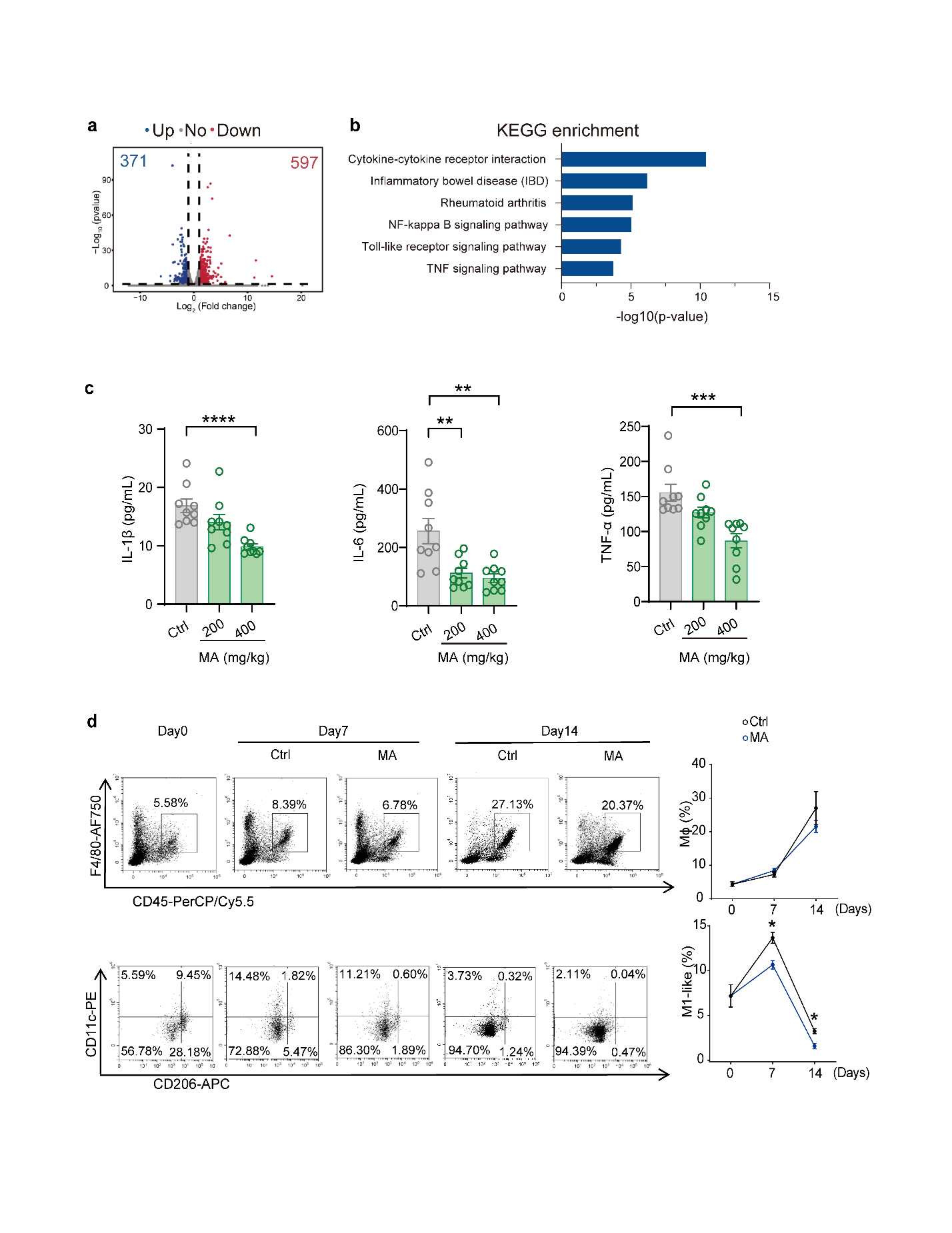


**Supplementary Figure. 2. L-malate decreases severity of collagen antibody induced arthritis, related to Figure 1**

(a) Volcano plot showing differentially expressed genes (DEGs) in BMDMs treated with LPS plus L-malate compared to LPS for 12 h. Black lines indicate thresholds for significant DEGs [p-value < 0.05, abs (log2 fold change) > 1].

(b) KEGG pathway enrichment on DEGs.

(c) The levels of IL-1β, IL-6, and TNF-α in serum of the CAIA mice treated with different dosages of L-malate (0, 200 and 400 mg/kg, n = 9 per group).

(d) Macrophage infiltration in paw of CAIA model. Macrophages (CD45^+^F4/80^+^CD3^-^CD45R^-^) and M1-like cells (CD45^+^F4/80^+^CD11c^+^CD206^-^CD3^-^CD45R^-^) were dynamically calculated by flow cytometry.

n ≥ 3. Data are shown as mean ± SEM. *p<0.05; **p <0.01; ***p <0.001; ****p <0.0001 (unpaired Student’s t test).


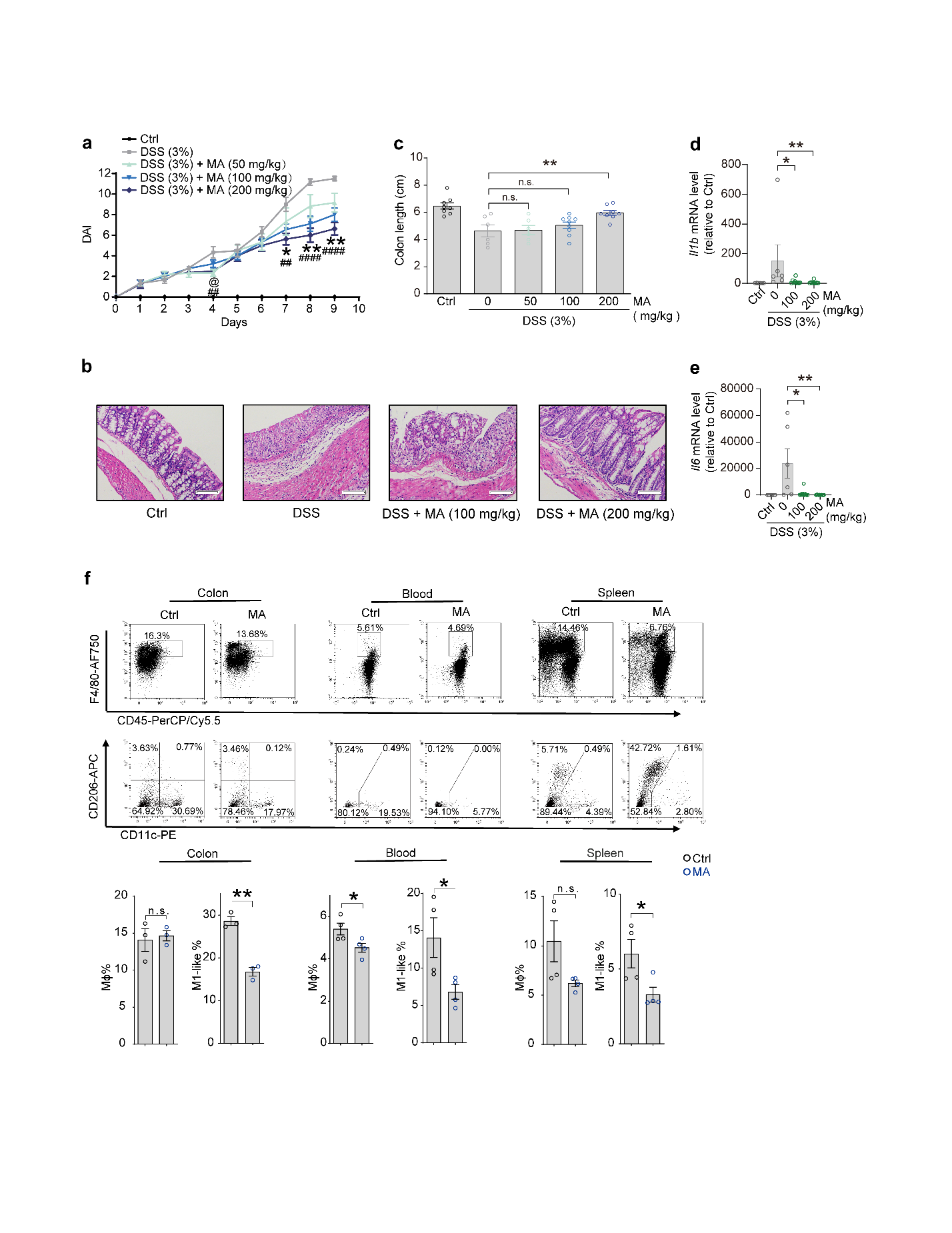


**Supplementary Figure. 3. L-malate decreases severity of DSS induced colitis, related to Figure 1**

(a) Disease Activity Index for PBS and L-malate (50 mg/kg, 100 mg/kg, 200 mg/kg) treated mice in 3% DSS-induced colitis model (n = 6 - 9 per group).

(b) Representative histological photographs of H&E-stained colon sections. Scale bar, 100 μm.

(c) Colon length of the mice as described above in a.

(d, e) *Il1b* (d) and *Il6* (e) expressions measured by qPCR in colons of the mice as described above in a.

(f) Macrophage infiltration in colon, blood and spleen of the mice as described above in A. Macrophages (CD45^+^F4/80^+^CD3^-^ CD45R^-^) and M1-like cells (CD45^+^F4/80^+^CD11c^+^CD206^-^CD3^-^CD45R^-^) were calculated by flow cytometry.

Data are shown as mean ± SEM. In (b), @p <0.05 (DSS 3% + 50 mg/kg L-malate compared to DSS 3% + PBS); *p < 0.05, **p < 0.01 (DSS 3% + PBS compared to DSS 3% + 100 mg/kg L-malate); ##p < 0.01, ####p < 0.0001 (DSS 3% + 200 mg/kg L-malate compared to DSS 3% + PBS). In (c, d, e, f), *p < 0.05, **p < 0.01 (two-tailed t-tests, Mann-whitmey U test or one-way ANOVA).


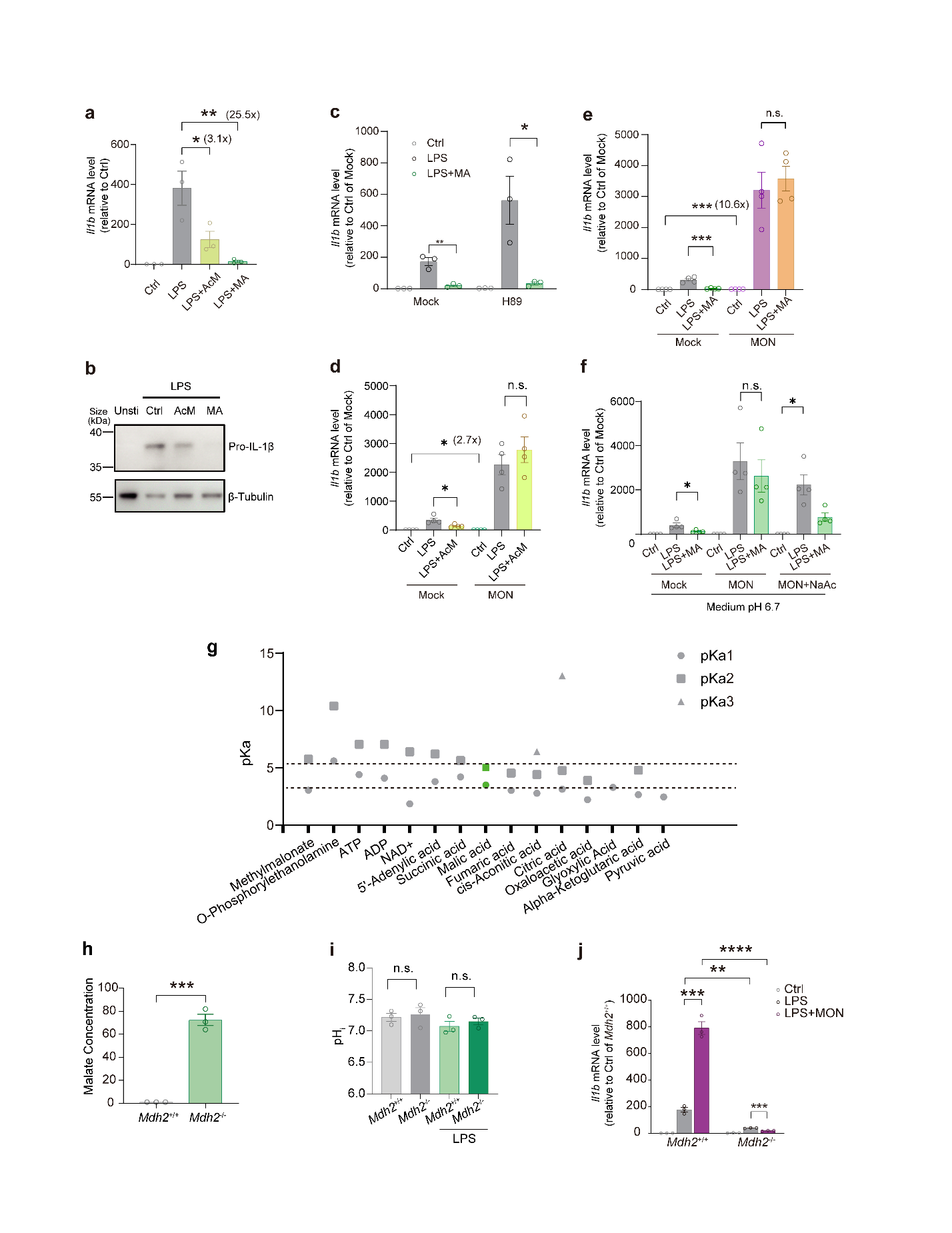


**Supplementary Figure. 4. The anti-inflammatory regulation of L-malate addition is mediated by both pH_i_ acidification and L-malate compounds, related to Figure 1**

(a, b) *Il1b* mRNA levels measured by qPCR (A) pro-IL-1β protein levels visualized by Western blot (b) in BMDMs treated with LPS for 16h treated with acidized medium (AcM) (pH 6.7) or L-malate (6 mM, pH 6.7) under stimulation of LPS (16 h)

(c) The *Il1b* mRNA levels of LPS-stimulated BMDMs treated with or without L-malate and H89 for 24 h.

(d) The *Il1b* expression in LPS-stimulated and unstimulated BMDMs treated with acidized medium (AcM) (pH 6.7) in the presence of monensin (1 μM) for 16 h.

(e) The *Il1b* expression in LPS-stimulated and unstimulated BMDMs treated with L-malate (6 mM, pH 6.7) in the presence of monensin (1 μM) for 24 h.

(f) The *Il1b* expression in LPS-stimulated BMDMs (medium pH 6.7) treated with L-malate (6 mM) in the presence of monensin or monensin plus sodium acetate (NaAc, 20 mM) for 16 h.

(g) pKa of acidic candidates of metabolite screening in LPS-induced activation of macrophages (related to Supplementary Figure 1). The value of L-malate is colored by green.

(h) L-malate levels in *Mdh2*^-/-^ and WT Raw264.7 cell lines measured by LC-MS/MS.

(i) pH_i_ of *Mdh2*^-/-^ and *Mdh2*^+/+^ Raw264.7 cell lines stimulated by LPS for 3 h.

(j) The *Il1b* mRNA levels in WT or *Mdh2*^-/-^ BMDMs treated with or without monensin (5 μM) and stimulated with LPS (24 h).

n ≥ 3. Data are shown as mean ± SEM. *p<0.05; **p <0.01; ***p <0.001; ****p <0.0001 (unpaired Student’s t test, one-way ANOVA).


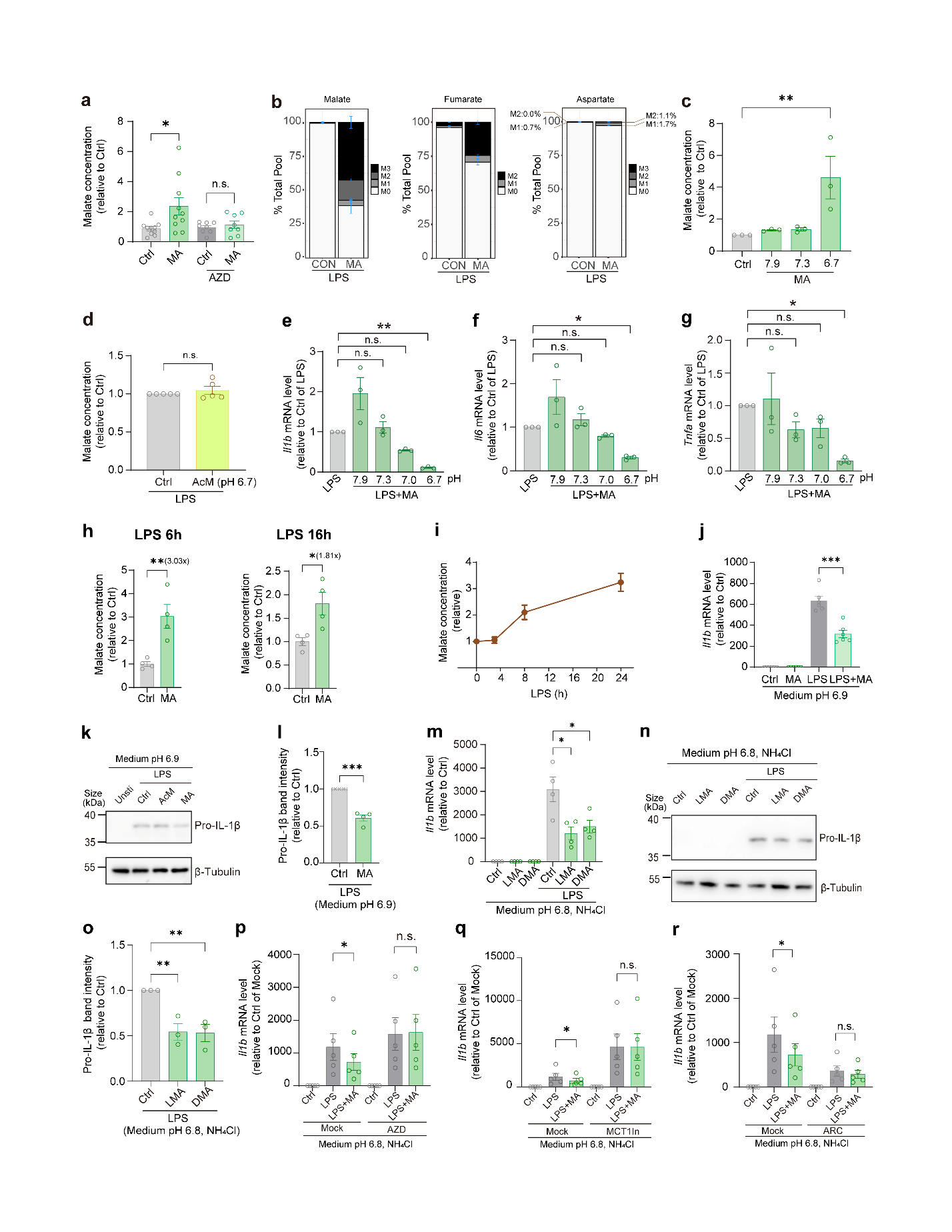


**Supplementary Figure. 5.** **L-malate acts as an intracellular anti-inflammatory metabolite with physiological relevance, related to Figure 1**

(a) L-malate level of PBMCs from C57BL/6J mice at 6 h after receiving intragastric administration of L-malate (200 mg/kg) and intraperitoneal injection of saline or AZD3965 (70 mg/kg, added 3 h before L-malate treatment) (n = 8 - 10 per group).

(b) Mass isotopologue analysis of L-malate-d3 labeling experiments tracking intracellular metabolism of extracellularly applied L-malate in BMDMs (controlled medium pH 6.9) stimulated by LPS for 8 h (n = 2). Mx (x = 0 - 3) represents the proportion of certain metabolite containing “x” deuterium(s) in its total pool.

(c) Intracellular L-malate levels in BMDMs treated by L-malate (6 mM, 6 h) at different pH (n = 3).

(d) Intracellular L-malate abundance in LPS-stimulated BMDMs treated with or without acidized medium (pH 6.7, 24 h) (n = 5).

(e-g) *Il1b* (e), Il6 (f), *Tnfa* (g) mRNA levels measured by qPCR in LPS-stimulated BMDMs treated with L-malate at different pH (n = 3).

(h) Intracellular levels of L-malate analyzed by LC–MS/MS relative to vehicle in LPS-stimulated BMDMs treated with or without L-malate (6 mM, pH 6.7) for 6 h and 16 h (n = 4).

(i) Intracellular levels of L-malate analyzed by LC–MS/MS relative to vehicle in BMDMs stimulated by LPS for 0, 3, 8 and 24 h (n = 3).

(j) The *Il1b* mRNA expression in LPS-stimulated BMDMs (medium pH 6.9) treated with or without L-malate (1.5 mM) for 16 h (n = 6).

(k, l) The pro-IL-1β protein levels in LPS-stimulated BMDMs (medium pH 6.9) treated with or without L-malate (1.5 mM) for 16 h (n = 4).

(m-o) The *Il1b* mRNA expression (N) in pro-IL-1β protein levels (N, O) in LPS-stimulated BMDMs (medium pH 6.8) treated with L-malate (0.5 mM) and D-malate (0.5 mM) in the presence of NH_4_Cl (20 mM) for 16 h (n = 3 - 4).

(p-r) The *Il1b* expression in LPS-stimulated (16 h) BMDMs (medium pH 6.8) treated with L-malate (0.5 mM, 16 h) and D-malate (0.5 mM, 16 h) in the presence of NH_4_Cl (20 mM) plus AZD3965 (AZD) (r) or MCT1-In-2 (MCT1In) (q) or AR-C155858 (ARC) (r) for 17 h (added 1 h before the treatment of LPS and L-malate) (n = 5)

Data are shown as mean ± SEM. *p<0.05; **p <0.01; ***p <0.001; ****p <0.0001 (unpaired Student’s t test, one-way ANOVA).


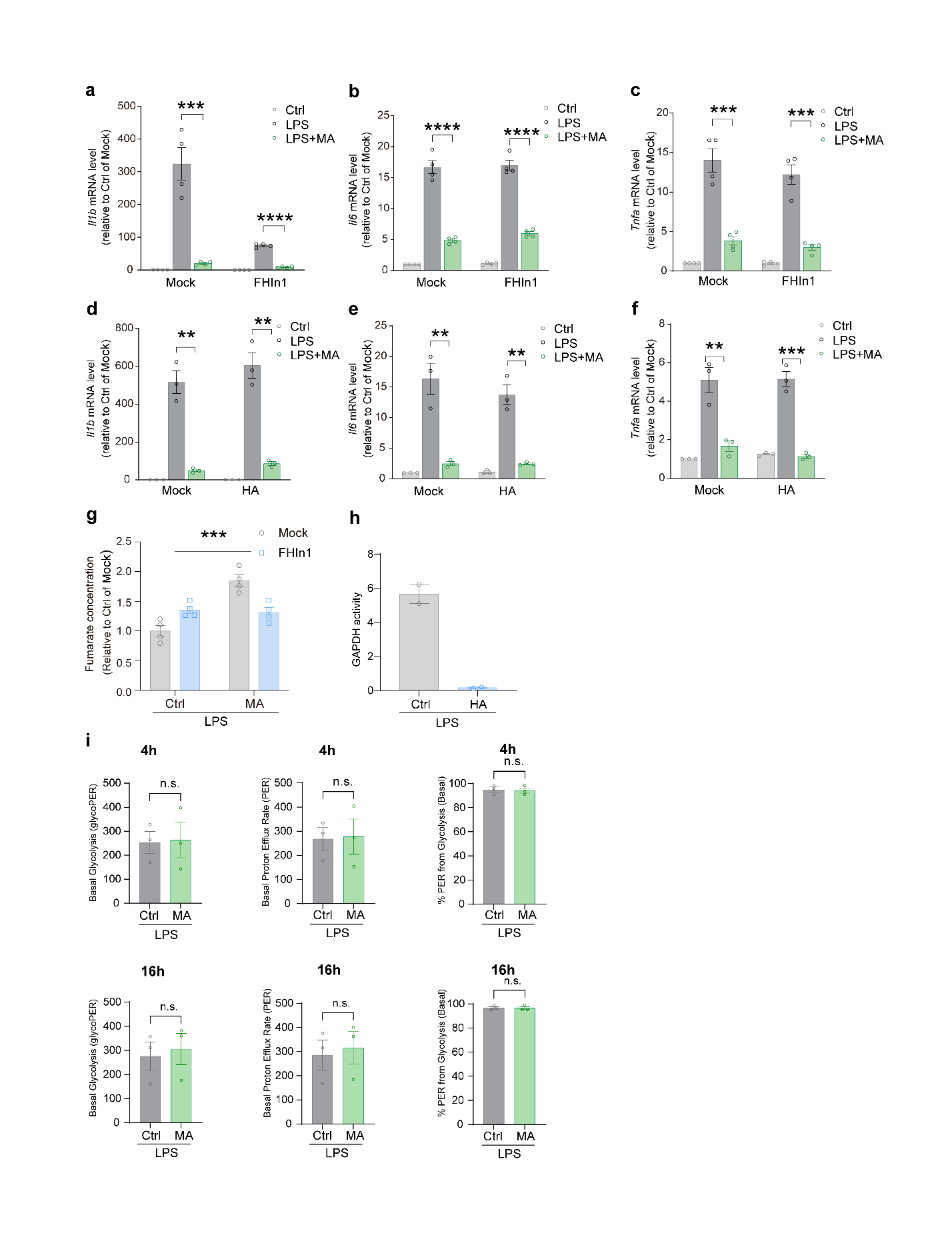


**Supplementary Figure. 6. L-malate inhibits inflammation independently of the metabolism-altering effect, related to Figure 2**

(a-f) *Il1b*, *Il6* and *Tnfa* expressions in FHIn1-treated (a - c) or Heptelidic acid (HA)-treated (d - f) BMDMs plus LPS and L-malate (24 h) (n ≥ 3).

(g) Fumarate levels of BMDMs treated with L-malate (6 mM, pH 6.7) in the presence or absence of FHIn1 under LPS stimulation (10 h) (n = 4).

(h) GAPDH activity of BMDMs treated with or without HA under LPS stimulation (16 h) (n = 2)

(i) Quantification of basal glycolysis (glycoPER), basal proton efflux rate (PER) and %PER from glycolysis in LPS-stimulated BMDMs treated with L-malate (6 mM, pH 6.7) for 4h and 16h (n = 3).

Data are shown as mean ± SEM. *p<0.05; **p <0.01; ***p <0.001; ****p <0.0001 (unpaired Student’s t test, two-way ANOVA).


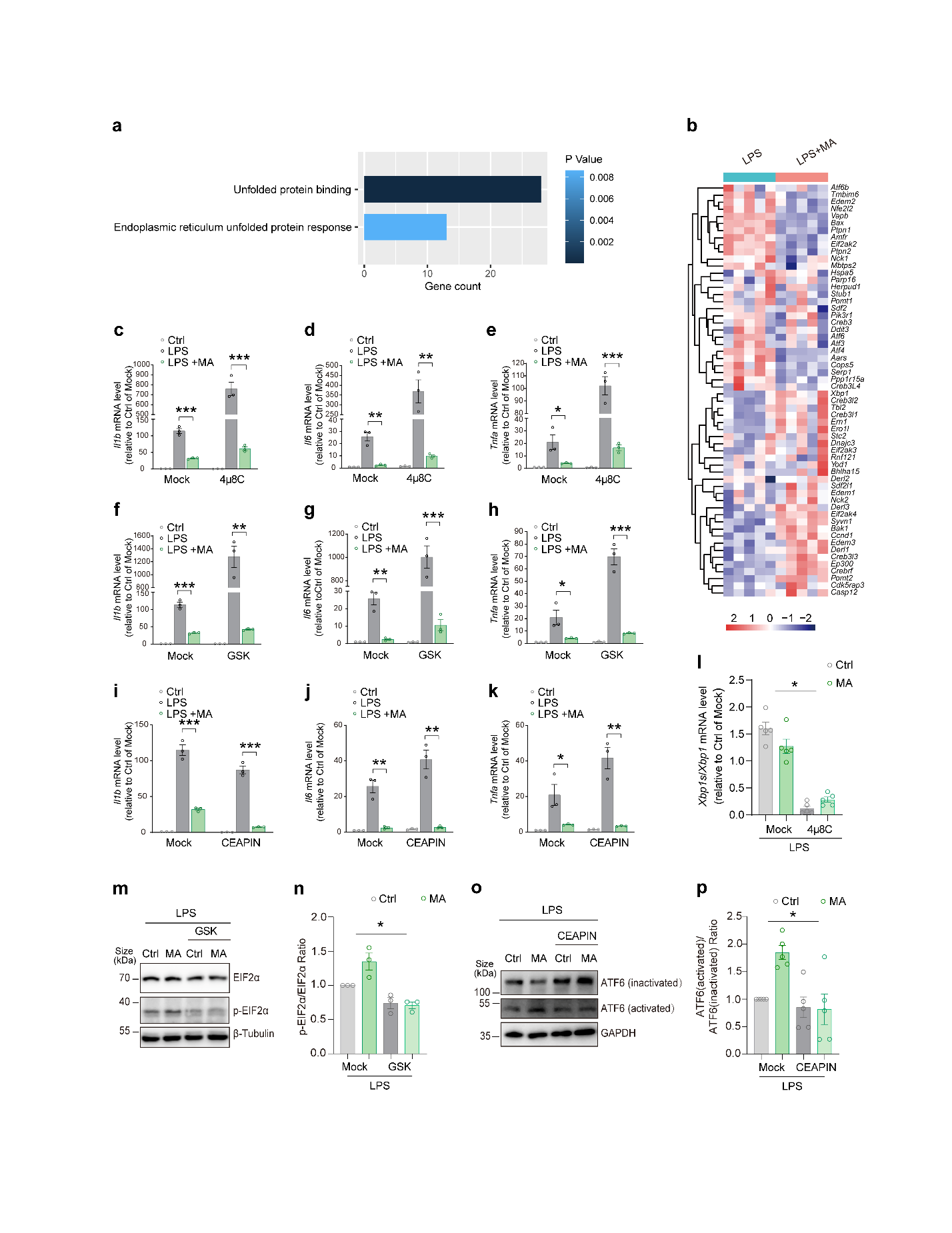


**Supplementary Figure. 7. L-malate inhibits LPS-induced macrophage activation independent of** **canonical UPR pathway****s, related to Figure 2**

(a) GO analysis (DAVID) of DEGs in BMDMs treated with LPS plus L-malate compared to LPS for 12 h, analyzed by RNA-sequencing, and UPR-associated pathways were shown.

(b) Expression of UPR-related genes in BMDMs treated as in (a).

(c-k) *Il1b*, *Tnfa*, or *Il6* mRNA expressions in LPS-stimulated BMDMs treated with/without L-malate (24 h) in the presence or absence of 4μ8C (c - e), GSK2606414 (GSK) (F-H) or CEAPIN-A7 (CEAPIN) (i - k).

(l) *Xbp1s*/*Xbp1* mRNA expressions in LPS-stimulated BMDMs treated with or without L-malate (6 mM, pH 6.7) in the presence or absence of 4μ8C for 24 h.

(m and n) Total and phosphorylated form of eIF2α protein levels in LPS-stimulated BMDMs treated with or without L-malate (6 mM, pH 6.7) in the presence or absence of GSK for 24 h.

(o and p) Inactivated and activated form of ATF6 protein levels in LPS-stimulated BMDMs mice treated with or without L-malate (6 mM, pH 6.7) in the presence or absence of CEAPIN for 6 h.

n ≥ 3. Data are shown as mean ± SEM. *p<0.05; **p <0.01; ***p <0.001; ****p <0.0001 (unpaired Student’s t test, two-way ANOVA).


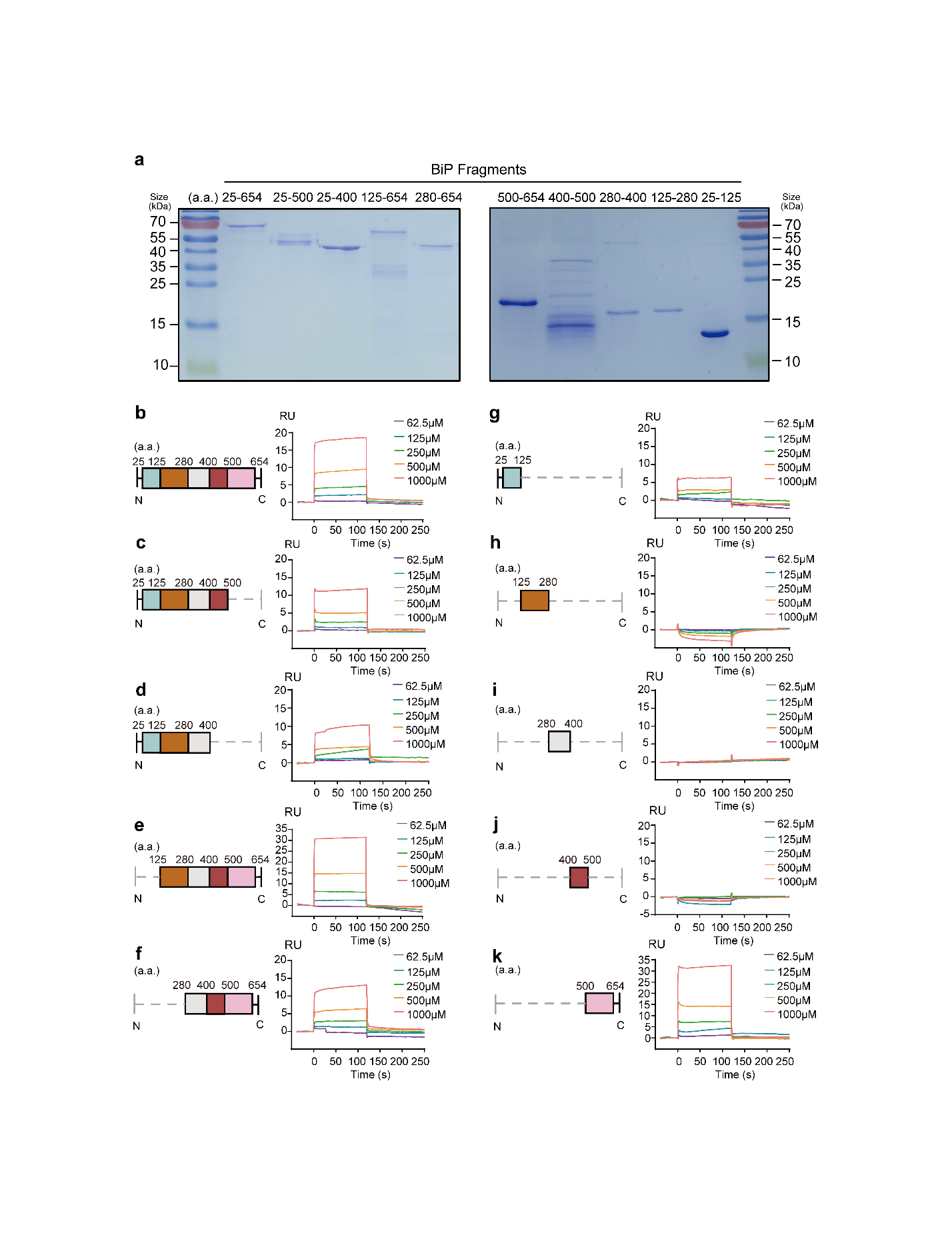


**Supplementary Figure. 8.** **L-malate bind the N terminal (aa 25 - 125) and C terminal (aa 500 - 654) domains of BiP, related to Figures 2 and 3**

(a) Coomassie blue staining of SDS-PAGE gel for analyzing BiP fragments.

(b-k) BIAcore diagrams of L-malate (concentrations indicated with colored lines, pH adjusted to 7.4) and E-Coli expressed BiP fragments (chip-coupled). Representative data from two experiments.


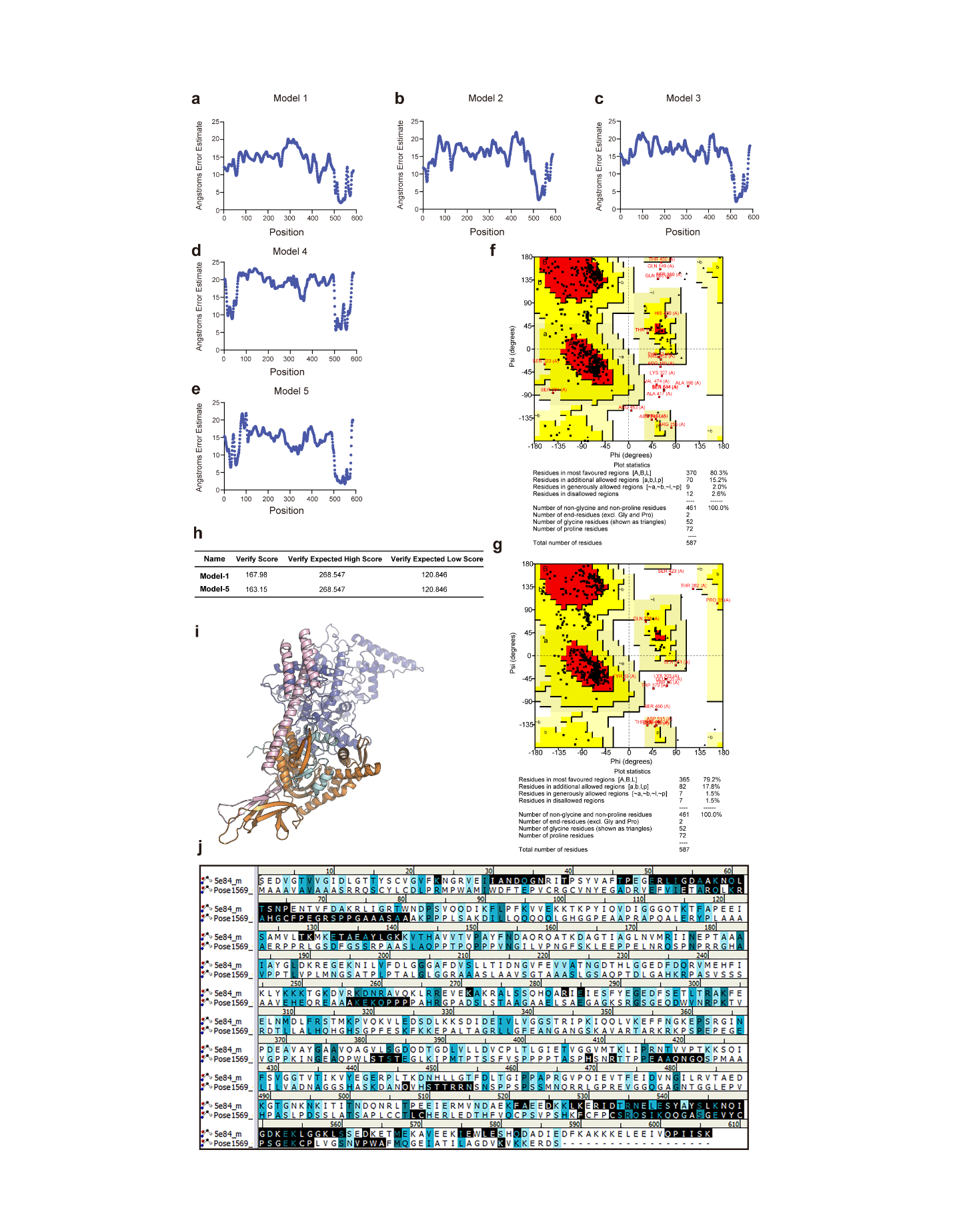


**Supplementary Figure. 9. Molecular modeling of IRF2BP2 and molecular docking of BiP-IRF2BP2 interaction, related to Figure 3**

(a-e) The angstroms error estimate values of model 1 (a), model 2 (b), model 3 (c), model 4 (d) and model 5 (e) predicted with RoseTTAFold.

(f, g) The Ramachandran Plot of Model 1 (f) and Model 5 (g).

(h) The Verify Protein (Profiles - 3D) results of Model1 and Model5.

(i) The binding model of BiP (crystal structure, PDE: 5E84) to IRF2BP2 (model1 predicted by RoseTTAFold) was predicted by protein-protein docking using ZDOCK. Different regions of BiP were respectively shown in palecyan (aa 1 - 125), tv_orange (aa 126 - 499), and palepink (aa 500 - 654), and IRF2BP2 was shown in blue.

(j) The selected amino acid residues were those involved in the interface of BiP-IRF2BP2 binding predicted by RoseTTAFold (5E84_m, the amino acid sequence of HSPA5; Pose1569, the amino acid sequence of IRF2BP2).


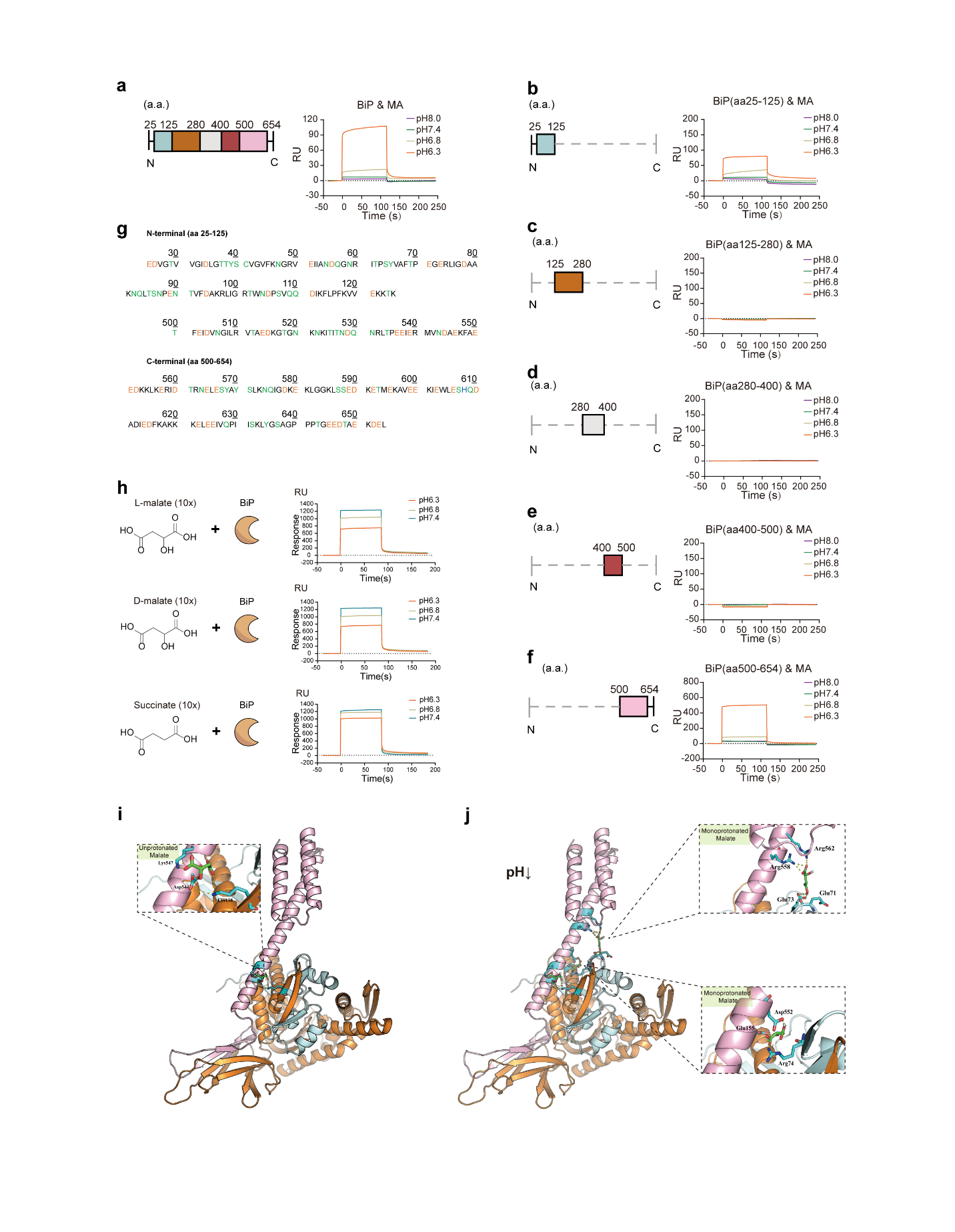


**Supplementary Figure.** **10. pH regulates direct binding between L-malate and BiP through dicarboxylate monoprotonation, related to Figure 7**

(a) BIAcore diagram of L-malate (1 mM) in different pH with E. coli-expressed BiP protein (chip-coupled). Representative data from three experiments.

(b-f) BIAcore diagram of L-malate (1 mM) in different pH with E. coli-expressed BiP fragments (chip-coupled), respectively. Representative data from three experiments.

(g) The amino acid sequence of N-terminal (aa 25 - 125), C-terminal (aa 500 - 654) regions in BiP with different types of amino acids colored by blue (histidine), orange (charged amino acids with -COO^-^ side chain), green (uncharged amino acids with -COO^-^ side chain).

(h) BIAcore diagram of L-malate, D-malate and succinate at concentrations of 10mM in different pH with E. coli-expressed BiP protein (chip-coupled). Representative data from two experiments.

(i, j) The binding models of BiP with the unprotonated malate alone (i) and the unprotonated malate plus the monoprotonated malate (j) were predicted by molecular docking. The malate was shown as stick colored by element (Carbon: green; Oxygen: red) while the related residues were shown as stick colored by element (Carbon: cyan; Oxygen: red; Nitrogen: blue). Protein-ligand interactions were represented with yellow dash lines.
